# Balanced contractility and adhesion drive polarization in a minimal elastic actomyosin network

**DOI:** 10.1371/journal.pcbi.1014750

**Published:** 2026-09-18

**Authors:** Zeno Messi, Franck Raynaud, Nathan W. Goehring, Alexander B. Verkhovsky

**Affiliations:** 1 Francis Crick Institute, London, United Kingdom; 2 Laboratory of Mechanisms of Cell Polarization and Cell Fusion, University of Geneva, Geneva, Switzerland; 3 Computer Science Department, University of Geneva, Geneva, Switzerland; 4 Department of Biochemistry, University of Oxford, Oxford, United Kingdom; 5 Laboratory of Biological Electron Microscopy, EPFL, Route de la Sorge, Lausanne, Switzerland; University of California Irvine, UNITED STATES OF AMERICA

## Abstract

Polarization of migrating cells involves chemical and mechanical interactions of signaling networks, cytoskeleton, plasma membrane, and substrate adhesions. Still, it is not fully understood which mechanisms and components are sufficient for symmetry breaking, and if they work independently or together. Here, we use a discrete active network model to investigate if and how an elastic cytoskeletal network is capable of breaking symmetry solely through mechanical interactions. Our minimal model consists of elastic bonds, attractive force dipoles, and force-sensitive anchor points, initially distributed uniformly and subject to simple turnover rules. We find that these features are sufficient to produce different cell behaviors, and, remarkably, to drive symmetry breaking and directed (polarized) motion. Network behavior was primarily determined by the turnover rate of anchor points, which, itself, is a function of the ratio between the dipole force and the threshold force required for anchor removal. Directional motion emerged at intermediate turnover rates, at which tension in the network accumulated through several turnover cycles before eventually exceeding the adhesion removal threshold locally at the edge, mirroring our recent experimental findings on the correlation of the traction force with protrusion-retraction transitions in the cell. At high turnover rates, forces were unable to build up to sufficiently high levels, while at low turnover rates, anchors hindered motion. These results demonstrate how directed motion can emerge as an intrinsic property of a simple mechanical network, independently of external cues or complex signaling networks. Given the concordance between this model and recent experimental findings, we suggest that polarization by contraction-adhesion dynamics could be a fundamental emergent behavior of actin-myosin networks.

## Author summary

From development to wound healing to immune responses, cell movement is essential, and to move, a cell must first decide where its “front” and “back” are, a process known as polarization. Most explanations for this behavior focus on complex chemical signaling inside the cell. In our work, we asked a simpler question: could mechanical forces within a cell be sufficient to make it polarize and move without an external cue? To explore this idea, we built on a computational model of a simplified cell made only of elastic connections, contractile forces, and attachment points to its surroundings. We started with a completely uniform system, without any built-in direction or external guidance. Surprisingly, we found that this minimal mechanical setup could spontaneously develop a front and a back and begin moving persistently. We proposed that the key mechanical factor controlling this behavior was the rate at which the attachment points break under mechanical load. When this process occurred at an intermediate rate, forces built up unevenly, leading to detachment and forward motion, similar to what is observed in living cells. Our findings suggest that cell polarization and movement can emerge spontaneously from mechanical properties alone, highlighting an important and overlooked role for mechanics in cell behavior.

## Introduction

Migrating cells can break symmetry, forming a structurally and functionally distinct protrusive front and retractive rear, and maintain this polarization during persistent motion. Polarization is a complex process involving the interaction of multiple components, including signaling networks, the plasma membrane, the cytoskeleton, and adhesions [1–7]. Although decades of biochemical and mechanical studies [8,9] have illuminated the properties and dynamics of individual players such as actin filaments, myosin motors, adhesion complexes, and the membrane, the integrative physical mechanisms initiating and subsequently stabilizing cell polarity remain unresolved. Polarization can occur in response to various external signals, such as chemical gradients, mechanical stimuli, and electric fields [10–15]. However, equally striking is that cells can polarize spontaneously, without an imposed directional cue, implying that symmetry breaking is an intrinsic property of the motile apparatus itself [16,17]. It could be initiated by localized protrusion at the prospective cell front, but also by the retraction at the prospective rear [18,19]. This suggests that polarization may occur via multiple pathways and that the mechanisms responsible for breaking symmetry may be distinct from those responsible for sensing external cues. Mathematical and computational models are pivotal to decipher this complexity by reformulating the problem in terms of simpler, individual, and testable processes that can reveal whether – and under what conditions – those components would spontaneously organize themselves to create front-rear polarity [20,21]. Several models attempted to include multiple, if not all, players of polarization processes, describing polarization in terms of feedback loops between the signaling networks, membrane, and the cytoskeleton [22–24]. These include reaction-diffusion dynamics of cytoskeletal regulators such as small GTPases [25], feedback between membrane tension or curvature and actin dynamics [26,27], coupling between polarity and motion through the flow of the actin network [28], as well as combinations of these mechanisms [24,29].

Models with a narrower focus have also addressed actomyosin mechanics using discrete filament-based or agent-based descriptions as well as continuum formulations [30–33]. Discrete models were particularly well suited to resolving filament-scale organization, motor activity, crosslinking, network architecture, and the effect of nonlinear actin elasticity on force transmission in a static isotropic configuration [34], but were not intended to reproduce symmetry breaking. Other studies have instead described the actin-myosin network as a viscoelastic continuum and investigated polarization [35–37]. Several types of feedback relationships and their combinations were considered, including non-linear feedback between actin flow and adhesions, force feedback from the outer boundary of the system, and competition between contractile and protrusive actin populations for a limited monomer pool. However, continuum models of polarization were not well suited to analyze the initial stages of symmetry breaking. They required manual introduction of either a significant initial asymmetry or very large fluctuations, with correlation length and decay time comparable to the dimensions of the whole simulation [35].

Here, we take a different approach: we extend a discrete elastic model of an actin-myosin network of an adherent cell to the dynamical case by adding simple rules of turnover. Most importantly, we introduce an intuitive rule for adhesion turnover: when adhesions experience a force above threshold, they break. This is inspired by our previous studies that demonstrate that increased traction forces at the cell periphery coincide with the cell edge switching from protrusion to retraction, eventually resulting in polarization [38,39]. In our minimalistic system of elastic bonds, force dipoles, and fixed substrate anchors, any feedback relationships and eventual symmetry breaking represent true emergent properties of local mechanics. We demonstrate that, provided the optimal balance between contractile and adhesive elements, this system can self-organize and break symmetry through solely mechanical interactions, without any feedback from chemistry, outer boundary, or mass conservation constraints. Such symmetry breaking in a contraction-adhesion system could represent a fundamental mechanism of polarization in adherent mesenchymal cells.

## Materials and methods

### Cell computational model

**Core:** We model the cell as a minimal actomyosin complex coupled to the substrate through adhesions. The actin cytoskeleton is represented by a network of elastic bonds attached at nodes on a hexagonal lattice. Each bond is made of two straight segments connected to each other via a hinge at the bond midpoint. The segments have a rest length $\ell_{0}$, they can be stretched and compressed, and the bonds can also bend at the hinge between the two segments (bond midpoint). A non-muscle myosin II minifilament is modeled by an attractive dipole attached to pairs of neighboring nodes. Note that it is not required that a bond underlies a dipole. Unlike [39], where adhesions were fixed nodes of the network, we rather attached the adhesion, hereafter called anchor, to a fixed spring to account for substrates with finite stiffness. The rest length of the anchor spring is set to 0. The system is initialized as follows:

1. *Node creation:* Nodes are placed on a hexagonal lattice (coordination number six), centered at the origin and restricted to a disk of radius 40 (in model units).
2. *Bond creation:* Each edge of the fully coordinated lattice is independently retained with probability ρ=0.7. Since this is well above the lattice bond-percolation threshold ($p_{c}\approx 0.35$), a spanning component always exists: we keep the largest connected component and discard all smaller components and isolated nodes.
3. *Anchor creation:* Each node of the remaining network is independently made an anchor with probability $\rho_{a}$ such that the density of anchors lies between 0.05% and 4% of the number of nodes.
4. *Dipole creation:* Finally, we place $N_{d}=\rho_{d}\;N_{node}$ dipoles, where $N_{node}$ is the number of nodes in the remaining network and $\rho_{d}=0.3$ is the dipole density. Each dipole is assigned to a pair of lattice-adjacent nodes drawn at random; a pair may be chosen even if it is not connected by a bond, and the same pair may receive more than one dipole.

Our model inherits largely from [34,39,40] in particular in its energetic and structural components. Previous works analyzed nonlinear elasticity and force transmission in a static and isotropic network [34] or in constrained geometry to show that traction forces increase with the distance from the cell center [39]. In the current study, we make the network dynamical, evolving it up to hundreds of minimization/remodeling cycles. More precisely, we introduced a force-sensitive adhesion turnover (anchors rupture above a load threshold), and network edge growth mimicking protrusion.

**Energy minimization:** The system evolves through several cycles of minimization of its energy, accounting for four contributions: stretching/compression of bonds, bending of bonds, dipole energy, and substrate deformation. Summing those contributions, the Hamiltonian reads (see Text A and Text D in S1 Appendix):


$$\begin{array}{c} ℋ=\underset{\text{spring energy}}{\underbrace{\mu_{b}\sum_{\text{segments}\;(i)}\frac{1}{2}{\left(\ell_{i}-1\right)}^{2}}}+\underset{\text{bending energy}}{\underbrace{\sum_{\text{hinges}\;(i)}2\sin^{2}\;\left(\frac{\phi_{i}}{2}\right)}}+\underset{\text{substrate deformation}}{\underbrace{\mu_{s}\sum_{\text{anchors}\;(i)}\frac{d_{i}^{2}}{2}}}+\underset{\text{dipole energy}}{\underbrace{M\sum_{\text{dipoles}\;(i)}l_{i}}}. \end{array}$$ (1)

As in [34,39] the Hamiltonian has been made dimensionless by setting to 1 the prefactor of the bending energy $2\frac{k_{B}T\ell_{p}}{\ell_{0}}$, with $\ell_{p}$ the persistence length of a filament [40]. Hinges are defined as midpoints of bonds, and points of contact between pairs of segments aligned in the initial underlying hexagonal lattice [34,39]. With this definition, bending penalizes angular deviation from the original alignment. In Eq 1, ℓ represents the segment length, ϕ the bending angle, *d* the length of the spring bound to the anchor, and *l* the distance between the two nodes forming the dipole (Text B and Fig A in S1 Appendix, and Table 1).

**Table 1:** Energetic parameters of the model (values and ranges are described in Text C, Text E, and Table A in S1 Appendix).

Name	Description	Value / range	Physical value
$\ell_{0}$	segment rest length (model units)	1	≈150nm\) [41]
$\mu_{b}$	dimensionless bond spring constant	1024	$100pN.nm^{-1}\backslash )$ [40]
$\mu_{s}$	dimensionless substrate spring constant	10^6^	$10^{5}pN.nm^{-1}\backslash )$
*M*	dimensionless dipole force magnitude	1--16	1--100 pN\) [42,43],

### Remodeling rules

**Pruning and merging:** Compared to previous studies [34,39,40], our computational cell model evolves through hundreds of consecutive energy-minimization procedures. Because no steric effects are included in the model, material would otherwise aggregate without bound and perturb the further computation and minimization; at each iteration we therefore apply a set of removal and merging rules that keep the discrete network well-behaved. These rules are motivated by identifiable features of actin-network disassembly, as detailed below.

1. *Force-dependent anchor removal:* The force applied on each anchor is computed, and the anchors withstanding a force higher than the threshold $T_{a}$ are removed. This represents the detachment of an adhesion whose transmitted load exceeds its rupture force.
2. *Buckling-induced severing:* A bond is removed when the interior angle between its two segments falls below $\alpha_{rem}=37^{\circ }$ (equivalently, when its deflection from straight exceeds $143^{\circ }$), i.e., when it buckles into a sharp fold under myosin contraction. The hinge energy $E_{bend}=2\sin^{2}(\phi /2)$ relates the deflection to a local radius of curvature $R=\ell_{0}/[2\sin(\phi /2)]$, so the threshold corresponds to $R_{rem}\approx 80\;nm$ (assuming $\ell_{0}\approx 150\;nm$). This threshold value is qualitatively consistent with buckled actin severing at radii of a few dozen [44] to a few hundred nanometers [45,46]. This rule is a conservative proxy removing the most acutely bent filaments.
3. *Density-limited node merging:* As previously mentioned, filaments are represented as one-dimensional bonds. Without volume exclusion nothing limits how much material the contracted network can draw through a single point. A per-node cap on the number of bonds and myosin links, together with the merging of nearby vertices, supplies the maximum local density that the finite filament diameter would impose in a real fiber network:
  - The vertices are iteratively grouped by pair and merged when the distance between them is smaller than a threshold distance $d_{merge}$. Each pair of merged nodes is replaced by a new node located at their barycenter, carrying all the bonds and dipoles of the pair; if one of the two merged nodes was an anchor, the new node is also an anchor.
  - A maximum of 10 bonds is fixed per node and 10 dipoles per pair of nodes. After merging, if more than 10 dipoles are attached to a pair, the exceeding dipoles are reassigned at random to other pairs of nodes, and the exceeding bonds are removed from the system.
  Because this cap acts precisely in the dense, compacted regions where myosin is known to fragment and sever F-actin [47,48], the material it removes mimics myosin-driven disassembly. The contribution of these disassembly modes is reported in Fig B and C in S1 Appendix.
4. *Removal of disconnected material:* Finally, the nodes that become disconnected, i.e., that do not belong to the largest connected component, are removed.

**Addition of material at the edge of the network:** A fundamental element to make the system dynamic is the addition of new material at the edge of the existing network. To do so, we first need to identify the boundary of the network. Since there is no reason for the shape to remain convex, typical algorithms for convex-hull detection are not reliable. Instead, we relied on a tracing algorithm using a point cloud to regularize outlines [49]. In brief, we consider all nodes that remain after the pruning procedure and compute the Delaunay triangulation of this set of nodes (if needed, fictive points are added at a distance of 0.01 around nodes to avoid singular edges in the triangulation). The boundary edges of the convex hull are directly identified from the triangulation, as they correspond to edges belonging to a single triangle; however, it is necessary to account for concavities. Edges of the convex hull are iteratively removed, and the convex hull recomputed after each removal, until no edges of the initial set with a length greater than 5 are present. This method [49] enables outlines with concavities and ensures that there are no holes within the shape. Then, each point of the outline is translated normally by a distance *P* to mimic actin protrusion at the edge of the cell. New material, including bonds, dipoles, and anchors, is added between the expanded and original outlines. The density of the new network components is the same as at initialization; new network bonds are then connected to the existing network if they are at a distance from the original network shorter than the rest bond length $\ell_{0}$. An iteration of the simulation is considered ended once the addition of new material is performed. Then the new iteration begins, the new Hamiltonian is computed and minimized using Broyden-Fletcher-Goldfarb-Shanno (BFGS) algorithm, forces are computed, and finally pruning and merging are performed as described previously (Table 2).

**Table 2:** Remodeling parameters.

Name	Description	Value / range	Physical value & Refs
	max bonds per pair of nodes	10	This study
	max dipoles per pair of nodes	10	This study
$T_{a}$	anchor removal threshold	0--16000	$\approx 10^{5}\;pN\backslash )$ [42,43],
*P*	protrusion rate	4	$\approx 100\;nm\;s^{-1}$ [50,51]
$d_{merge}$	node-merging distance	0.02	~3nm
$\alpha_{rem}$	interior angle for hinge removal	$\cos^{-1}(0.8)\approx 37^{\circ }$	[44–46]

**Shuffling of anchors and dipoles:** To test the effect of loss of anchor and/or dipole polarity, we considered a restarted simulation from a well polarized initial state randomizing anchors and/or dipoles. We simulated the system for 50 iterations and compared the trajectories and outlines obtained in the different conditions (control, shuffle dipoles, shuffle anchors, shuffle both).

**Simulations with external cue:** The external cue is simulated by varying the anchor threshold in the system. For any anchor, the effective removal threshold *T*_eff_ is given by a baseline $T_{a}$ shifted by a constant multiplied by the relative distance between the x-coordinate of the anchor and the center of the system


$$\begin{array}{c} T_{\text{eff}}=T_{a}+\frac{x_{a}-x_{c}}{\left|x_{\text{max}}\right|}D, \end{array}$$ (2)

where *D* is the gradient strength, $x_{a}$ and $x_{c}$ are the positions of the anchor and the center respectively. The distance is normalized by the distance to the anchor furthest from the center of the system.

**Model implementation and data analysis:** The model was implemented in a C++ program and the data was analyzed with C++ and Python programs. For energy minimization, the GNU scientific library implementation of the BFGS algorithm was used [52]. Computational geometry for finding and expanding the boundary of the system was carried out with the CGAL library [53].

### Quantification and statistical analysis

**Kymographs:** To obtain linear kymographs, we generated raster images of the system network, anchors, and dipoles at each simulation iteration. The resulting image sequences were imported into Fiji. For each sequence, we manually drew a line through the approximate center of the system, and used Fiji’s Reslice tool [54] to produce the kymograph. In polarized systems, the kymograph axis was aligned approximately with the front-back axis.

To construct circular kymographs, anchors and dipoles were radially projected onto a circle centered on the system centroid. At each iteration, the system was divided into 30 circular sectors radiating from this centroid (defined as the centroid of the system outline). In order to highlight eventual change of direction in the system, the orientation of the sectors was fixed with respect to the substrate frame of reference. Angular position zero points to the right (as in a trigonometric circle). The number of anchors (resp. dipoles) in each sector was counted and normalized to the maximum value at that iteration. The normalized values were then color-coded to generate a single line of the kymograph.

**Network flow:** Network flow was quantified as the average displacement of nodes before and after the minimization procedure. The system was divided into a 40×40 grid, scaled to the system size (with the number of squares fixed and their dimensions varying accordingly). The vectorial displacements of all lattice nodes were averaged in each square and represented by an arrow. Arrow length and color encode the magnitude of the average displacement.

**Anchor lifetime:** To quantify anchor lifetime, we counted how many iterations anchors existed for before being removed. Every anchor in the system was followed from creation to removal or to the end of the simulation. If an anchor was not removed at the end of the simulation, its current lifetime was taken into account.

**Trajectory gyration radius:** To quantify the persistence of motion, we measured the gyration radius of trajectories. Each trajectory was treated as a point cloud, and its gyration radius was computed as the root-mean-square distance of all trajectory points from the cloud’s center of mass


$$\begin{array}{c} R_{g}=\sqrt{\frac{1}{N}\sum_{i}{\left(r_{i}-r_{m}\right)}^{2}}. \end{array}$$ (3)

Compared to persistence measures based on end-to-end distance, the gyration radius incorporates the entire trajectory, and remains meaningful even when trajectories include pauses, loops, or changes of direction.

**Force maps:** Force maps were generated from raster images of the forces acting on anchors. The system was divided into a grid of 517×517 pixels. For every pixel in the grid, the assigned force was the average of contributions from all nearby anchors, up to a distance of 40 length units. This approach does not represent a physical force field, but rather provides a qualitative visualization of the spatial distribution and relative magnitude of forces. In addition to the force image, we created three other images for the trajectory, system outline, and the force colormap. All four images were imported into Fiji, where the force image was blurred and then blended with the other layers to produce the final visualization.

**Radial histograms:** For simulations with an external cue and migrating phenotype, radial histograms were generated from the angular distribution of the end-to-end vectors, pooled across 20 simulations per parameter set. For the radial phenotype, histograms were instead computed from the angular distribution of all displacements recorded during the simulations. In both cases, bins represent a 30-degree angle.

**Straightness of normalized trajectory:** To compare the trajectories of cells of different sizes, we define a metric that decouples the straightness of the trajectory from speed and size. This metric is computed as follows: first, we calculate the mean squared displacement MSD(τ) for different lag values τ:


$$\begin{array}{c} MSD(\tau )={\left\langle {\left|{\hat{𝐫}}_{n+\tau }-{\hat{𝐫}}_{n}\right|}^{2}\right\rangle }_{n} \end{array}$$

Here ${\hat{𝐫}}_{n}=\sum_{i=0}^{n-1}\delta {\hat{r}}_{i}$ and $\delta {\hat{r}}_{i}=\frac{\vec{P_{i}P_{i+1}}}{\left\|\vec{P_{i}P_{i+1}}\right\|}$ is a normalized vector pointing along the direction of two successive positions of the geometrical cell center $P_{i}$ and $P_{i+1}$ (Fig D in S1 Appendix). In this representation, the MSD of ballistic motion and that of two-dimensional random motion bound the MSD of any simulated trajectory. For a ballistic motion, we have $MSD_{ballistic}(\tau )=\tau^{2}$, while for a random motion $MSD_{random}(\tau )=\tau$. The straightness of the normalized trajectory, SNT\), is calculated as:


$$\begin{array}{c} SNT=\frac{{𝒜}_{\text{sim}}^{\text{MSD}}-{𝒜}_{\text{random}}^{\text{MSD}}}{{𝒜}_{\text{ballistic}}^{MSD}-{𝒜}_{\text{random}}^{\text{MSD}}} \end{array}$$

where ${𝒜}^{MSD}$ represents the area under the MSD curves for the simulated cells, ballistic motion, or random motion. Consequently, if the cell trajectory is a straight line, SNT=1\), whereas if the trajectory is random, SNT=0\).

## Results

### A mechanical model of self-organized cell migration

The core of the model is based on a network of elastic elements, bonds, connected via nodes, effectively representing a minimal actin network [34,39]. Similar to actin filaments, bonds can undergo stretching, compression, and bending when subjected to force. To mimic asymmetric elasticity of actin filaments, parameters of the model are set such that bending requires significantly less force than stretching or compression (see Materials and methods). The two additional mechanical elements are attractive force dipoles that exert contractile force between neighboring pairs of nodes, reflecting myosin, and anchors, which take the form of nodes attached to fixed, zero rest length springs and mimic substrate attachment sites with a finite stiffness. Importantly, the anchors are force sensitive – if the applied force exceeds a threshold, the anchors are removed.

To explore the behavior of this system, networks were initialized on a hexagonal lattice with random distributions of bonds, dipoles, and anchors (Fig 1A). Networks were then evolved as follows. They were allowed to deform to find a conformation of minimal energy, and anchors experiencing force above threshold were removed. The network was then pruned. Briefly, highly bent bonds were removed, and pairs of nodes closer than a threshold distance were merged. Merging could result in multiple bonds and dipoles connecting the same nodes. If there were more than 10 dipoles and/or bonds connecting a pair of nodes, excess dipoles were reassigned randomly, and excess bonds were removed (Fig 1B, see Material and methods for details). Finally, the edge of the system was extended isotropically, mimicking actin polymerization, with new bonds, dipoles and anchors, and the network was allowed to deform again to find a new force-free conformation of minimal energy (Fig 1C and Fig E in S1 Appendix). This sequence was repeated at each iteration. The simulations were stopped after 400 iterations or if the system exceeded a threshold area of 100000 model units.

**Fig 1:**
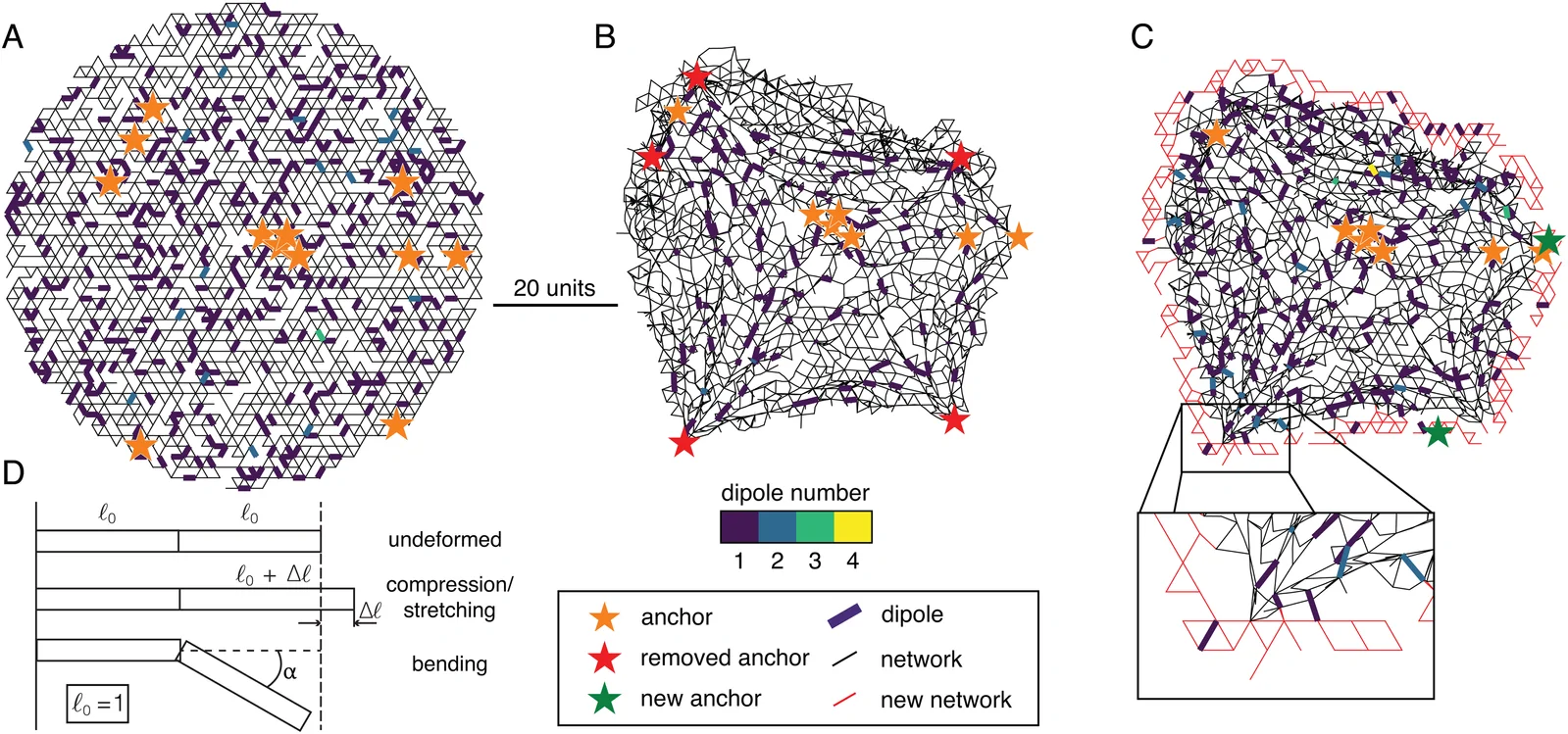
Model description and dynamical rules. A-C) State of the system at different stages of an iteration of the simulation. Bonds and anchors are represented as black segments and orange stars, respectively. The number of dipoles connecting the same network node is color-coded from dark blue (low number) to yellow (high number). Scale bar represents 20 model distance units. A) State of the system at initialization, in a circle of 40 units radius with uniform distribution of bonds, anchors and dipoles. B) State of the system after energy minimization. After minimization, anchors that are subjected to a force exceeding force threshold (in red) are removed. Only dipoles with a non-zero extension are displayed. C) State of the system after merging of neighboring nodes, pruning of highly bent bonds, and addition of new bonds (red) and anchors (green). D) Description of the three modes of bond deformation: stretching, compression and buckling under load. The parameters for the simulation are: anchor density $\rho_{a}=1$, dimensionless dipole force magnitude *M* = 8, anchor removal threshold $T_{a}=40$.

### Assessing system size and motion across model parameters

As previously described, the system evolves through an iterative process: isotropic addition of new material along the perimeter, followed by energy minimization. We first investigated the mean area and its temporal evolution across an ensemble of over 150 distinct parameter sets (Fig 2A). Those parameter sets correspond to different anchor density, anchor removal threshold and dipole force, to investigate the respective effects of the number of anchors in the system, the strength of the adhesion and the strength of the contractile units. Since material addition in our model is unconstrained by design, we anticipated unbounded area growth. We observed this behavior in half of the parameter sets (Fig 2A insets). While unconstrained growth is of limited biological relevance, it is notable that the remaining parameter sets produced areas that stayed below a prescribed threshold. Most cells maintained their area below this limit and stabilized at a stationary area over time, rather than merely growing more slowly (Fig 2A insets and Fig F in S1 Appendix). Importantly, the temporal evolution of the different network components closely parallels that of the cell area, indicating that the systems are not getting denser over time (Fig G in S1 Appendix).

**Fig 2:**
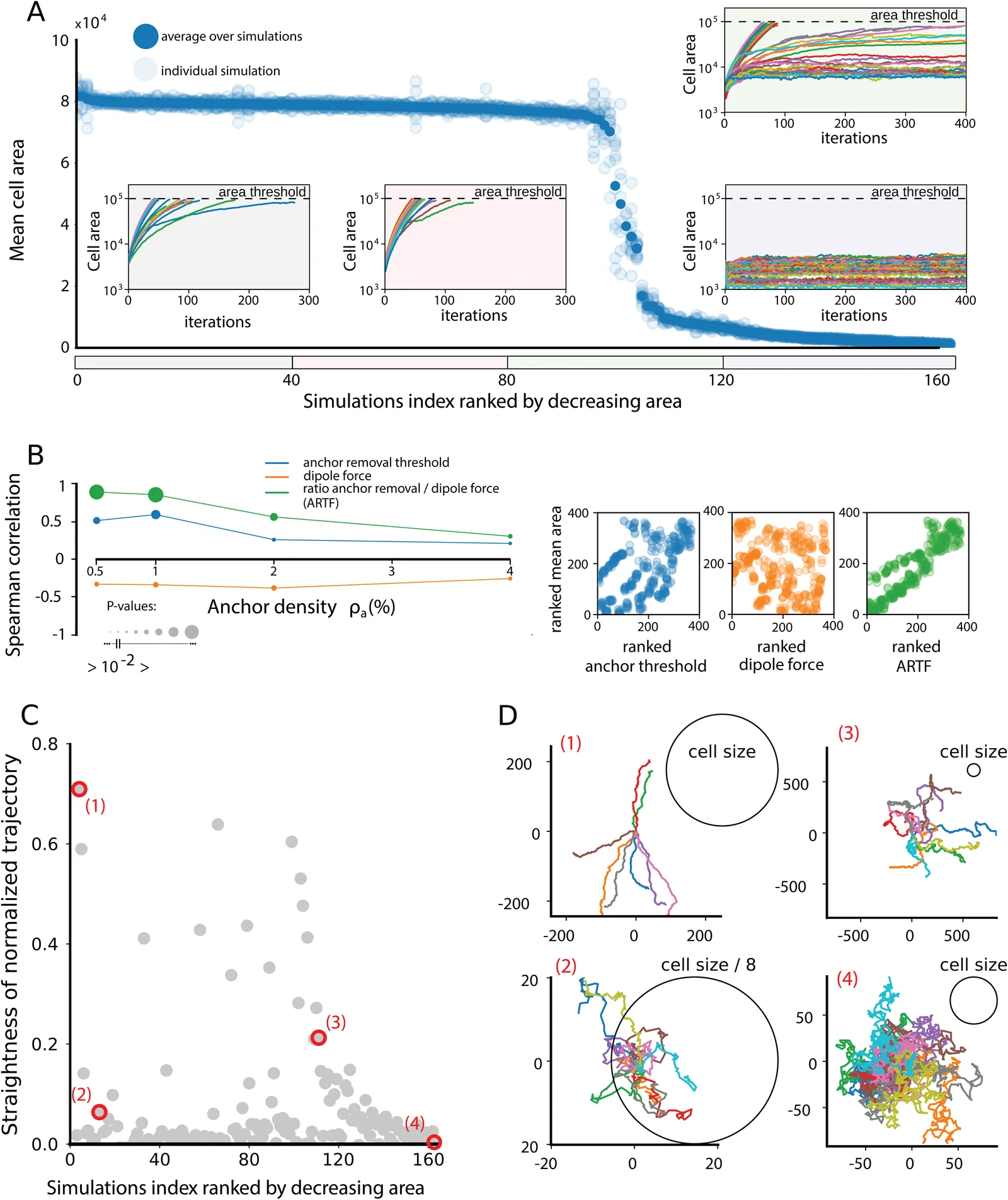
Model behavior. A) Mean cell areas plotted against the simulation index, ranked by decreasing area. Transparent blue points represent individual simulations, while solid blue points denote the average over identical parameter sets. For each simulation, the mean area is computed over the final third of the simulation duration. Insets show the temporal evolution of cell area, averaged across identical parameter sets; each inset corresponds to a group of 40 distinct parameter sets. B) (Left) Spearman correlation coefficients between cell area and anchor removal threshold (blue), dipole force (orange), and ARTF (green) as a function of anchor density. (Right) Scatter plot of ranked mean cell area versus ranked anchor removal threshold (blue), ranked dipole force (orange), and ranked ARTF (green) for a fixed anchor density $\rho_{a}=0.5\%$. C) Scatter plot of the SNT, ordered by decreasing mean cell area. Four red circles are representative cases: large cells with high (1) and low (2) SNT, and small cells with high (3) and low (4) SNT. D) Cell trajectories corresponding to the four cases marked in C (1–4). Trajectories are non-normalized. Each trajectory represents one realization of a given set of parameters. The circle represents one typical cell size for (1), (2), and (4); for (3), the circle represents 1/8 of the cell size.

We then investigated which parameters and parameter combinations determine cell size. For each simulation, we first assessed the associations between cell area and both the anchor threshold and the dipole force using Spearman rank correlations (Fig 2B). Across all anchor density values, cell area showed a significant positive correlation with anchor threshold and a significant negative correlation with dipole force. Accordingly, the ratio of anchor removal threshold to dipole force (ARTF) exhibited the strongest association with cell area, an association that weakened as anchor density increased.

Next, we examined the temporal characteristics of cell center displacement. Monitoring displacement alone is insufficient for comparing cells of different sizes, as two simulated cells can exhibit vastly different net displacements solely due to size differences. A more informative approach is to assess the straightness of the cell motion. To enable comparison across cells of varying sizes, we defined a straightness based on the mean squared displacement (MSD) of a normalized trajectory of the cell center (see Materials and methods). This metric facilitates the comparison across all parameter sets independent of cell size and instantaneous velocity, avoids explicit curve fitting for MSD(τ), and penalizes persistence at short time scales.

Fig 2C plots the straightness of the normalized trajectory, SNT, for all simulations, ordered by decreasing cell area. Both large and small cells span the full range of SNT, and in each case the two extremes correspond to two distinct behaviors, made apparent by inspecting individual trajectories (Fig 2D). Among the small cells, low SNT reflects erratic motion and high SNT reflects persistent migration. Among the large cells, low SNT corresponds to nearly immobile cells, whereas high SNT corresponds to a small but straight centroid displacement: the trajectory is directionally straight, but its magnitude is small relative to the cell size and arises from directed, growth-driven translocation of the centroid rather than from locomotion. Straightness therefore captures the shape of the trajectory, not the distance traveled, which is why a large cell can score high SNT while barely displacing relative to its size. The following section characterizes these four behaviors in detail.

### Types of system behavior dependent on anchor turnover parameters

The ARTF ratio and the anchor density determined how often the anchors were removed by the forces built in the network. Variation of these parameters produced five phenotypes: erratic, centripetal, isotropic-growth, anisotropic-growth, and migrating, which we describe in three groups according to their underlying contraction/adhesion dynamics.

#### Erratic and centripetal.

For low ARTF ratios (0–3) nearly all anchors are removed at each iteration. Resulting configurations of the network, evolution of system outlines, and motion and distribution of the elements are illustrated in Fig 3. If the anchor density parameter is low, the system shrinks from its initial size and remains small. At any iteration, there are only a few anchors, and they are removed immediately, leading to erratic contractions and jittering motion of the system (S1 Movie). Higher anchor density leads to a larger system with anchors deposited and subsequently removed in a band at the periphery, resulting in a more organized contraction in a centripetal direction and radial organization of the network (see network organization and vector maps of flow (Fig 3G and 3L and S2 Movie)). Both erratic and centripetal systems are characterized by concentration of dipoles near the system center and absence of anchors anywhere except the very periphery (Fig 3). To further illustrate the dynamics of these systems, we generate kymographs of two types: linear and circular. Linear kymographs along an arbitrary line (Fig 3E and 3K) show contractions at the periphery as well as general erratic motion of the system and demonstrate that anchors at the periphery of the system are short-lived. To create circular kymographs, we radially projected anchors and dipoles at each iteration onto a circle drawn around the system and displayed linearized circles side by side in a time sequence (Fig 3C and 3I). Circular kymographs of both erratic and centripetal systems show that anchors and dipoles are distributed in nearly isotropic fashion over the time sequence. The centripetal system is reminiscent of cells with weak adhesions displaying strong isotropic retrograde flow [55,56].

**Fig 3:**
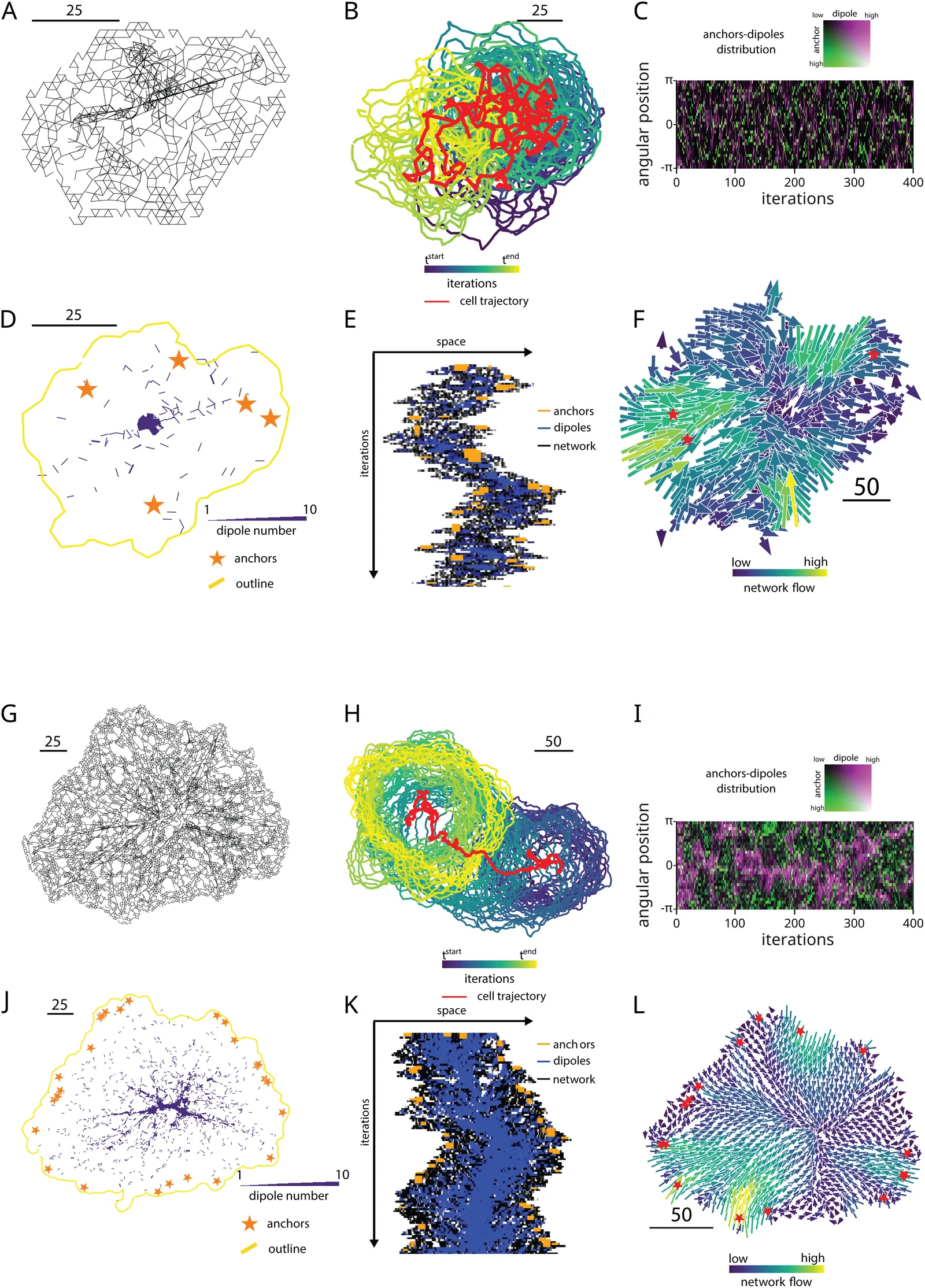
Low ARTF ratio leads to erratic motion and fast centripetal flow. Features and characteristic movement of systems with low ARTF ratio, and low anchor density (A-F) or high anchor density (G-L). A,G) Network configuration. B,H) Evolution of the cell contour over 400 iterations. The position of the centroid is drawn in red. The system outline is shown every 10 iterations, color-coded for time from dark blue (first iteration) to yellow (last iteration). C,I) Circular kymograph of dipoles (green) and anchors (red) (see Materials and methods). D,J) Spatial distribution of anchors (orange stars) and dipoles (blue) with the system outline (yellow). The width of dipoles represents the number of overlapping dipoles. E,K) Kymograph along a line going through the approximate center of the system along a random axis. Bonds are represented in black, dipoles in blue, and anchors in orange. F,L) Network displacement before and after minimization. Amplitude of the displacement is color-coded from dark blue (low flow) to yellow (high flow). The scale bar is given in model distance units. Anchors removed before the last minimization are shown in red. The parameters for the simulations are: (A-F) anchor density $\rho_{a}=1$, dimensionless dipole force magnitude *M* = 16, anchor removal threshold $T_{a}=16$, (G-L) anchor density $\rho_{a}=2$, dimensionless dipole force magnitude *M* = 3, anchor removal threshold $T_{a}=7.5$.

#### Isotropic and anisotropic growth.

For very high ARTF ratios (10 and above), the force generated in the network never reaches the anchor threshold, and anchors are not removed irrespective of their density. This phenotype is characterized by round systems undergoing isotropic growth. Growth is unlimited because our model does not feature mass conservation. These systems never move; their center of mass remains within the area occupied by the first iteration (S3 Movie, Fig 4A – 4F). Only the peripheral region of the system, where new network was recently added, undergoes some rearrangement and pruning (Fig 4F, S3 Movie). The rest of the system is frozen (Fig 4E and 4F). Rearrangements at the periphery during growth of the system later become frozen in the form of small alternating regions of high and low density of bonds and dipoles with anchors spread uniformly over the area. Overall distribution of all elements in the system is approximately uniform and isotropic (Fig 4C). Such systems with a narrow peripheral dynamical region and frozen bulk are similar to cells spreading under conditions of impaired contractility.

**Fig 4:**
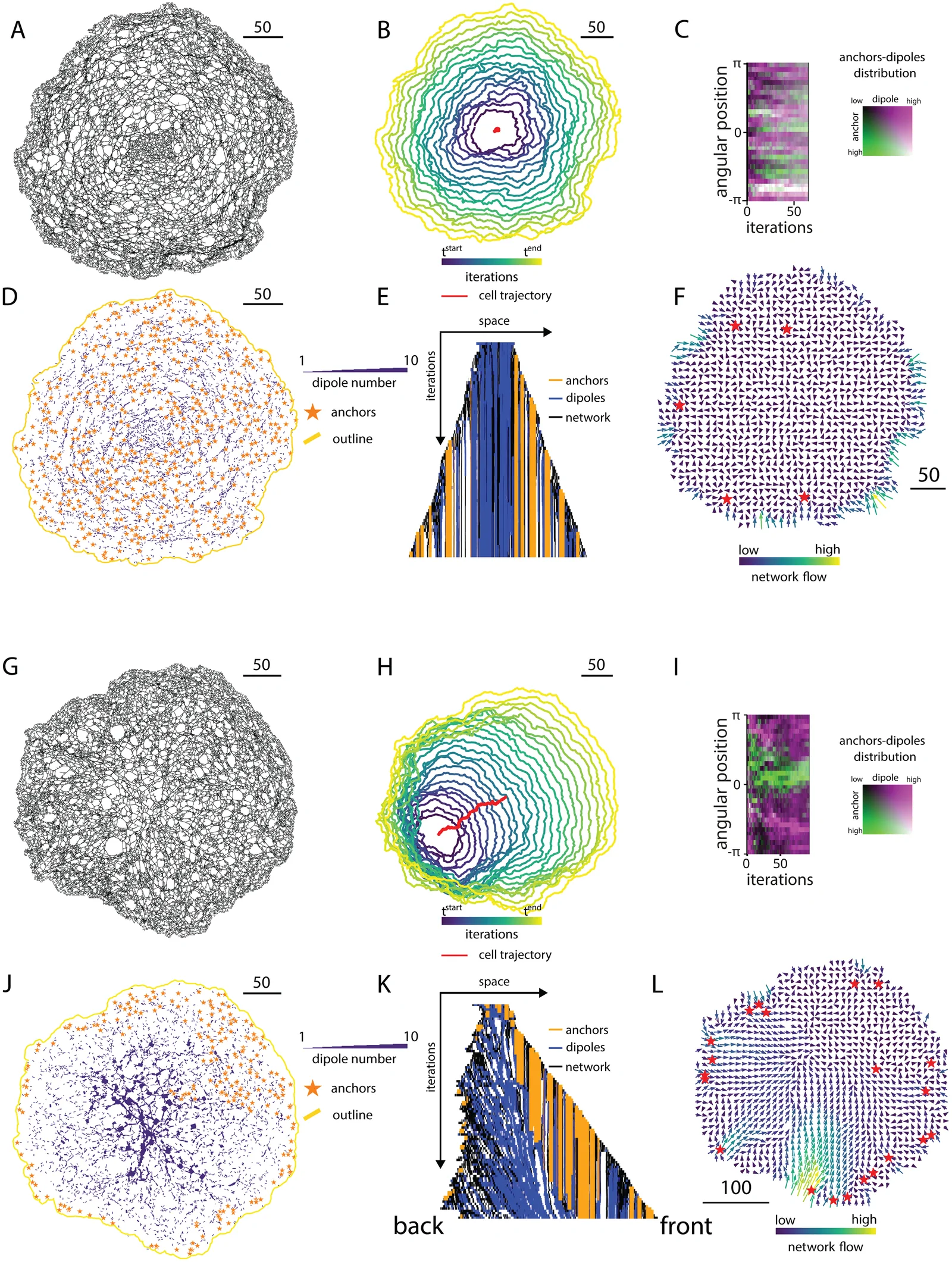
High ARTF ratio leads to system growth. Features and characteristic movement of systems with high ARTF ratio. A,G) Network configuration. B,H) Movement of the system throughout the simulation (400 iterations). The position of the centroid is drawn in red. The system outline is shown every 10 iterations, color-coded for time from dark blue to yellow (viridis palette). C,I) Circular kymograph of dipoles (green) and anchors (red) (see Materials and methods). D,J) Distribution of anchors (orange stars) and dipoles (blue) with the system outline (yellow). The width of dipoles represents the number of overlapping dipoles. E,K) Kymograph along a line going through the approximate center of the system along a random axis (E) and along the direction of growth (K). Network is represented in black, dipoles in blue, and anchors in orange. F,L) Network displacement before and after minimization. Amplitude of the displacement is color-coded from dark blue (low flow) to yellow (high flow). The scale bar is given in model distance units. Anchors removed before the last minimization are shown in red. The parameters for the simulations are: (A-F) anchor density $\rho_{a}=1$, dimensionless dipole force magnitude *M* = 8, anchor removal threshold $T_{a}=80$, (G-L) anchor density $\rho_{a}=2$, dimensionless dipole force magnitude *M* = 4, anchor removal threshold $T_{a}=20$.

Lowering the ratio of anchor removal threshold to dipole force (typically, between 5 and 10) results in systems that also display unlimited growth, but the growth now has a preferred axis (Fig 4, S4 Movie). These systems do not move in the sense that the resulting trajectory never leaves the area occupied by their original contour, but they display a striking polarization. One side of the system grows steadily, while the other remains largely in place (Fig 4H). At the growing side, the network is frozen in the bulk and the anchors remain throughout the simulation, while the opposite side undergoes cycles of anchor removal and contraction and displays a flow of the network away from the periphery (Fig 4K and 4L). Thus, the edge of the contracting side maintains its position despite the continuous addition of new network. This outcome can be explained as follows: once a few anchors are removed locally, the force load on the anchors remaining in the vicinity increases, making their removal in the following cycles more likely than in other areas. At the same time, anchor removal leads to the collapse of the network, concentrating dipoles and further increasing the force load. These mechanical events effectively generate a mutual negative feedback between adhesion and contraction – a mechanism postulated in several previous studies [35,57]. Removal of the adhesion leads to contraction, which in turn, facilitates further adhesion removal. Consistent with this interpretation, across all phenotypes an adhesion was more likely to fail when located near one that had just detached (Fig H Panel A in S1 Appendix), suggesting that local force redistribution destabilizes neighboring adhesion sites. As shown in Fig H Panels B and C in S1 Appendix, the load released by the loss of one anchor is transferred to nearby anchors, bringing them closer to their own detachment threshold. Adhesion turnover therefore appears to be a spatially coordinated process in which the loss of one adhesion redistributes force to neighboring adhesions, rather than a sequence of independent events. The organization of this process is phenotype-dependent (Fig H Panel D and Text G in S1 Appendix): failure events are strongly clustered in migrating and anisotropic-growth cells, remain more dispersed in the centripetal regime, and are close to random in erratic cells. Together, these observations suggest that the balance of adhesion strength, contractile force, and adhesion density determines whether this feedback gives rise to localized cascades of adhesion turnover or to a more diffuse pattern, thereby helping to explain the distinct migratory and growth behaviors observed. Our simulations demonstrated that stochasticity of local dynamics eventually leads to self-organized growing and stable edges segregated to opposite sides of the system, producing polarized distributions of dipoles and anchors. Dipoles become concentrated towards the interior of the system, with the region of highest density shifted to the contracting side, while the anchors are present along the entire perimeter, but are internalized into the bulk only at the growing side where they are not removed (Fig 4J and 4L).

#### Migrating.

Finally, a migrating phenotype is observed at intermediate ARTF ratios between 3 and 10, depending on the anchor density (lower ARTF ratios require higher anchor density). In contrast to growing phenotypes, the size of these systems reaches a steady state, and they move persistently several diameters before eventually changing direction (Fig 5B, S5 Movie). The dynamics of these systems is similar to anisotropic growth: anchors are removed at one side (trailing edge) and persist at the other side (leading edge), the network displays strong inward flow at the trailing edge and low and disordered flow at the leading edge, and the distribution of dipoles and anchors is strongly polarized. Unlike anisotropically growing systems, contraction at the trailing edge not only balances the addition of new network but leads to a steady translocation of the whole system [58,59]. Despite uniform addition of network and anchors everywhere at the periphery, anchors added at the back are quickly removed and the system robustly maintains its polarity (Fig 5F, inset). The shape and dynamics of these systems bear a strong resemblance to persistently migrating cells like fish and amphibian keratocytes: the system is elongated perpendicularly to the direction of motion, the leading edge is convex, and the trailing edge, is concave. The network and dipoles are concentrated in a bundle-like structure running parallel to the trailing edge (Fig I in S1 Appendix); anchors are found at the front and sides, and dipoles at the back. Similar to a keratocyte, the system advances steadily at the front while displaying variable edge velocity at the back characterized by short-lived protrusions followed by rapid retraction [38,60] (Fig 5F, inset).

**Fig 5:**
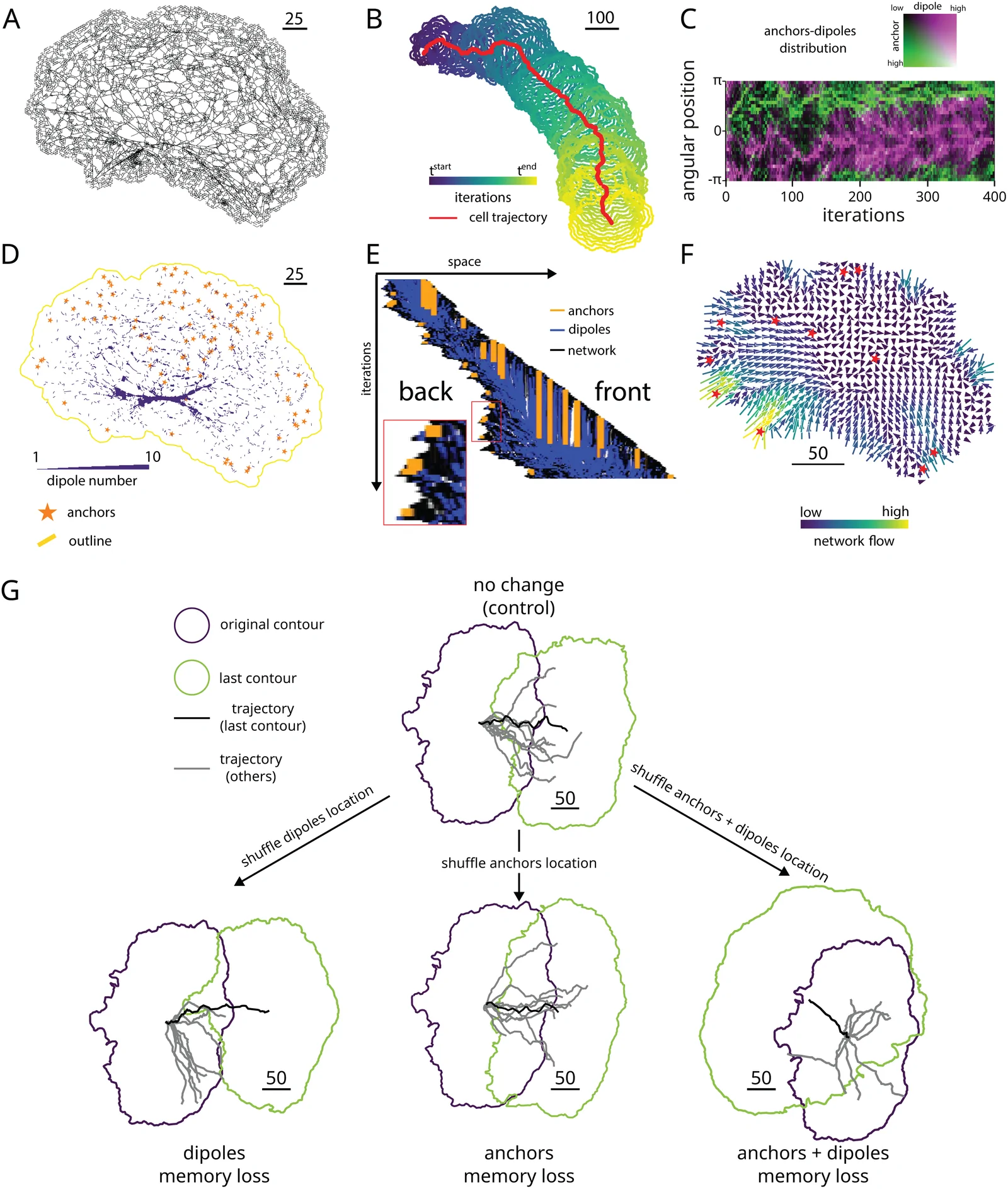
Intermediate ARTF ratio leads to spontaneous polarization and persistent migration. Features and characteristic movement of a system with intermediate ARTF ratio. A) Network configuration. B) Movement of the system throughout the simulation (400 iterations). The position of the centroid is drawn in red. The system outline is shown every 10 iterations, color-coded for time from dark blue to yellow (viridis palette). C) Circular kymograph of dipoles (green) and anchors (red) (see Materials and methods). D) Distribution of anchors (orange stars) and dipoles (blue) with the system outline (yellow). The width of dipoles represents the number of overlapping dipoles. E) Kymograph along a line going through the approximate center of the system along the direction of migration. Network is represented in black, dipoles in blue, and anchors in orange. Inset: Zoom on the back of the cell showing multiple short-lived protrusion-retraction events. F) Network displacement before and after minimization. Amplitude of the displacement is color-coded from dark blue (low flow) to yellow (high flow). Anchors removed before the last minimization are shown in red. G) Effect of randomization of anchor and/or dipoles on a polarized system. Top panel shows 20 simulations of the control case (without any randomization) with a representative initial (resp. last) configuration in blue (resp. green) and the corresponding cell trajectory in black. Other trajectories are displayed in gray. The three lower panels represent respectively spatial perturbation of dipoles (left), anchors (middle) and both dipoles and anchors (right). The scale bar is given in model distance units. The parameters for the simulation are: anchor density $\rho_{a}=1$, dimensionless dipole force magnitude *M* = 8, anchor removal threshold $T_{a}=60$.

To probe the mechanism of polarization in the migrating phenotype, we tested if the direction of motion is determined by the polarized distribution of anchors, dipoles, or both. To this end, at one of the iterations of the simulation where the system displayed directional motion, we randomized the distribution of either anchors, or dipoles, or both and ran multiple realizations of the simulation for each case. Resulting trajectories were compared to multiple realizations of the same simulation without randomization of the elements. We observe that after randomization of just one type of the elements, the system displays a directional bias indistinguishable from the system without randomization, and only the randomization of both anchors and dipoles results in a loss of directionality (Fig 5). This result is consistent with the idea of a feedback between adhesion and contraction as described above.

### Intermediate anchor lifetime is optimal for motion

The above observations suggest that anchor turnover is important for the type of system behavior. To characterize anchor turnover in quantitative terms, we measure mean anchor lifetime and investigate how it depends on the ARTF ratio and anchor density (Fig 6A). Anchor lifetime increases rapidly with the ARTF ratio, and the increase happens earlier with higher anchor density. These results can be explained as follows. Over multiple iterations, bonds and dipoles tend to form bundled structures in which several dipoles are aligned. These structures, reminiscent of stress fibers, are generally found between anchors. The stacking of actively pulling network elements in parallel leads to an increase in the force on the anchor that eventually reaches its threshold and the anchor is removed. The ARTF ratio sets the minimal number of dipoles that must pull on a single anchor to remove it. As a result, the anchor lifetime is correlated with this ratio; the greater the ratio, the more dipoles are required to remove an anchor and therefore the more steps are required to build a structure with sufficient pulling force. The anchor density has an impact on the anchor lifetime as well: a larger density implies that the force is distributed among a larger number of anchors, and it takes longer to reach the threshold. Eventually, at a high ratio of anchor removal threshold to dipole force, large structures are not able to remove anchors fast enough to balance the addition of new ones, and the systems grow uncontrollably. Note that anchor lifetime reaches a plateau at high values of the ratio (Fig 6A). This is an artifact due to the finite number of iterations in the simulation, which bounds the lifetime of an anchor that has not been removed at the end of the simulation. Isotropically growing systems observed at a high ratio all reach their limit area after a similar number of iterations, which in turn truncates the mean lifetime at similar values. The plateau is a censored lower bound for anchors that are never removed in the isotropically growing phenotype, while lifetimes below the plateau are genuine, turnover-limited measurements.

**Fig 6:**
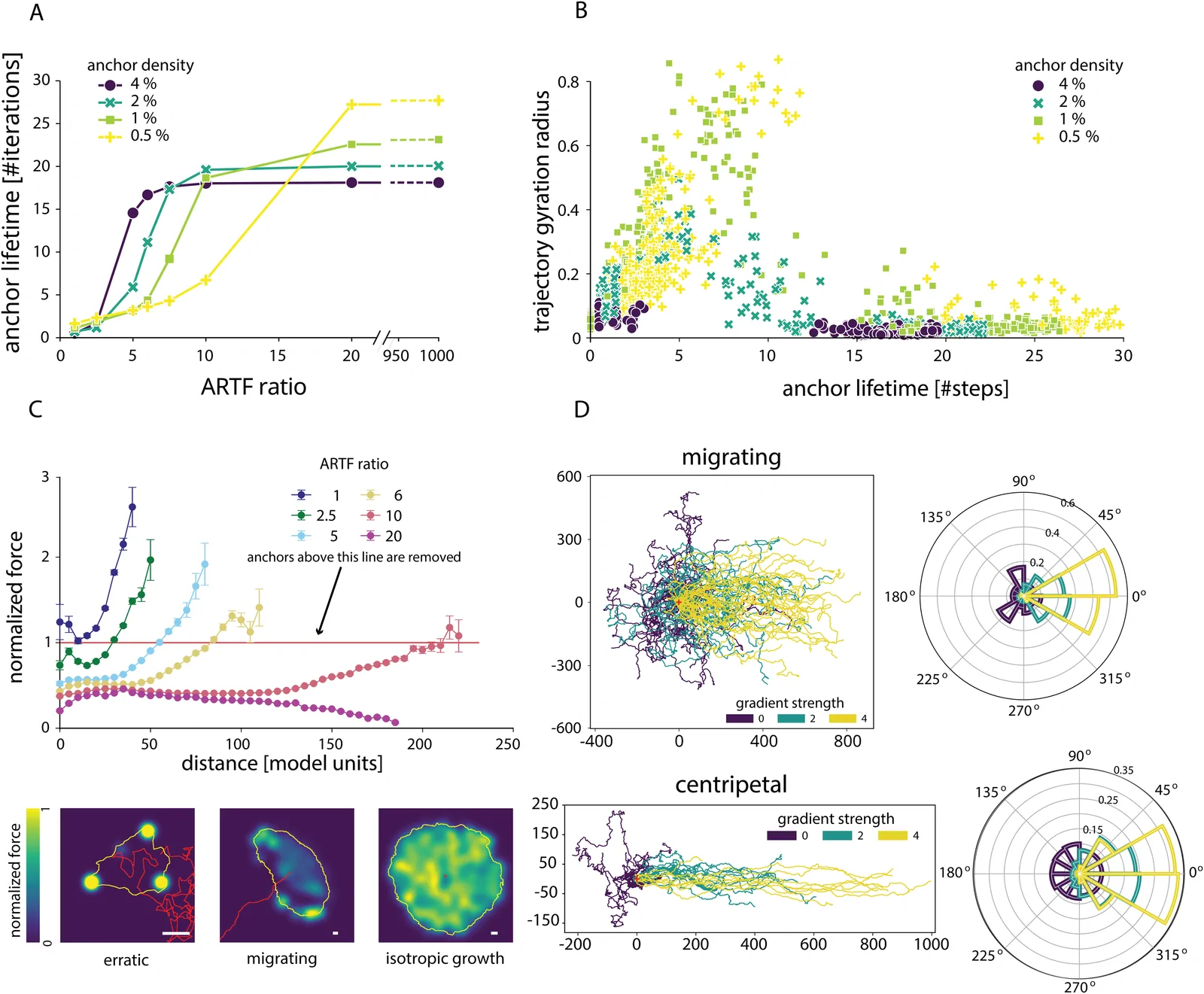
Intermediate anchor lifetime is optimal for migration. A) Plot of anchor lifetime as a function of the ARTF ratio for different values of the anchor density. Every point is an average over 10 simulations. B) Scatter plot of trajectory gyration radius as a function of the anchor lifetime for different values of the anchor density. Each point is a single simulation. C) (top panel) Force-distance relationship for different values of the ARTF ratio averaged over 10 simulations. Force is normalized by the anchor removal threshold. Distances are discretized in bins of 5 model length units from the system centroid (defined as the centroid of the system outline). Red horizontal line at 1 represents the anchor removal threshold. Everything above this line will be removed. The parameters for the simulations are: anchor density $\rho_{a}=1$, dimensionless dipole force magnitude *M* = 4, anchor removal threshold $T_{a}\in \{4,10,20,24,40,80\}$. (Bottom panel) spatial force distribution for typical realization of erratic (left), migrating (center), and isotropic growth (right) phenotypes. The corresponding cell trajectories and outlines are shown in red and yellow. Amplitude of the normalized force is color-coded from dark blue (low forces) to yellow (high forces). The parameters are: (erratic) anchor density $\rho_{a}=1$, dimensionless dipole force magnitude *M* = 16, anchor removal threshold $T_{a}=16$, (migrating) anchor density $\rho_{a}=0.5$, dimensionless dipole force magnitude *M* = 2, anchor removal threshold $T_{a}=19$, (isotropic growth) anchor density $\rho_{a}=1$, dimensionless dipole force magnitude *M* = 8, anchor removal threshold $T_{a}=80$. D) Effects of ARTF ratio gradient strengths for cells with migrating (top) and centripetal (bottom) cell phenotypes. Trajectories are displayed for different gradient values, see methods, (left panels) together with their trajectories’ end-to-end vectors angular distributions (right panels). The parameters for the simulations are: (top) anchor density $\rho_{a}=1$, dimensionless dipole force magnitude *M* = 4, anchor removal threshold $T_{a}=20$, (bottom) anchor density $\rho_{a}=2$, dimensionless dipole force magnitude *M* = 3, anchor removal threshold $T_{a}=7.5$.

Anchor lifetime, in turn, is a determining factor for the motion of the system. We characterized the extent of motion of the system by the gyration radius of the trajectory (see Materials and methods). Fig 6B shows that the gyration radius peaks at an intermediate anchor lifetime of around 5 simulation steps. Here, the systems are observed to move in a relatively persistent fashion. Shorter lifetimes mean that the anchors are removed too frequently for the system to develop any memory and polarize. This results in a smaller gyration radius and corresponds to the erratically moving systems and systems with high centripetal flow. Above a lifetime of 5, the gyration radius decreases again. Anchors live too long and become a hindrance to motion until the systems do not move at all (anisotropically and isotropically growing systems).

### In polarized systems, forces increase with the distance from the center and exceed the anchor removal threshold only at the periphery

In our recent work [39], we showed that local stress patterns exerted by rapidly moving fish epidermal keratocytes correlate with distance from the cell center and that edge retraction initiates preferentially at the longest distances, leading eventually to emergence and maintenance of a characteristic cell shape with long axis perpendicular to the direction of motion [38]. Using a simple mechanical model of the actin-myosin network without turnover, we showed that the increase of force with distance from the center is intrinsic to such networks. Here, we test whether a system with turnover exhibits a similar feature and how it depends on anchor turnover parameters (Fig 6C, S6 Movie and S8 Movie). Systems that build forces above the anchor removal threshold display increased force with distance from the center. When the system cannot build enough force to remove the anchors, this pattern is lost, and force instead decreases with distance as in an isotropically growing phenotype (Fig 6C, ratio 20, S7 Movie). Force-distance curves for polarized systems show forces large enough to remove anchors only at the system periphery (Fig 6C, ratio 5 to 10, migrating and anisotropically growing systems, S8 Movie), consistent with our findings in fish keratocytes [39]. In contrast, erratic and centripetal systems (Fig 6C, ratio 1 and 2.5, S6 Movie) generate forces above the anchor removal threshold everywhere (Fig 6C, ARTF ratio 1). Anchor turnover at each step prevents these systems from building polarity.

### External cues can bias migration

As shown above, the system of bonds, dipoles, and anchors is able to polarize and move spontaneously. We next tested if an external directional cue could bias this system. To simulate a cellular reaction to external cues, we introduced a directional gradient in the ARTF ratio. Such a gradient could arise in response to a variety of external adhesion-related cues. Rather than explicitly modeling specific external cues, here we simulate a universal response of the cell that reacts by strengthening of its adhesions to stimuli such as an increased density of external ligand (as in haptotaxis) or increased substrate rigidity (as in durotaxis).

Addition of an ARTF ratio gradient clearly biases the direction of migration of polarized systems towards higher ARTF ratio in a manner dependent on the strength of the gradient (Fig 6D migrating and S9 Movie), and also induces directional motion in the centripetal system, which does not migrate in the absence of a gradient, with the magnitude of this response likewise increasing with gradient strength (Fig 6D centripetal and S10 Movie).

## Discussion

Here, we found that a minimalistic model system consisting only of elastic bonds, force dipoles, and anchors, subjected to very simple turnover rules, is capable of self-organized polarization and directional motion. The key feature of this model is the detachment of anchors upon the buildup of force. The parameters of the model that define the rate of anchor detachment are the ratio between the anchor removal threshold and the dipole force (ARTF), and the anchor density. The emergent timescale of anchor detachment effectively defines the behavior of the system. If anchors detach at every simulation step, the system cannot build up any form of memory, fails to self-organize, and moves erratically, while if anchors are effectively permanent, the system expands isotropically. Critically, only when anchors detach at an intermediate rate can the system build up asymmetry, spontaneously polarize and undergo directed motion.

This result suggests that the ability to self-polarize is an intrinsic property of such an active mechanical network. Importantly, symmetry breaking results from mutual mechanics of contraction and adhesion. Our model features the addition of new network, mimicking actin protrusion, but the addition is uniform around the perimeter of the system and thus does not contribute to symmetry breaking. The system polarizes despite uniform protrusion. Likewise, our model does not feature any feedback from the outer boundary, which could represent membrane tension, and therefore such feedback is not necessary for symmetry breaking. Neither does the model feature any mass conservation constraints, thus excluding any mechanisms that depend on a limited supply of components. Mechanisms based on a limited pool of components could in principle facilitate polarization and broaden the parameter range where symmetry breaking is observed. For example, if adhesion supply is limited, the network could build more force per anchor and break anchors even with a very high removal threshold. We performed simulations limiting the anchor pool to 50 per cell and indeed observed symmetry breaking and motion for the range of parameters that produced unlimited growth in the case when anchors were not limited (Fig J in S1 Appendix). While these additional mechanisms (localized protrusion, membrane tension, limited pool of components) could be a part of the polarization process in living cells, our study suggests that the self-organization of contraction and adhesion alone is sufficient to break symmetry. This could be a key triggering event, especially in cases where polarization starts by local retraction at the prospective rear [17,18]. Asymmetry of protrusion may develop subsequently due to a limited actin pool or other mechanisms. Additional features may also result from adhesion dynamics that in reality are richer than represented in our model, such as reinforcement under applied force and viscous stick-slip behavior [36,61,62].

The switch from isotropic behavior to polarization happens in our system through tuning the balance between contractility and adhesion. Previous experimental studies indicated that balance between contractility and adhesion influences the speed of cell migration [63,64]. Importantly, our work shows that optimal balance between contractility and adhesion promotes the development of polarity itself. This suggests that any factor influencing contractility or adhesion may also promote or suppress symmetry breaking. This finding provides a framework for understanding the roles of various regulatory factors and molecules in cell polarization and suggests a possible intersection between chemical signaling networks and cytoskeletal machinery. According to our model, a trigger for polarization does not have to be a chemical gradient or other directional signal. Instead, a global change in the chemistry of contractile or adhesive machinery may trigger symmetry breaking. A similar finding was recently reported for a different system - a biomimetic model based on actin assembly [65]. At the same time, our simulation of the imposed spatial gradient of the adhesion strength demonstrated that the machinery is sensitive to directional signals, which can both promote polarization and influence the direction of motion in a system that is already polarized. An additional implication of these findings is that the signal-induced symmetry breaking and the cue providing directional information could be separate and independent signals.

The polarization mechanism in our model is consistent with the geometrical idea of distance-dependent protrusion-retraction switch and with the experimentally observed distance-dependence of forces [38,39]. Forces in the system build up at the most distant anchor points, eventually leading to their detachment. This limits lateral expansion of the system and results in a geometry reminiscent of fish keratocytes with a long axis normal to the direction of motion. This polarization process is also consistent with the idea of feedback between motion and polarity, such as in the UCSP model [28]: as the system moves, force dipoles accumulate at the back leading to the force build-up and detachment of adhesions, which promotes continued motion in the same direction, while adhesions at the front remain through multiple simulation cycles. A similar rear-contraction route to motility was featured in continuum active-gel analyses, in which a contraction-driven instability produces a bifurcation from a static to a steadily moving state [58,59,66]. We note the complementarity with those works that established the bifurcation analytically in a one-dimensional visco-active gel framework, whereas our discrete model shows that the same qualitative transition emerges from explicit, local adhesion turnover and additionally reproduces the characteristic shape of two-dimensional motile cells. Furthermore, the spatial and temporal scales of the model are quantitatively consistent with those of real cells. Because force dipoles act between neighboring network nodes, the distance between nodes (2 model units) can be mapped to the characteristic length of a myosin bipolar minifilament, approximately 300 nm [41]. Considering that the size of moving systems in our simulations is in the range of tens of thousands of square model units, we arrive at system linear dimensions in the range of tens of micrometers, comparable to those of migrating cells. The protrusion rate in the model is fixed at 4 model units per simulation step (~600 nm). Given typical cell protrusion rates of several tens to a few hundred nanometers per second [50,51], this implies that a simulation iteration is on the order of seconds. Accordingly, the full simulation duration (400 steps) corresponds to tens of minutes (typically ~40min\) for a protrusion rate of $100\;nm\cdot s^{-1}\backslash )$), consistent with the timescale required for cell polarization and migration over distances of several cell lengths. Thus, our model provides realistic relations between the microscopic processes and system-level behavior in both space and time. Importantly, realistic system dimensions are not set but emerge spontaneously in the model through self-organization.

## Conclusion

By dissecting polarization mechanisms via physical modeling, our study identifies a network of elastic, contractile, and adhesive elements as an independent module capable of symmetry breaking. Experimental biomimetic studies reconstituting active systems from purified components provide another important approach to dissecting polarization mechanisms. These studies mainly focused on reconstituting actin networks at the surface of small movable objects or inside lipid vesicles [65,67–70]. Our work demonstrates that feedback from the external boundary is not a necessary part of the polarization mechanism and suggests that symmetry breaking could be achieved in patches of actin-myosin network by tuning their attachment to a flat surface. This could inspire new biomimetic studies.

In living cells, symmetry breaking through contraction-adhesion mechanics is probably but one of multiple polarization mechanisms. Even this mechanism is likely much more complicated than represented in our model due to phenomena such as adhesion reinforcement, viscous dissipation of forces, etc. Nevertheless, by identifying a simple relationship between adhesion lifetime and polarization, our study provides a framework for understanding the integration of different mechanisms in cell polarization and a benchmark for further experimental and theoretical studies.

## Supporting information

S1 Appendix Supplementary Figs A–K. (PDF)

S1 MovieErratic system timelapse.Timelapse of a typical simulation of the erratic phenotype. Bonds are shown as black segments, dipoles as blue segments, and anchors as orange stars. Trajectory of the system centroid from the start of the simulation to the current position is shown in red. Grid size 50×50. Inset shows the outline of the system in black and the trace of the centroid in red. Reference for the simulation 1711312861 (same as Fig 3A-3F). Length of the simulation: 400 steps. The parameters for the simulation are: anchor density $\rho_{a}=1$, dimensionless dipole force magnitude *M* = 16, anchor removal threshold $T_{a}=16$.(AVI)

S2 MovieCentripetal system timelapse.Timelapse of a typical simulation of the centripetal phenotype. Bonds are shown as black segments, dipoles as blue segments, and anchors as orange stars. Trajectory of the system centroid from the start of the simulation to the current position is shown in red. Grid size 50×50. Inset shows the outline of the system in black and the trace of the centroid in red. Reference for the simulation 1708960333 (same as Fig 3G-3L). Length of the simulation: 400 steps. The parameters for the simulation are: anchor density $\rho_{a}=2$, dimensionless dipole force magnitude *M* = 3, anchor removal threshold $T_{a}=7.5$.(AVI)

S3 MovieIsotropic growing system timelapse.Timelapse of a typical simulation of the isotropic growing phenotype. Bonds are shown as black segments, dipoles as blue segments, and anchors as orange stars. Trajectory of the system centroid from the start of the simulation to the current position is shown in red. Grid size 50×50. Inset shows the outline of the system in black and the trace of the centroid in red. Reference for the simulation 1707691522 (same as Fig 4A-4F). Length of the simulation: 400 steps. The parameters for the simulation are: anchor density $\rho_{a}=1$, dimensionless dipole force magnitude *M* = 8, anchor removal threshold $T_{a}=80$.(AVI)

S4 MovieAnisotropic growing system timelapse.Timelapse of a typical simulation of the anisotropic growing phenotype. Bonds are shown as black segments, dipoles as blue segments, and anchors as orange stars. Trajectory of the system centroid from the start of the simulation to the current position is shown in red. Grid size 50×50. Inset shows the outline of the system in black and the trace of the centroid in red. Reference for the simulation 1711535798 (same as Fig 4G-4L). Length of the simulation: 400 steps. The parameters for the simulation are: anchor density $\rho_{a}=2$, dimensionless dipole force magnitude *M* = 4, anchor removal threshold $T_{a}=20$.(AVI)

S5 MovieMigrating system timelapse.Timelapse of a typical simulation of the migrating phenotype. Bonds are shown as black segments, dipoles as blue segments, and anchors as orange stars. Trajectory of the system centroid from the start of the simulation to the current position is shown in red. Grid size 50×50. Inset shows the outline of the system in black and the trace of the centroid in red. Reference for the simulation 1710023558 (same as Fig 5). Length of the simulation: 400 steps. The parameters for the simulation are: anchor density $\rho_{a}=1$, dimensionless dipole force magnitude *M* = 8, anchor removal threshold $T_{a}=60$.(AVI)

S6 MovieForce map timelapse of erratic system.Timelapse of force maps generated by a typical simulation of the erratic phenotype. System outline is shown in yellow. Trajectory of the system centroid from the start of the simulation to the current position is shown in red. Magnitude of the force on nearby anchors is shown color-coded. The force is normalized at each frame by the maximum force on an anchor in the frame. Frame number is shown in the left-top corner. Reference for the simulation 1711312861 (same as Fig 6C (erratic)). Length of the simulation: 400 steps. The parameters for the simulation are: anchor density $\rho_{a}=1$, dimensionless dipole force magnitude *M* = 16, anchor removal threshold $T_{a}=16$. See methods for a description of the generation of the force maps.(AVI)

S7 MovieForce map timelapse of migrating system.Timelapse of force maps generated by a typical simulation of the migrating phenotype. System outline is shown in yellow. Trajectory of the system centroid from the start of the simulation to the current position is shown in red. Magnitude of the force on nearby anchors is shown color-coded. The force is normalized at each frame by the maximum force on an anchor in the frame. Frame number is shown in the left-top corner. Reference for the simulation 1723538402 (same as Fig 6C (migrating)). Length of the simulation: 400 steps. The parameters for the simulation are: anchor density $\rho_{a}=0.5$, dimensionless dipole force magnitude *M* = 2, anchor removal threshold $T_{a}=19$. See methods for a description of the generation of the force maps.(AVI)

S8 MovieForce map timelapse of isotropic growing system.Timelapse of force maps generated by a typical simulation of the isotropic growing phenotype. System outline is shown in yellow. Trajectory of the system centroid from the start of the simulation to the current position is shown in red. Magnitude of the force on nearby anchors is shown color-coded. The force is normalized at each frame by the maximum force on an anchor in the frame. Frame number is shown in the left-top corner. Reference for the simulation 1707691522 (same as Fig 6C (isotropic growth)). Length of the simulation: 400 steps. The parameters for the simulation are: anchor density $\rho_{a}=1$, dimensionless dipole force magnitude *M* = 8, anchor removal threshold $T_{a}=80$. See methods for a description of the generation of the force maps.(AVI)

S9 MovieTrajectory comparison with and without directional cue for migrating systems.Comparison of trajectories of systems with different level of external cue. (Top) no cue, (middle) gradient 2, (bottom) gradient 4. Outline and trajectory of each system is shown in black and red respectively. All simulations were generated from the same set of parameters that corresponds to the migrating phenotype. The parameters for the simulation are: anchor density $\rho_{a}=1$, dimensionless dipole force magnitude *M* = 4, anchor removal threshold $T_{a}=20$.(AVI)

S10 MovieTimelapse of a centripetal system with directional cue.Timelapse of a system from the centripetal phenotype with an external cue. Bonds are shown as black segments, dipoles as blue segments, and anchors as orange stars. Trajectory of the system centroid from the start of the simulation to the current position is shown in red. Grid size 50×50. Inset shows the outline of the system in black and the trace of the centroid in red. Reference for the simulation 1743622318. Length of the simulation: 400 steps. The parameters for the simulation are: anchor density $\rho_{a}=2$, dimensionless dipole force magnitude *M* = 3, anchor removal threshold $T_{a}=7.5$.(AVI)

## References

[1] Lauffenburger DA, Horwitz AF. Cell migration: a physically integrated molecular process. Cell. 1996;84(3):359–69. doi: 10.1016/s0092-8674(00)81280-5 8608589

[2] Doubrovinski K, Kruse K. Cell motility resulting from spontaneous polymerization waves. Phys Rev Lett. 2011;107(25):258103. doi: 10.1103/PhysRevLett.107.258103 22243118

[3] Verkhovsky AB. The mechanisms of spatial and temporal patterning of cell-edge dynamics. Curr Opin Cell Biol. 2015;36:113–21. doi: 10.1016/j.ceb.2015.09.001 26432504

[4] Stankevicins L, Ecker N, Terriac E, Maiuri P, Schoppmeyer R, Vargas P, et al. Deterministic actin waves as generators of cell polarization cues. Proc Natl Acad Sci U S A. 2020;117(2):826–35. doi: 10.1073/pnas.1907845117 31882452 PMC6969493

[5] Seetharaman S, Etienne-Manneville S. Cytoskeletal crosstalk in cell migration. Trends Cell Biol. 2020;30(9):720–35. doi: 10.1016/j.tcb.2020.06.004 32674938

[6] Ierushalmi N, Keren K. Cytoskeletal symmetry breaking in animal cells. Curr Opin Cell Biol. 2021;72:91–9. doi: 10.1016/j.ceb.2021.07.003 34375786

[7] Banavar SP, Trogdon M, Drawert B, Yi T-M, Petzold LR, Campàs O. Coordinating cell polarization and morphogenesis through mechanical feedback. PLoS Comput Biol. 2021;17(1):e1007971. doi: 10.1371/journal.pcbi.1007971 33507956 PMC7872284

[8] Asnacios A, Hamant O. The mechanics behind cell polarity. Trends Cell Biol. 2012;22(11):584–91. doi: 10.1016/j.tcb.2012.08.005 22980034

[9] Drubin DG, Nelson WJ. Origins of cell polarity. Cell. 1996;84(3):335–44. doi: 10.1016/s0092-8674(00)81278-7 8608587

[10] Fouchard J, Mitrossilis D, Asnacios A. Acto-myosin based response to stiffness and rigidity sensing. Cell Adh Migr. 2011;5(1):16–9. doi: 10.4161/cam.5.1.13281 20818154 PMC3038090

[11] Graziano BR, Weiner OD. Self-organization of protrusions and polarity during eukaryotic chemotaxis. Curr Opin Cell Biol. 2014;30:60–7. doi: 10.1016/j.ceb.2014.06.007 24998184 PMC4177965

[12] Charras G, Sahai E. Physical influences of the extracellular environment on cell migration. Nat Rev Mol Cell Biol. 2014;15(12):813–24. doi: 10.1038/nrm3897 25355506

[13] Chang F, Minc N. Electrochemical control of cell and tissue polarity. Annu Rev Cell Dev Biol. 2014;30:317–36. doi: 10.1146/annurev-cellbio-100913-013357 25062359

[14] Allen GM, Mogilner A, Theriot JA. Electrophoresis of cellular membrane components creates the directional cue guiding keratocyte galvanotaxis. Curr Biol. 2013;23(7):560–8. doi: 10.1016/j.cub.2013.02.047 23541731 PMC3718648

[15] Allen GM, Lee KC, Barnhart EL, Tsuchida MA, Wilson CA, Gutierrez E, et al. Cell mechanics at the rear act to steer the direction of cell migration. Cell Syst. 2020;11(3):286-299.e4. doi: 10.1016/j.cels.2020.08.008 32916096 PMC7530145

[16] Verkhovsky AB, Svitkina TM, Borisy GG. Self-polarization and directional motility of cytoplasm. Curr Biol. 1999;9(1):11–20. doi: 10.1016/s0960-9822(99)80042-6 9889119

[17] Yam PT, Wilson CA, Ji L, Hebert B, Barnhart EL, Dye NA, et al. Actin-myosin network reorganization breaks symmetry at the cell rear to spontaneously initiate polarized cell motility. J Cell Biol. 2007;178(7):1207–21. doi: 10.1083/jcb.200706012 17893245 PMC2064654

[18] Cramer LP. Forming the cell rear first: breaking cell symmetry to trigger directed cell migration. Nat Cell Biol. 2010;12(7):628–32. doi: 10.1038/ncb0710-628 20596043

[19] Cramer LP, Kay RR, Zatulovskiy E. Repellent and attractant guidance cues initiate cell migration by distinct rear-driven and front-driven cytoskeletal mechanisms. Curr Biol. 2018;28(6):995-1004.e3. doi: 10.1016/j.cub.2018.02.024 29526589 PMC5863766

[20] Edelstein-Keshet L, Holmes WR, Zajac M, Dutot M. From simple to detailed models for cell polarization. Philos Trans R Soc Lond B Biol Sci. 2013;368(1629):20130003. doi: 10.1098/rstb.2013.0003 24062577 PMC3785957

[21] Mogilner A, Barnhart EL, Keren K. Experiment, theory, and the keratocyte: an ode to a simple model for cell motility. Semin Cell Dev Biol. 2020;100:143–51. doi: 10.1016/j.semcdb.2019.10.019 31718950

[22] Marée AFM, Jilkine A, Dawes A, Grieneisen VA, Edelstein-Keshet L. Polarization and movement of keratocytes: a multiscale modelling approach. Bull Math Biol. 2006;68(5):1169–211. doi: 10.1007/s11538-006-9131-7 16794915

[23] Shi C, Huang C-H, Devreotes PN, Iglesias PA. Interaction of motility, directional sensing, and polarity modules recreates the behaviors of chemotaxing cells. PLoS Comput Biol. 2013;9(7):e1003122. doi: 10.1371/journal.pcbi.1003122 23861660 PMC3701696

[24] Copos C, Mogilner A. A hybrid stochastic-deterministic mechanochemical model of cell polarization. Mol Biol Cell. 2020;31(15):1637–49. doi: 10.1091/mbc.E19-09-0549 32459563 PMC7521800

[25] Rappel W-J, Edelstein-Keshet L. Mechanisms of cell polarization. Curr Opin Syst Biol. 2017;3:43–53. doi: 10.1016/j.coisb.2017.03.005 29038793 PMC5640326

[26] Houk AR, Jilkine A, Mejean CO, Boltyanskiy R, Dufresne ER, Angenent SB, et al. Membrane tension maintains cell polarity by confining signals to the leading edge during neutrophil migration. Cell. 2012;148(1–2):175–88. doi: 10.1016/j.cell.2011.10.050 22265410 PMC3308728

[27] Sadhu RK, Iglič A, Gov NS. A minimal cell model for lamellipodia-based cellular dynamics and migration. J Cell Sci. 2023;136(14):jcs260744. doi: 10.1242/jcs.260744 37497740

[28] Maiuri P, Rupprecht J-F, Wieser S, Ruprecht V, Bénichou O, Carpi N, et al. Actin flows mediate a universal coupling between cell speed and cell persistence. Cell. 2015;161(2):374–86. doi: 10.1016/j.cell.2015.01.056 25799384

[29] Ziebert F, Aranson IS. Effects of adhesion dynamics and substrate compliance on the shape and motility of crawling cells. PLoS One. 2013;8(5):e64511. doi: 10.1371/journal.pone.0064511 23741334 PMC3669322

[30] Nedelec F, Foethke D. Collective Langevin dynamics of flexible cytoskeletal fibers. New J Phys. 2007;9(11):427–427. doi: 10.1088/1367-2630/9/11/427

[31] Kim T, Hwang W, Lee H, Kamm RD. Computational analysis of viscoelastic properties of crosslinked actin networks. PLoS Comput Biol. 2009;5(7):e1000439. doi: 10.1371/journal.pcbi.1000439 19609348 PMC2703781

[32] Maxian O, Peláez RP, Mogilner A, Donev A. Simulations of dynamically cross-linked actin networks: morphology, rheology, and hydrodynamic interactions. PLoS Comput Biol. 2021;17(12):e1009240. doi: 10.1371/journal.pcbi.1009240 34871298 PMC8675935

[33] Rutkowski DM, Vavylonis D. Discrete mechanical model of lamellipodial actin network implements molecular clutch mechanism and generates arcs and microspikes. PLoS Comput Biol. 2021;17(10):e1009506. doi: 10.1371/journal.pcbi.1009506 34662335 PMC8553091

[34] Ronceray P, Broedersz CP, Lenz M. Fiber networks amplify active stress. Proc Natl Acad Sci U S A. 2016;113(11):2827–32. doi: 10.1073/pnas.1514208113 26921325 PMC4801261

[35] Barnhart E, Lee K-C, Allen GM, Theriot JA, Mogilner A. Balance between cell-substrate adhesion and myosin contraction determines the frequency of motility initiation in fish keratocytes. Proc Natl Acad Sci U S A. 2015;112(16):5045–50. doi: 10.1073/pnas.1417257112 25848042 PMC4413303

[36] Sens P. Stick-slip model for actin-driven cell protrusions, cell polarization, and crawling. Proc Natl Acad Sci U S A. 2020;117(40):24670–8. doi: 10.1073/pnas.2011785117 32958682 PMC7547270

[37] Chen Y, Saintillan D, Rangamani P. Interplay between mechanosensitive adhesions and membrane tension regulates cell motility. PRX Life. 2023;1(2). doi: 10.1103/prxlife.1.023007

[38] Raynaud F, Ambühl ME, Gabella C, Bornert A, Sbalzarini IF, Meister J-J, et al. Minimal model for spontaneous cell polarization and edge activity in oscillating, rotating and migrating cells. Nature Phys. 2016;12(4):367–73. doi: 10.1038/nphys3615

[39] Messi Z, Bornert A, Raynaud F, Verkhovsky AB. Traction forces control cell-edge dynamics and mediate distance sensitivity during cell polarization. Curr Biol. 2020;30(9):1762-1769.e5. doi: 10.1016/j.cub.2020.02.078 32220324

[40] Broedersz CP, MacKintosh FC. Modeling semiflexible polymer networks. Rev Mod Phys. 2014;86:995–1036. doi: 10.1103/RevModPhys.86.995

[41] Hu S, Dasbiswas K, Guo Z, Tee Y-H, Thiagarajan V, Hersen P, et al. Long-range self-organization of cytoskeletal myosin II filament stacks. Nat Cell Biol. 2017;19(2):133–41. doi: 10.1038/ncb3466 28114270

[42] Gittes F, Mickey B, Nettleton J, Howard J. Flexural rigidity of microtubules and actin filaments measured from thermal fluctuations in shape. J Cell Biol. 1993;120(4):923–34. doi: 10.1083/jcb.120.4.923 8432732 PMC2200075

[43] Berro J, Michelot A, Blanchoin L, Kovar DR, Martiel J-L. Attachment conditions control actin filament buckling and the production of forces. Biophys J. 2007;92(7):2546–58. doi: 10.1529/biophysj.106.094672 17208983 PMC1864840

[44] Cameron LA, Svitkina TM, Vignjevic D, Theriot JA, Borisy GG. Dendritic organization of actin comet tails. Curr Biol. 2001;11(2):130–5. doi: 10.1016/s0960-9822(01)00022-7 11231131

[45] Arai Y, Yasuda R, Akashi K, Harada Y, Miyata H, Kinosita K Jr, et al. Tying a molecular knot with optical tweezers. Nature. 1999;399(6735):446–8. doi: 10.1038/20894 10365955

[46] Murrell MP, Gardel ML. F-actin buckling coordinates contractility and severing in a biomimetic actomyosin cortex. Proc Natl Acad Sci U S A. 2012;109(51):20820–5. doi: 10.1073/pnas.1214753109 23213249 PMC3529094

[47] Haviv L, Gillo D, Backouche F, Bernheim-Groswasser A. A cytoskeletal demolition worker: myosin II acts as an actin depolymerization agent. J Mol Biol. 2008;375(2):325–30. doi: 10.1016/j.jmb.2007.09.066 18021803

[48] Vogel SK, Petrasek Z, Heinemann F, Schwille P. Myosin motors fragment and compact membrane-bound actin filaments. Elife. 2013;2:e00116. doi: 10.7554/eLife.00116 23326639 PMC3545444

[49] Awrangjeb M. Using point cloud data to identify, trace, and regularize the outlines of buildings. Int J Remote Sensing. 2016;37(3):551–79. doi: 10.1080/01431161.2015.1131868

[50] Gabella C, Bertseva E, Bottier C, Piacentini N, Bornert A, Jeney S, et al. Contact angle at the leading edge controls cell protrusion rate. Curr Biol. 2014;24(10):1126–32. doi: 10.1016/j.cub.2014.03.050 24794299

[51] Carlier MF, Pantaloni D. Control of actin dynamics in cell motility. J Mol Biol. 1997;269(4):459–67. doi: 10.1006/jmbi.1997.1062 9217250

[52] Gough B. GNU scientific library reference manual. Network Theory; 2009.

[53] The CGAL Project. CGAL User and Reference Manual. 6.0.1 ed. CGAL Editorial Board; 2024. Available from: https://doc.cgal.org/6.0.1/Manual/packages.html

[54] Schindelin J, Arganda-Carreras I, Frise E, Kaynig V, Longair M, Pietzsch T, et al. Fiji: an open-source platform for biological-image analysis. Nat Methods. 2012;9(7):676–82. doi: 10.1038/nmeth.2019 22743772 PMC3855844

[55] Henson JH, Yeterian M, Weeks RM, Medrano AE, Brown BL, Geist HL, et al. Arp2/3 complex inhibition radically alters lamellipodial actin architecture, suspended cell shape, and the cell spreading process. Mol Biol Cell. 2015;26(5):887–900. doi: 10.1091/mbc.E14-07-1244 25568343 PMC4342025

[56] Jankowska KI, Williamson EK, Roy NH, Blumenthal D, Chandra V, Baumgart T, et al. Integrins modulate T cell receptor signaling by constraining actin flow at the immunological synapse. Front Immunol. 2018;9:25. doi: 10.3389/fimmu.2018.00025 29403502 PMC5778112

[57] Gardel ML, Sabass B, Ji L, Danuser G, Schwarz US, Waterman CM. Traction stress in focal adhesions correlates biphasically with actin retrograde flow speed. J Cell Biol. 2008;183(6):999–1005. doi: 10.1083/jcb.200810060 19075110 PMC2600750

[58] Recho P, Putelat T, Truskinovsky L. Contraction-driven cell motility. Phys Rev Lett. 2013;111(10):108102. doi: 10.1103/PhysRevLett.111.108102 25166712

[59] Recho P, Putelat T, Truskinovsky L. Mechanics of motility initiation and motility arrest in crawling cells. J Mech Phys Solids. 2015;84:469–505. doi: 10.1016/j.jmps.2015.08.006

[60] Ambühl ME, Brepsant C, Meister J-J, Verkhovsky AB, Sbalzarini IF. High-resolution cell outline segmentation and tracking from phase-contrast microscopy images. J Microsc. 2012;245(2):161–70. doi: 10.1111/j.1365-2818.2011.03558.x 21999192

[61] Bershadsky A, Kozlov M, Geiger B. Adhesion-mediated mechanosensitivity: a time to experiment, and a time to theorize. Curr Opin Cell Biol. 2006;18(5):472–81. doi: 10.1016/j.ceb.2006.08.012 16930976

[62] Hennig K, Wang I, Moreau P, Valon L, DeBeco S, Coppey M, et al. Stick-slip dynamics of cell adhesion triggers spontaneous symmetry breaking and directional migration of mesenchymal cells on one-dimensional lines. Sci Adv. 2020;6(1):eaau5670. doi: 10.1126/sciadv.aau5670 31921998 PMC6941913

[63] Palecek SP, Loftus JC, Ginsberg MH, Lauffenburger DA, Horwitz AF. Integrin-ligand binding properties govern cell migration speed through cell-substratum adhesiveness. Nature. 1997;385(6616):537–40. doi: 10.1038/385537a0 9020360

[64] Gupton SL, Waterman-Storer CM. Spatiotemporal feedback between actomyosin and focal-adhesion systems optimizes rapid cell migration. Cell. 2006;125(7):1361–74. doi: 10.1016/j.cell.2006.05.029 16814721

[65] Razavi S, Wong F, Abubaker-Sharif B, Matsubayashi HT, Nakamura H, Nguyen NTH, et al. Synthetic control of actin polymerization and symmetry breaking in active protocells. Sci Adv. 2024;10(24):eadk9731. doi: 10.1126/sciadv.adk9731 38865458 PMC11168455

[66] Wössner V, Drozdowski OM, Ziebert F, Schwarz US. Active gel model for one-dimensional cell migration coupling actin flow and adhesion dynamics. New J Phys. 2024;26(7):073039. doi: 10.1088/1367-2630/ad63c5

[67] Guevorkian K, Manzi J, Pontani L-L, Brochard-Wyart F, Sykes C. Mechanics of biomimetic liposomes encapsulating an actin shell. Biophys J. 2015;109(12):2471–9. doi: 10.1016/j.bpj.2015.10.050 26682806 PMC4701011

[68] Mulla Y, Aufderhorst-Roberts A, Koenderink GH. Shaping up synthetic cells. Phys Biol. 2018;15(4):041001. doi: 10.1088/1478-3975/aab923 29570090

[69] Sakamoto R, Banerjee DS, Yadav V, Chen S, Gardel ML, Sykes C, et al. Membrane tension induces F-actin reorganization and flow in a biomimetic model cortex. Commun Biol. 2023;6(1):325. doi: 10.1038/s42003-023-04684-7 36973388 PMC10043271

[70] Cáceres R, Abou-Ghali M, Plastino J. Reconstituting the actin cytoskeleton at or near surfaces in vitro. Biochim Biophys Acta. 2015;1853(11 Pt B):3006–14. doi: 10.1016/j.bbamcr.2015.07.021 26235437

