# Supplementary material for: Balanced contractility and adhesion drive polarization in a minimal elastic actomyosin network
Source: PLoS Comput Biol. 2026 Sep 18;22(9):e1014750. doi: 10.1371/journal.pcbi.1014750 (PMC13630322; doi:10.1371/journal.pcbi.1014750)
Supplement: Supplementary file 1 [file pcbi.1014750.s001.pdf]

Balanced contractility and adhesion drive polarization in a minimal elastic actomyosin network

S1 Appendix

Zeno Messi[*1,2], Franck Raynaud[3], Nathan W Goehring[1,4], and Alexander B Verkhovsky[5]

[1] *Francis Crick Institute, London, UK*
[2] *Laboratory of Mechanisms of Cell Polarization and Cell Fusion, University of Geneva, Switzerland*
[3] *Computer Science Department, University of Geneva, Switzerland*
[4] *Department of Biochemistry, University of Oxford, Oxford, UK*
[5] *Laboratory of Biological Electron Microscopy, EPFL, Route de la Sorge, Lausanne 1015, Switzerland*
[*] *zeno.messi@unige.ch*

List of Supplementary Texts

Text A Energy contributions . . . S2
Text B Non-dimensionalization . . . S3
Text C Biologically motivated parameter values . . . S3
Text D Numerical implementation of the dipole force . . . S4
Text E Complete list of parameters . . . S4
Text F Fraction of forces failing cancellation . . . S9
Text G Spatial analysis of adhesion failures . . . S13

List of Supplementary Figures

Fig A Explanatory sketches of the model . . . S5
Fig B Time evolution of the relative contributions of the different network disassembly mechanisms (dipoles and bonds) . . . S6
Fig C Time evolution of the relative contributions of the different network disassembly mechanisms (bonds, nodes and anchors) . . . S7
Fig D Mean Squared Displacement (MSD) of normalized trajectories . . . S8
Fig E Cumulative distribution of the fraction of forces failing cancellation for the different phenotypes . . . S9
Fig F Time evolution of the effective cell growth rate . . . S10
Fig G Time evolution of the number of elements in the networks . . . S11
Fig H Spatial analysis of anchor removal . . . S12
Fig I Spatial density profiles relative to the axis of motion . . . S15
Fig J A limited resource is not required for polarization . . . S16
Fig K Simulations varying the maximal number of dipoles between a pair of nodes and the interior angle for hinge removal . . . S17

List of Tables

Table A Complete list of model parameters . . . S4
Table B Statistics of the distribution for the different phenotypes . . . S9

Supplementary Methods: Model and parameter values

This section derives the energy terms of the Hamiltonian from the worm-like-chain (WLC) description of actin, presents the non-dimensionalization that defines the dimensionless model parameters, and gives the biologically motivated values assigned to them.

Text A. Energy contributions

The system energy has four contributions: stretching/compression of segments (E_{spring}), bending of bonds at the mid-bond hinge (E_{bend}), attractive dipoles (E_{dip}), and substrate deformation at the anchors (E_{sub}). Following [1, 2, 3] we obtain the first two from the WLC model, in which a filament is a chain of inextensible segments whose relative orientations fluctuate, characterized by a persistence length ℓ_p.

Bending. A hinge joins two segments of length ℓ_0, so the bent element has length $2\ell_0$. Assuming uniform bending to a radius of curvature R, its WLC bending energy is

$$E_{\text{bend}}(R) = \frac{k_B T\, \ell_p}{2} \frac{2\ell_0}{R^2} = \frac{k_B T\, \ell_p\, \ell_0}{R^2}. \tag{1}$$

A hinge deflected by ϕ from straight has radius of curvature $R = \ell_0/[2\sin(\phi/2)]$ (the circumradius of the two segments; see Fig A Panel B), so

$$E_{\text{bend}}(\phi) = \frac{k_B T\, \ell_p\, \ell_0}{(\ell_0/[2\sin(\phi/2)])^2} = \frac{2k_B T\, \ell_p}{\ell_0} \times 2\sin^2\left(\frac{\phi}{2}\right). \tag{2}$$

Consecutive segments that are initially aligned on the lattice also form a hinge and are penalized identically; segments that are not initially aligned may reorient freely. The energy to fully bend a hinge and the associated typical force are

$$E_0 = E_{\text{bend}}(\pi) = \frac{4k_B T\, \ell_p}{\ell_0}, \qquad F_0 = \frac{2k_B T\, \ell_p}{\ell_0^2}. \tag{3}$$

Stretching. In the linear regime the WLC stretching energy of a segment of length ℓ and rest length ℓ_0 is [3]

$$E_{\text{spring}}(\ell) = \frac{90\, k_B T\, \ell_p^2}{\ell_0^4} \frac{(\ell-\ell_0)^2}{2} = \frac{2k_B T\, \ell_p}{\ell_0} \times \frac{45\, \ell_p}{\ell_0} \times \frac{1}{2}\left(\frac{\ell}{\ell_0} - 1\right)^2. \tag{4}$$

Dipoles. Non-muscle myosin II minifilaments are modeled as attractive dipoles exerting a constant force F between two nodes separated by a distance ℓ, giving

$$E_{\text{dip}} = F\,\ell = \frac{2k_B T\, \ell_p}{\ell_0} \times \frac{F}{F_0} \times \frac{\ell}{\ell_0}. \tag{5}$$

Substrate. Each anchor is attached to its initial position by a spring of zero rest length and spring constant K, so a displacement d from that position costs

$$E_{\text{sub}} = K\frac{d^2}{2} = \frac{2k_B T\, \ell_p}{\ell_0} \times \frac{K\ell_0^3}{2k_B T\, \ell_p} \times \frac{1}{2}\left(\frac{d}{\ell_0}\right)^2. \tag{6}$$

The force borne by an anchor, used in the force-dependent removal rule of the main text, is $F_{\text{anchor}} = K\,d = \mu_s\, d$ in model units.

Hamiltonian. Summing the four contributions:

$$\mathcal{H} = \frac{2k_BT\,\ell_p}{\ell_0}\Bigg[\sum_{\text{segments}\,(i)} \frac{45\,\ell_p}{\ell_0}\,\frac{1}{2}\left(\frac{\ell_i}{\ell_0}-1\right)^2 + \sum_{\text{hinges}\,(i)} 2\sin^2\left(\frac{\phi_i}{2}\right) + \sum_{\text{anchors}\,(i)} \frac{K\ell_0^3}{2k_BT\,\ell_p}\,\frac{1}{2}\left(\frac{d_i}{\ell_0}\right)^2 + \sum_{\text{dipoles}\,(i)} \frac{F}{F_0}\,\frac{\ell_i}{\ell_0}\Bigg]. \tag{7}$$

Text B. Non-dimensionalization

The Hamiltonian is made dimensionless by setting the bending prefactor and the segment rest length to unity:

$$\frac{2k_BT\,\ell_p}{\ell_0} = 1, \qquad \ell_0 = 1, \tag{8}$$

so that energies are measured in units of the bending prefactor and lengths in units of ℓ_0. We set the three dimensionless parameters:

$$\mu_b = \frac{45\,\ell_p}{\ell_0}, \qquad M = \frac{F}{F_0}, \qquad \mu_s = \frac{K\ell_0^3}{2k_BT\,\ell_p}, \tag{9}$$

which reduce Eq. 7 to the form used in the main text:

$$\mathcal{H} = \mu_b \sum_{\text{segments}\,(i)} \frac{1}{2}(\ell_i-1)^2 + \sum_{\text{hinges}\,(i)} 2\sin^2\left(\frac{\phi_i}{2}\right) + \mu_s \sum_{\text{anchors}\,(i)} \frac{d_i^2}{2} + M \sum_{\text{dipoles}\,(i)} \ell_i. \tag{10}$$

Text C. Biologically motivated parameter values

Length scales. The internode distance $2\ell_0$ is identified with the length of a myosin bipolar minifilament, 0.1–1 μm [4], and the actin persistence length is taken as $\ell_p \sim 10\,\mu$m [5, 6].

Bond spring constant. With these scales, Eq. 9 gives

$$\mu_b = \frac{45\,\ell_p}{\ell_0} \;\sim\; 450\text{–}4500. \tag{11}$$

We arbitrarily set an intermediate value $\mu_b = 1024$.

Dipole force. Substituting the same scales into Eq. 3 yields a typical bond-bending force

$$F_0 \sim 1\text{–}10 \text{ pN}, \tag{12}$$

consistent with experimental estimates [7, 8]. Because a network in which a single dipole cannot bend a bond simply grows without remodeling, we restrict the analysis to dipole forces capable of bending bonds and set the dimensionless dipole force magnitude in the range $M = 1$–16.

Substrate stiffness. To represent a rigid substrate we take a large substrate spring constant, $\mu_s = 10^6$.

Text D. Numerical implementation of the dipole force

For numerical stability the constant dipole force is regularized by a continuous short-range cut-off at a length ℓ_c. The force on a dipole vertex is

$$F_{\text{dip}} = \begin{cases} M, & \ell > \ell_c, \\ M\left(\dfrac{2\ell}{\ell_c} - 1\right), & \frac{\ell_c}{2} < \ell < \ell_c, \\ 0, & \ell < \frac{\ell_c}{2}, \end{cases} \tag{13}$$

with corresponding energy

$$E_{\text{dip}} = \begin{cases} M(\ell - \ell_c) + \dfrac{M\ell_c}{4}, & \ell > \ell_c, \\ \dfrac{M}{\ell_c}\ell^2 - M\ell + \dfrac{M\ell_c}{4}, & \frac{\ell_c}{2} < \ell < \ell_c, \\ 0, & \ell < \frac{\ell_c}{2}, \end{cases} \tag{14}$$

where ℓ is the dipole length and $\ell_c = 0.01$ the cut-off length.

Text E. Complete list of parameters

The parameters fall into structural (bond, anchor, dipole densities), energetic (bond spring constant, dipole force), and turnover/pruning groups (anchor removal threshold, protrusion rate, merging distance, hinge removal angle). Structural and energetic values were fixed from the properties of the actin–myosin network and previous work [1, 2]; we varied the parameters governing anchor turnover, the anchor density, the anchor removal threshold, and the dipole force magnitude, which control the phenotype. Varying the remaining parameters shifted the phenotype boundaries in parameter space but produced no additional phenotypes. Parameters are listed in Table A.

Table A: Complete list of model parameters, with the values or ranges, and the references used for the conversion from model to physical units.

Name	Description	Value / range	Physical value & References
ρ	lattice (bond) retention probability	0.7	[1, 2]
ρ_a	anchor density	0.05–4%	[2]
ρ_d	dipole density	0.3	[2]
	max bonds per pair of nodes	10	This study
	max dipoles per pair of nodes	10	This study
ℓ_0	segment rest length	1	$\approx$ 150 nm [4]
F_0	dimensionless force to fully bend a hinge	1	1–10 pN, [6, 7]
μ_b	dimensionless bond spring constant	1024	100 pN.nm^{-1} [3]
μ_s	dimensionless substrate spring constant	10^6	10^5 pN.nm^{-1}
M	dimensionless dipole force magnitude	1–16	1–100 pN, [6, 7]
T_a	anchor removal threshold	0–16000	$\approx 10^5$ pN, [6, 7]
P	protrusion rate	4	$\approx 100\ \text{nm}\,\text{s}^{-1}$ [9, 10]
d_{merge}	node-merging distance	0.02	$\sim$ 3 nm
α_{rem}	interior angle for hinge removal	$\cos^{-1}(0.8) \approx 37°$	[11, 12, 13]
ℓ_c	dipole force cut-off length	0.01	$\sim$ 1.5 nm
D	removal-threshold gradient strength	0–4	This study

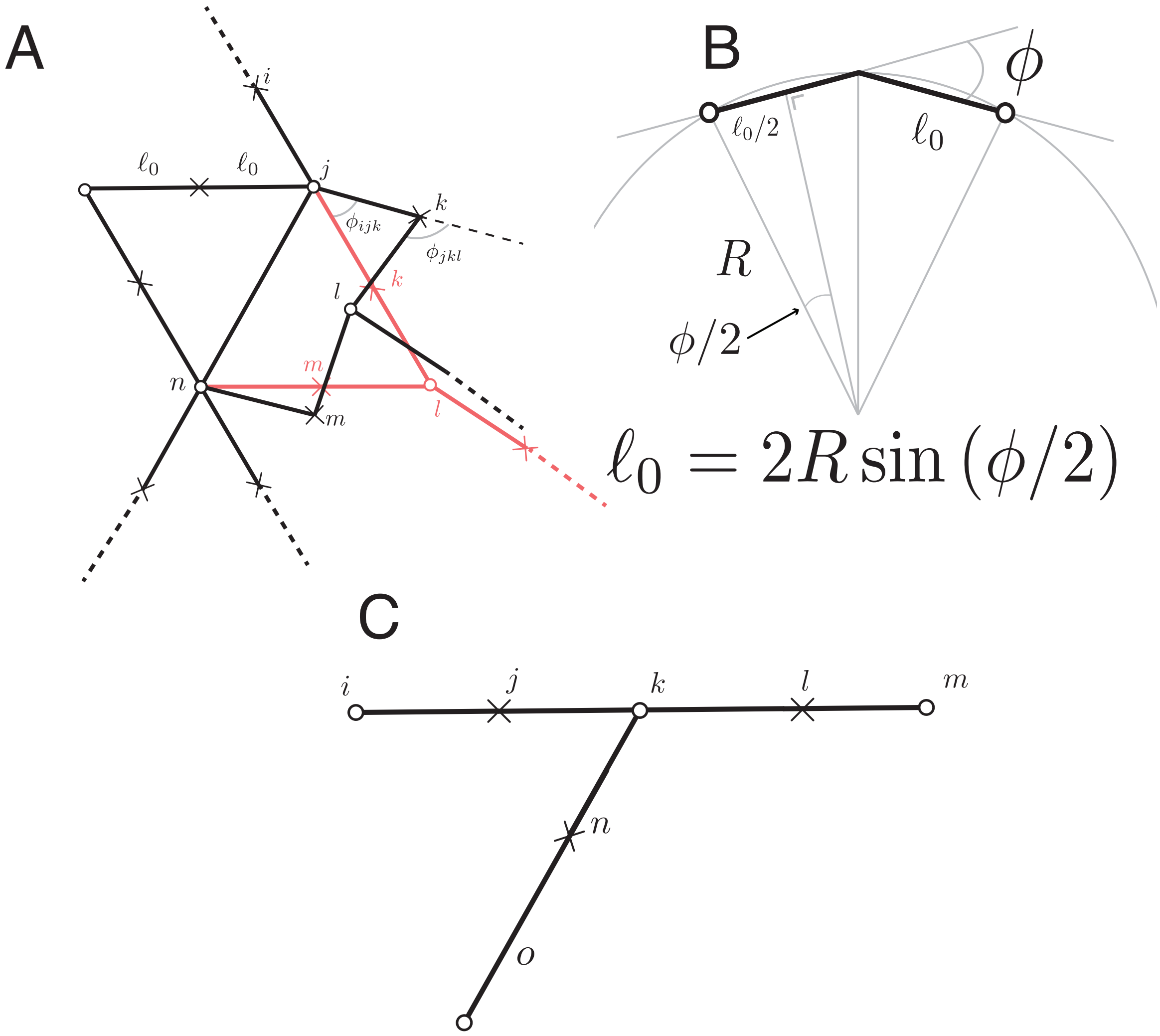

Fig A: Explanatory sketches of the model. Lattice nodes are marked by open circles. Midpoints of bonds are marked by crosses. A. Detail of the network and example of bending. (i, j), (j, k), (k, l), (l, m), (m, n) are segments. (j, k, l) and (l, m, n) are bonds. j, l, n are lattice nodes. Initial network configuration is shown in red. An example of a perturbed configuration of the network is shown in black. ϕ_{ijk} is the bending angle between segments (i, j) and (j, k). ℓ_0 is the length of a segment. B. Geometric explanation of the relationship between curvature radius and bending angle. A bond of length $2\ell_0$ is bent in its middle. R is the curvature radius of the bend, i.e. the circumradius of the triangle defined by the two ends of the bonds and its midpoint. Construction lines are shown in light gray. C. Explanation of what a hinge is. The sketch represents a detail of the network in its initial configuration. (i, j, k), (k, l, m) and (k, n, o) are hinges because they are bonds that can bend in their middle. (j, k, l) is a hinge because the segments (j, k) and (k, l) are aligned. (j, k, n) is not a hinge because it is neither a bond, nor are (j, k) and (k, n) aligned in the initial network configuration.

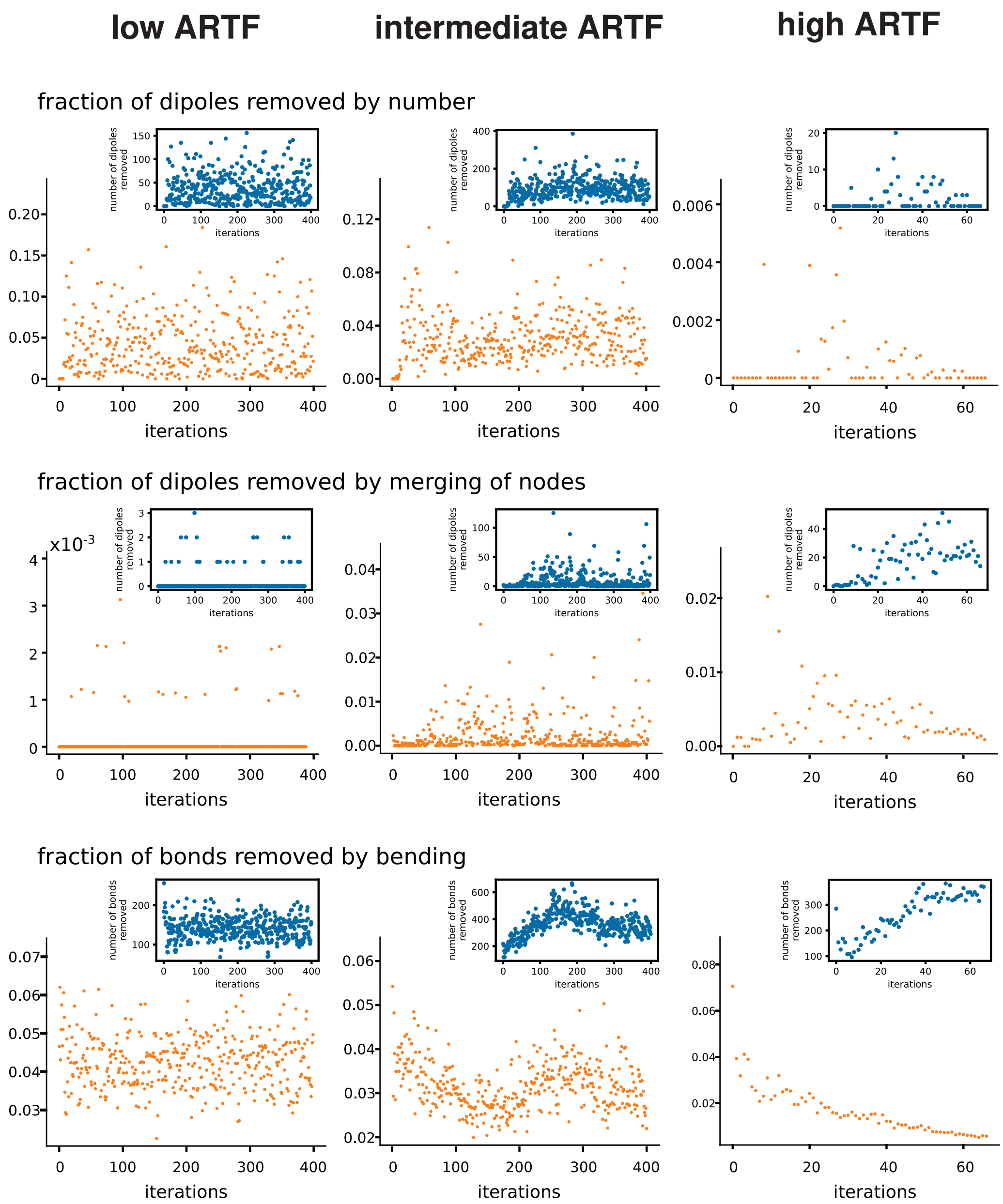

Fig B: Time evolution of the relative contributions of the different network disassembly mechanisms (dipoles and bonds): dipoles removed by number (top), dipoles removed by merging, and bonds removed by bending for low (left column), intermediate (middle column), and high (right column) ARTF ratios. Insets display the corresponding absolute quantities.

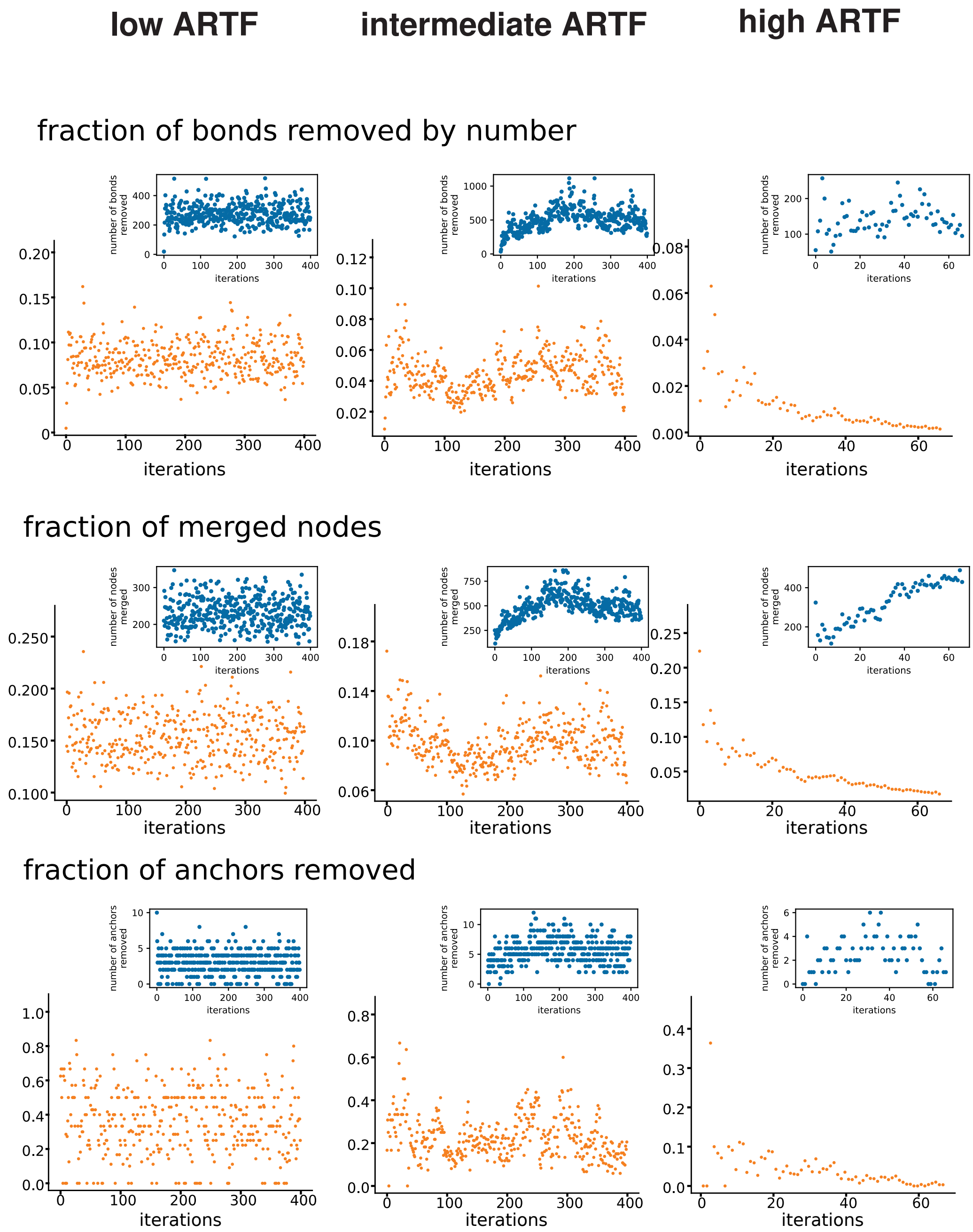

Fig C: Time evolution of the relative contributions of the different network disassembly mechanisms (bonds, nodes and anchors): bonds removed by number (top), merged nodes, and removed anchors for low (left column), intermediate (middle column), and high (right column) ARTF ratios. Insets display the corresponding absolute quantities.

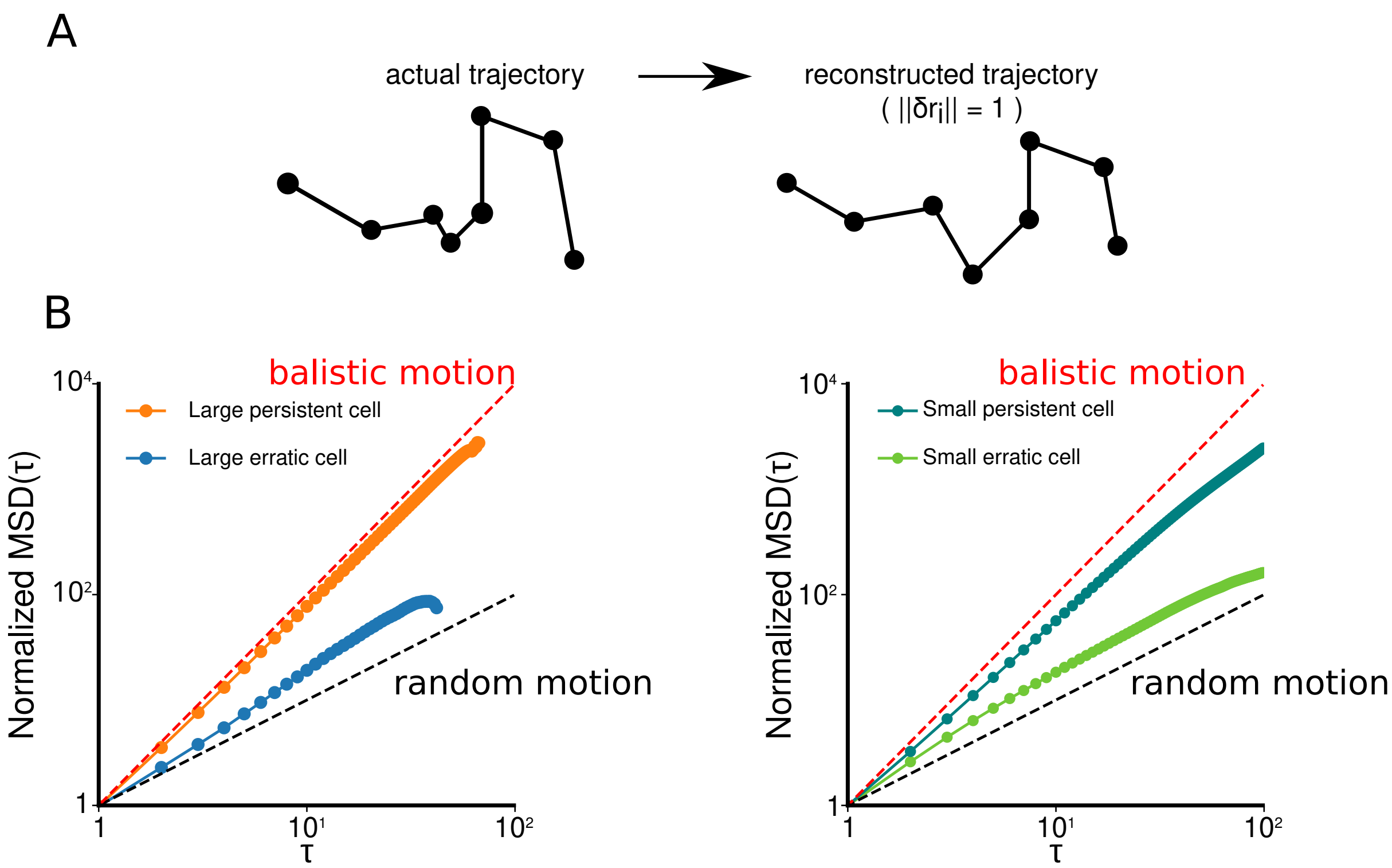

Fig D: Mean Squared Displacement (MSD) of normalized trajectories. A) Sketch of the construction of a normalized trajectory. B) MSD(τ) for representative large (left) and small cells (right). Both large and small cells display persistent (orange, dark green) or random-like trajectories (blue, light green). The bounds for ballistic motion ($MSD_{ballistic}$, red) and random motion (MSD_{random}, black) are shown for reference.

Area under the curve of the normalized mean-squared displacement is used to compute the straightness of the normalized trajectory (SNT) as follows:

$$\mathrm{SNT} = \frac{\mathcal{A}^{MSD}_{sim} - \mathcal{A}^{MSD}_{\mathrm{random}}}{\mathcal{A}^{MSD}_{\mathrm{ballistic}} - \mathcal{A}^{MSD}_{\mathrm{random}}}$$

where $\mathcal{A}^{\mathrm{MSD}}$ represents the area under the MSD curves for the simulated cells, ballistic motion, or random motion. Consequently, if the cell trajectory is a straight line, $\mathrm{SNT} = 1$, whereas if the trajectory is random, $\mathrm{SNT} = 0$.

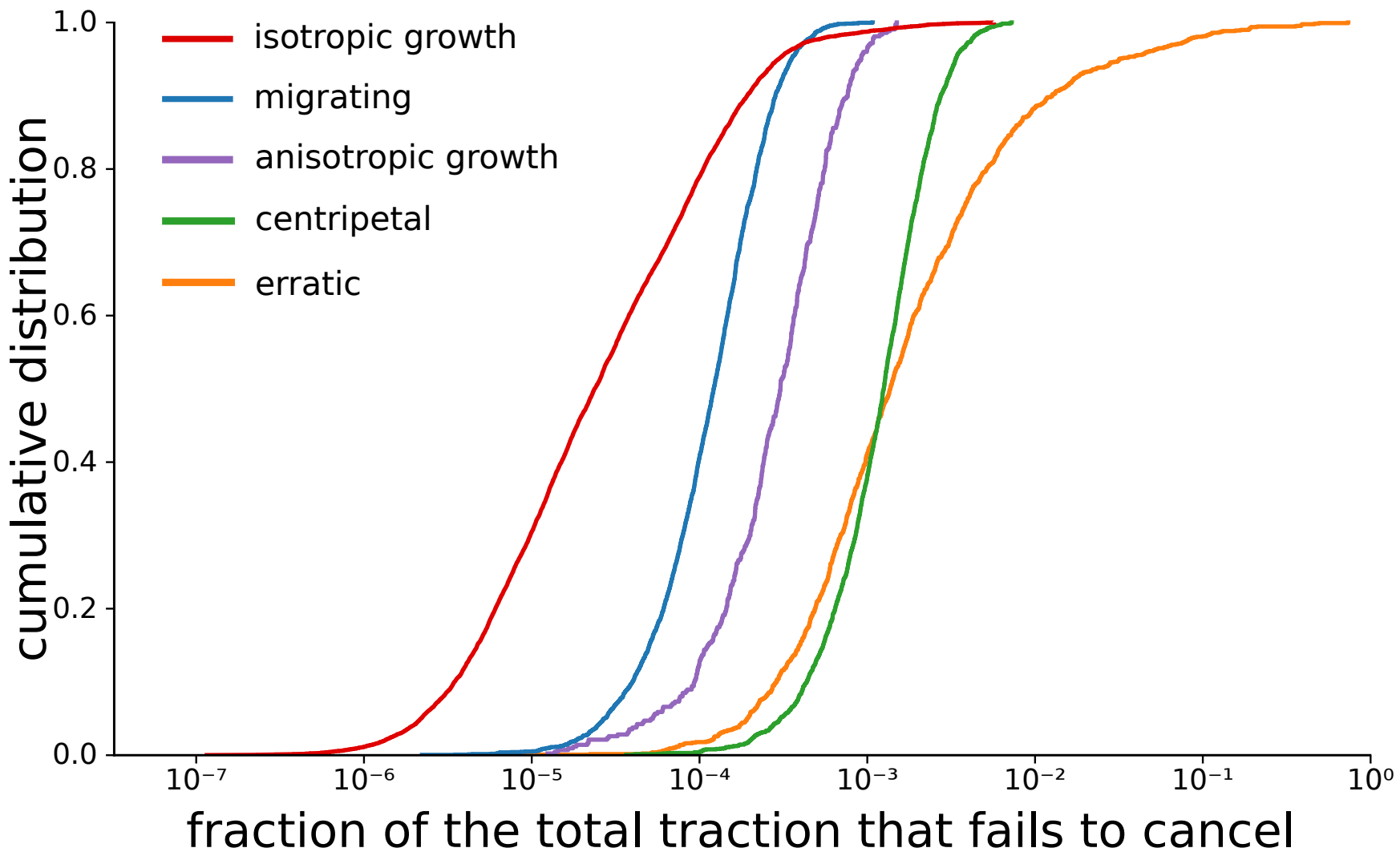

Fig E: Cumulative distribution of the fraction of forces failing cancellation for the different phenotypes: isotropic growth (red), migrating (blue), anisotropic growth (purple), centripetal (green), erratic (orange).

Text F. Fraction of forces failing cancellation

We computed, for every frame of every simulation, the dimensionless residual

$$r = \frac{\left|\sum_i \vec{F}_i\right|}{\sum_i \left|\vec{F}_i\right|}$$

r represents the fraction of the total traction that fails to be canceled. By definition r is bounded in $[0, 1]$ with $r = 0$ corresponding to an exact balance (force-free system) and $r = 1$ corresponding to no cancellation at all (all forces are aligned). Note that the residual is more broadly distributed in regimes with very few anchors per frame (erratic). For this phenotype, with few tractions, the relative residual does not average down, so the upper tail reflects anchor count rather than poorer convergence. Statistics and cumulative distribution for the dimensionless residuals for different phenotypes are summarized in Table B and Fig E respectively.

Table B: Statistics of the distribution for the different phenotypes

**Phenotype**	**Median**	**95th**	**99th**
Erratic	$\sim 2 \times 10^{-3}$	$\sim 3 \times 10^{-1}$	8×10^{-1}
Centripetal	$\sim 2 \times 10^{-3}$	$\sim 3 \times 10^{-3}$	$\sim 5 \times 10^{-3}$
Anisotropic growth	$\sim 3 \times 10^{-4}$	$\sim 9 \times 10^{-4}$	$\sim 10^{-3}$
Migrating	$\sim 10^{-4}$	$\sim 3 \times 10^{-4}$	$\sim 5 \times 10^{-4}$
Isotropic growth	$\sim 2 \times 10^{-5}$	$\sim 3 \times 10^{-4}$	$\sim 10^{-3}$
All sims (pooled)	$\sim 8 \times 10^{-4}$	$\sim 3 \times 10^{-1}$	$\sim 9 \times 10^{-1}$

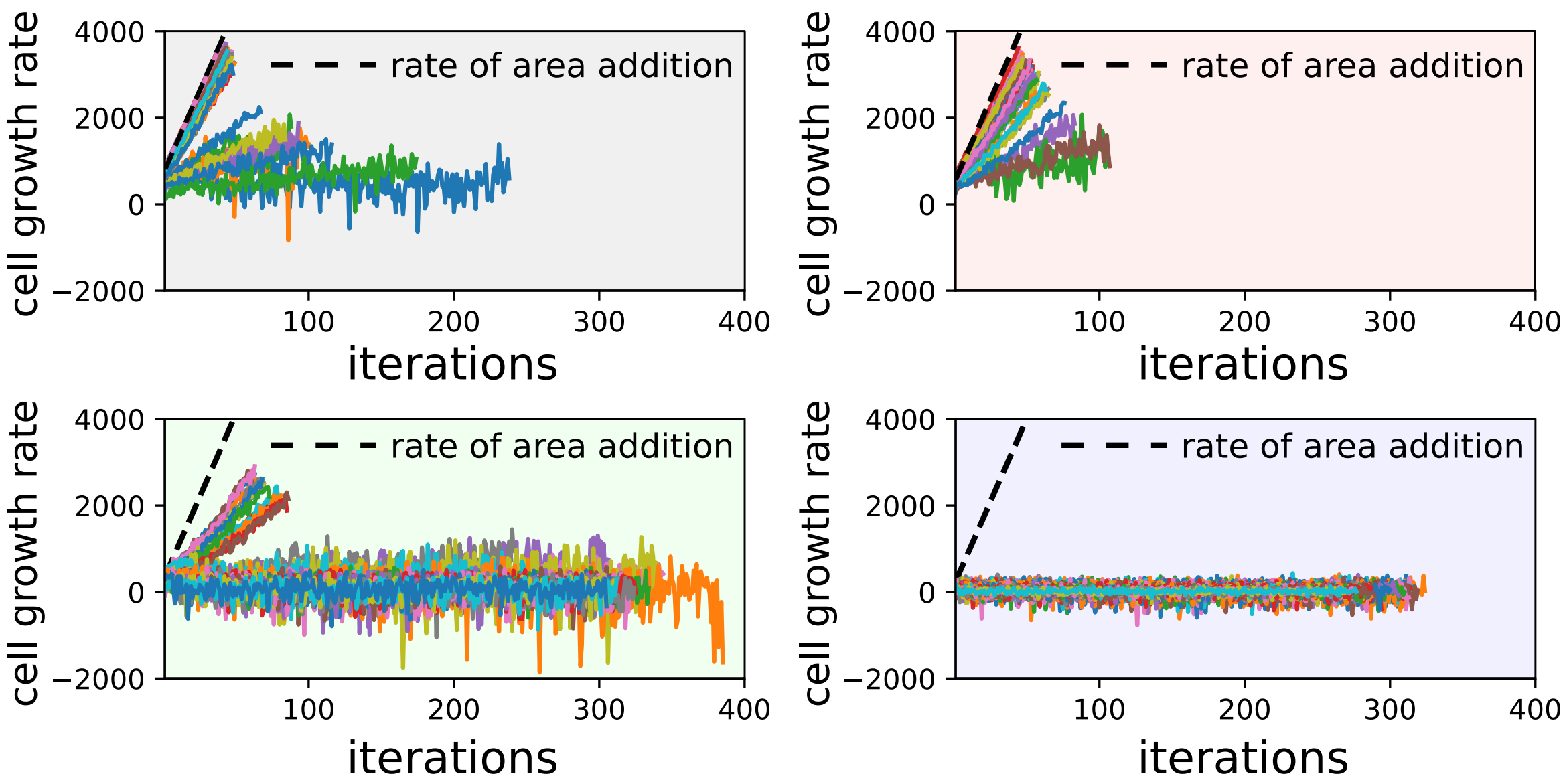

Fig F: Time evolution of the effective cell growth rate. The four panels are the same as in Fig 2. The dashed line corresponds to the rate of area addition due to protrusion.

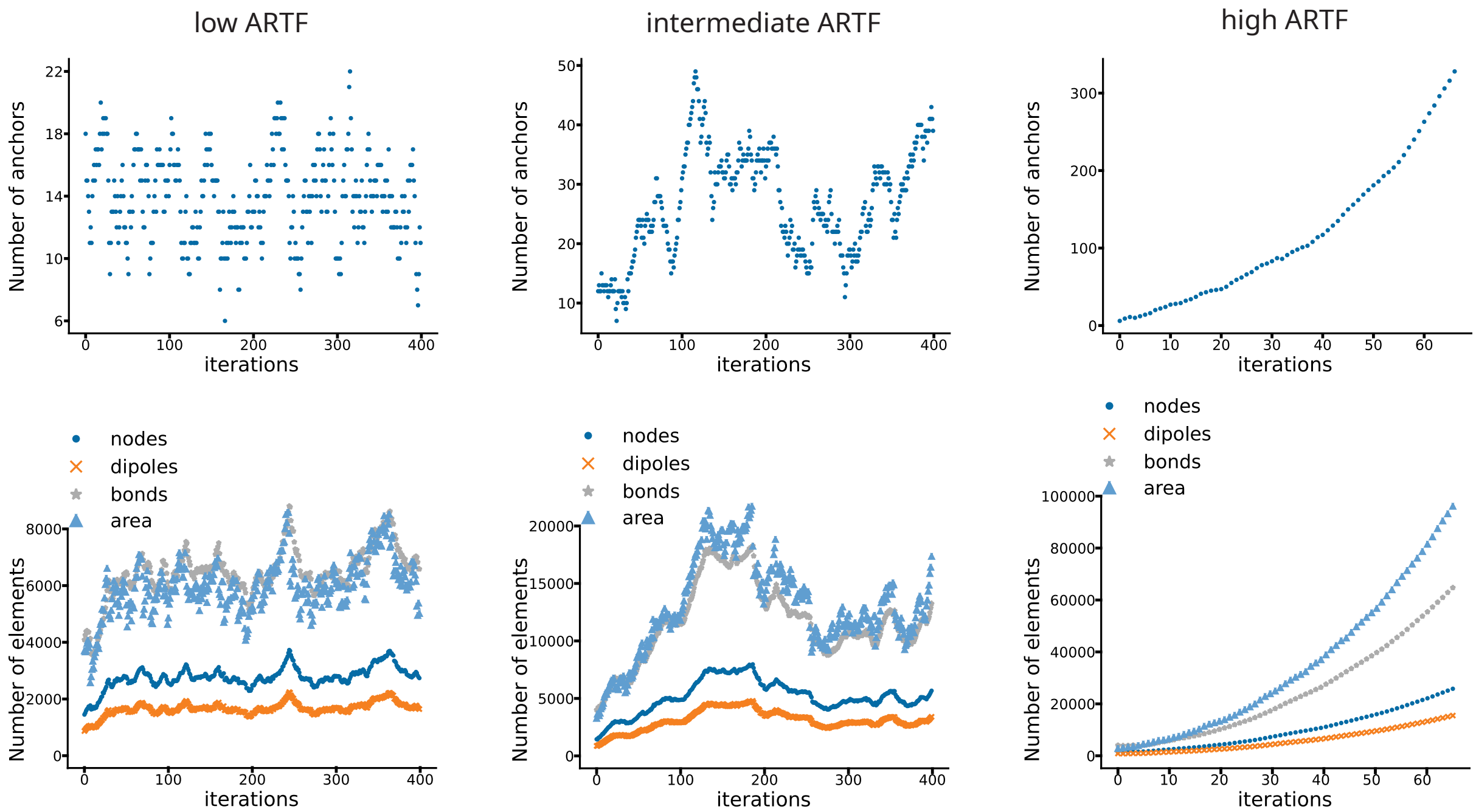

Fig G: Time evolution of the number of elements in the networks for low (left column), intermediate (middle column) and high ARTF ratios (right column). Top panels are the number of anchors, the lower panels are all the other elements: nodes, bonds, dipoles, and cell area.

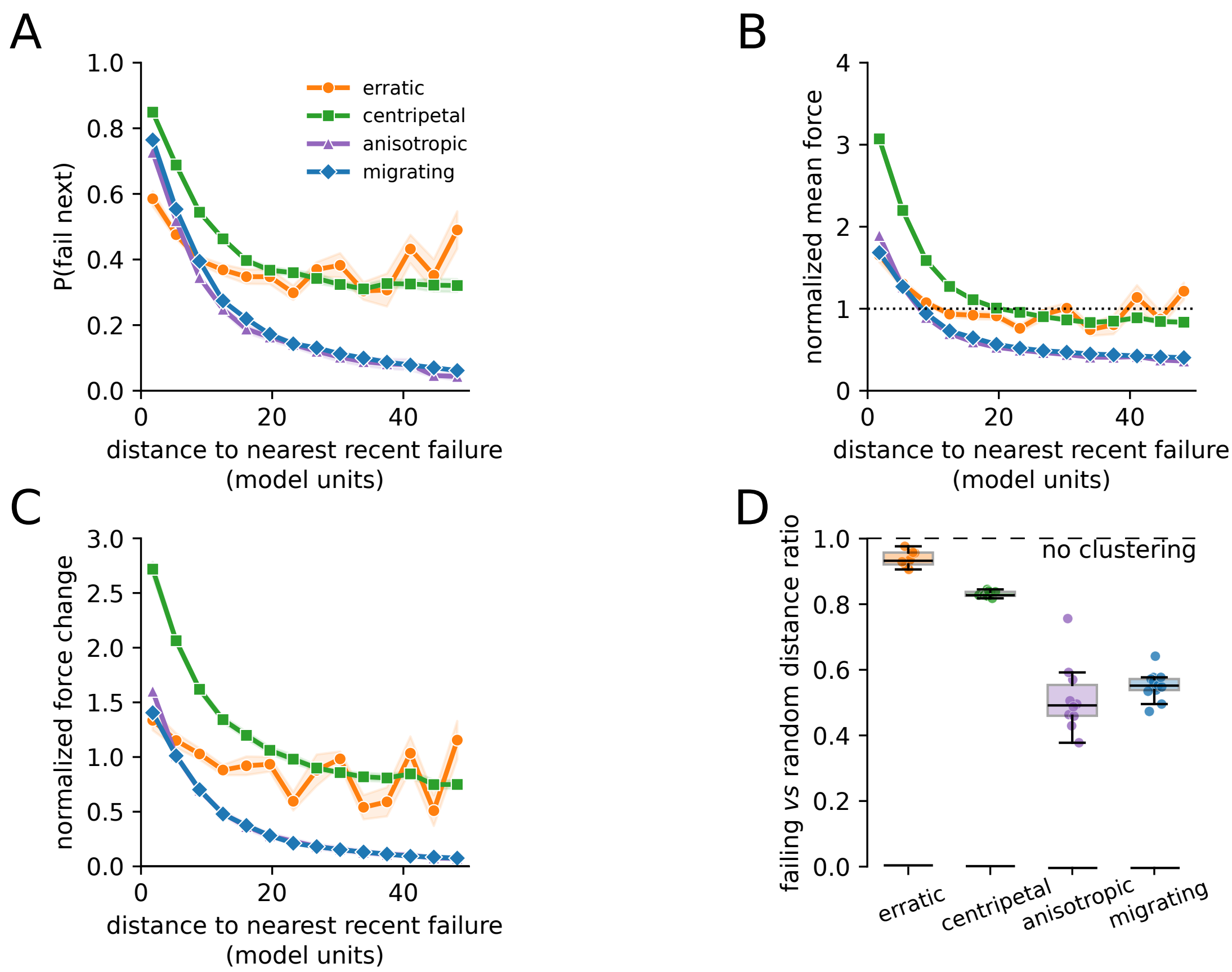

Fig H: Spatial analysis of anchor removal, shown for four phenotypes overlaid and ordered by increasing ARTF ratio: erratic (orange), centripetal (green), anisotropic growth (purple) and migrating (blue). **A** Probability that an anchor is removed in the next step, as a function of its distance to the nearest recent failure. **B** Mean anchor force, normalized by the removal threshold (dotted line at 1), versus the same distance. **C** Redistribution excess: the change in a surviving anchor's force across the step in which a neighbor fails ($\frac{\Delta F}{T_a}$), after subtracting, for each simulation, its own far-field value. **D** Failing-versus-random clustering: per simulation, the ratio of the mean distance-to-recent-failure of the anchors that fail to that of a random, frame-matched sample of the same size; values below the dotted line (ratio = 1) indicate that failing anchors lie closer to recent failures than expected by chance. Box plots show the across-replicate distribution (median, interquartile range, whiskers) with one point per simulation. Each phenotype has 10 replicates.

Text G. Spatial analysis of adhesion failures

This analysis asks whether adhesion failures are organized in space and time: whether an anchor is more likely to be removed when it sits close to one that has just failed, and whether the load released by a failure is picked up by its surviving neighbors. For each phenotype we analyzed the representative simulation together with all parameter-matched replicates. Panels A–C report a quantity averaged within distance bins, computed independently per simulation and then averaged across replicates (error bars: standard error across simulations); panel D reports a single summary number per simulation. Throughout, we discarded the transient and kept the second half of every run.

Detecting failures. Let $\mathcal{A}_t$ be the set of anchors present at frame t and $\mathbf{x}_j^t$ the position of anchor j. We follow anchors from one frame to the next by matching each to its nearest neighbor, with a tolerance $\epsilon = 0.1$ model units, far below the typical spacing between anchors (repeating the analysis with $\epsilon = 1$ left the results unchanged). An anchor present at frame $t-1$ is counted as having *failed* over $t-1 \to t$ if no anchor lies within ϵ of it at frame t – that is, if it has disappeared. The failures of that step form the set

$$\mathcal{R}_t = \Big\{ k \in \mathcal{A}_{t-1} \ : \ \min_{j \in \mathcal{A}_t} \big\|\mathbf{x}_k^{t-1} - \mathbf{x}_j^t\big\| > \epsilon \Big\}.$$

A disappearance in this sense is the removal of an adhesion whose traction had exceeded the threshold T_a. We kept only the steps in which at least one anchor failed ($\mathcal{R}_t \neq \varnothing$).

Distance to a recent failure. For every anchor still present at frame t, we measured how close it is to the most recent failure,

$$d_j(t) = \min_{k \in \mathcal{R}_t} \big\|\mathbf{x}_j^t - \mathbf{x}_k^{t-1}\big\|,$$

the distance from the anchor's current position to the last position of the nearest anchor that just disappeared. We also recorded whether that anchor itself fails at the following step, as an indicator that is 1 if it is gone by frame $t+1$ and 0 otherwise:

$$I_j(t) = \begin{cases} 1, & \min_{\ell \in \mathcal{A}_{t+1}} \big\|\mathbf{x}_j^t - \mathbf{x}_\ell^{t+1}\big\| > \epsilon, \\ 0, & \text{otherwise.} \end{cases}$$

Surviving anchors (for panel C). For the redistribution analysis we also used the anchors that *survived* $t-1 \to t$,

$$\mathcal{S}_t = \Big\{ k \in \mathcal{A}_{t-1} \ : \ \min_{j \in \mathcal{A}_t} \big\|\mathbf{x}_k^{t-1} - \mathbf{x}_j^t\big\| \leq \epsilon \Big\},$$

each matched to its image $m(k)$ at frame t. For a survivor, the distance to the nearest recent failure is taken in the pre-failure frame,

$$d_k(t) = \min_{r \in \mathcal{R}_t} \big\|\mathbf{x}_k^{t-1} - \mathbf{x}_r^{t-1}\big\|,$$

because the redistribution happens across $t-1 \to t$; since a survivor moves less than ϵ between frames, this coincides with the frame-t distance used elsewhere to within ϵ, well below the bin width.

Forces. The force $F_i(t)$ on an anchor is read directly from the simulation and normalized by the removal threshold, $f_i(t) = F_i(t)/T_a$, so that phenotypes are comparable and $f = 1$ marks the threshold. Isotropic-growth phenotypes, in which anchors essentially never reach the threshold,

produce no failures and were excluded.

For a bin B we write $\langle \cdot \rangle_{d \in B}$ for the average of a per-anchor quantity over all anchor–frame pairs (t, j) whose distance $d_j(t)$ falls in B. Panels A–C are all this same bin-average, applied to three different quantities.

Panel A – probability of failing next. The failure probability in bin B is the fraction of anchors in that bin that disappear at the next step, i.e. the bin average of the indicator:

$$P(\text{fail next} \mid d \in B) = \big\langle I_j(t) \big\rangle_{d \in B}.$$

Because $I_j(t)$ is 0 or 1, this is simply the number of anchors in the bin that fail next divided by the number present.

Panel B – mean force. Applying the same average to the normalized force,

$$\langle f \mid d \in B \rangle,$$

shows whether anchors near a recent failure carry a larger fraction of the removal threshold than those farther away.

Panel C – redistribution excess. For each survivor we take the change in its normalized force across the step,

$$\delta_k(t) = \frac{F_{m(k)}(t) - F_k(t-1)}{T_a},$$

and average it within bins, $\langle \delta \mid d \in B \rangle$. Anchors are loaded even far from any failure, so we plot the *excess* over each simulation's own far-field level: δ_{far} is the average of $\langle \delta \mid d \in B \rangle$ over the bins beyond 60% of the distance range, and panel C shows

$$\delta_{\text{excess}}(B) = \langle \delta \mid d \in B \rangle - \delta_{\text{far}}.$$

This far-field level is the ambient per-step loading due to turnover, so the excess isolates the extra load associated with a nearby failure.

Panel D – clustering of failures. Finally, we ask whether the anchors that fail are closer to recent failures than chance would predict. Let $\langle d \rangle_{\text{failing}}$ be the mean distance-to-recent-failure of the anchors that fail at the next step,

$$\langle d \rangle_{\text{failing}} = \frac{\sum_t \sum_{j \in \mathcal{A}_t} I_j(t)\, d_j(t)}{\sum_t \sum_{j \in \mathcal{A}_t} I_j(t)},$$

i.e. the average of $d_j(t)$ over the failing anchors. As a chance reference we use the mean distance over *all* anchors present, giving each frame the same weight it carries above (its number of next-step failures $n_f(t) = \sum_{j \in \mathcal{A}_t} I_j(t)$):

$$\langle d \rangle_{\text{random}} = \frac{\sum_t n_f(t)\, \bar{d}(t)}{\sum_t n_f(t)}, \qquad \bar{d}(t) = \frac{1}{|\mathcal{A}_t|} \sum_{j \in \mathcal{A}_t} d_j(t).$$

Panel D reports their ratio, one value per simulation,

$$R = \frac{\langle d \rangle_{\text{failing}}}{\langle d \rangle_{\text{random}}} :$$

$R < 1$ means the anchors about to fail sit closer to recent failures than expected by chance (spatial clustering), while $R \approx 1$ means no clustering. The per-simulation values are shown as box plots with one point per simulation.

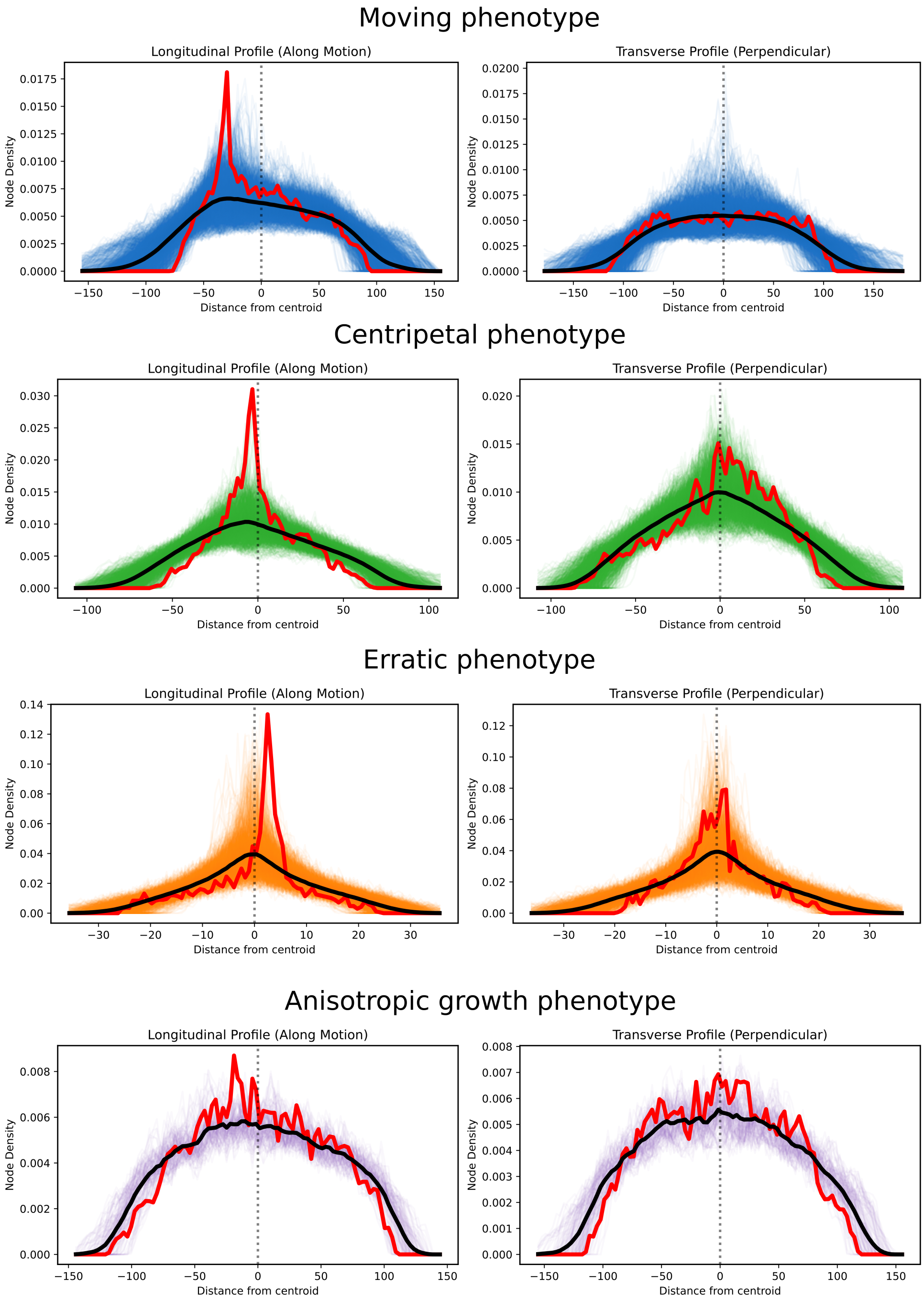

Fig I: Spatial density profiles relative to the axis of motion. For each phenotype, cell density distributions are plotted along the longitudinal axis (parallel to the direction of migration, left column) and the transverse axis (perpendicular to migration, right column). The direction of motion for each iteration t was defined by the displacement vector of the geometric cell centroid from $t-20$ to $t+20$. Distances are shown in absolute model units relative to the centroid (vertical dotted line at 0). Each semi-transparent line represents lowerian individual density profile of a single cell iteration across all replicates. Solid black curves represent the mean density profile for that phenotype, averaged over all individual profiles. The red lines illustrate the specific individual frame exhibiting the highest peak in longitudinal node density.

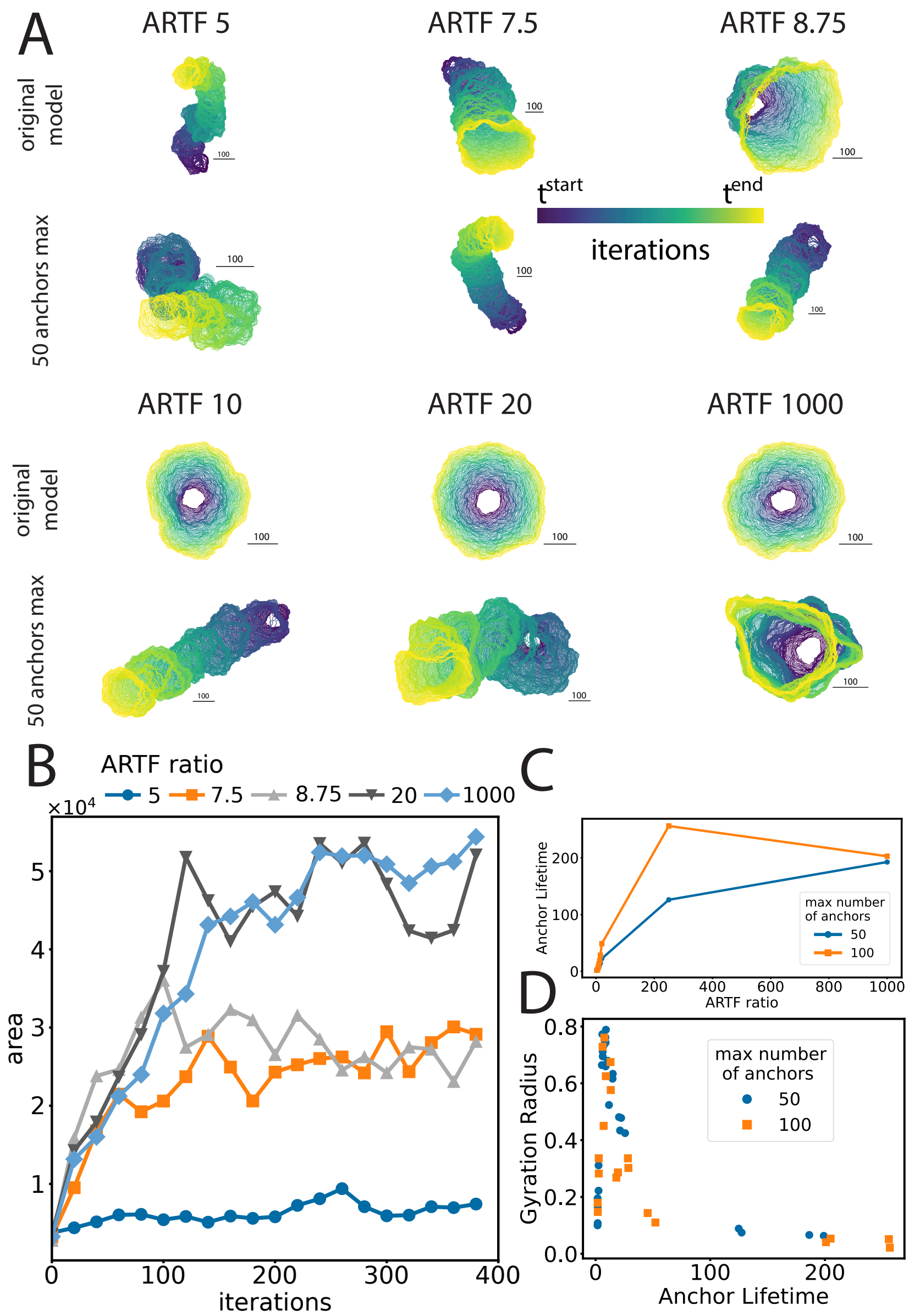

Fig J: A limited resource is not required for polarization. The unconstrained dynamics leads to self-polarization and emergent turnover of material, but a finite adhesion pool may help and prevent unbounded growth. Analysis of a model with a fixed maximum number of anchors. A. Comparison of the behavior of the cell between the original model and a model with a maximum number of 50 anchors. Outlines are color-coded for iterations. Scale bar in dimensionless model length units. B. Representative curves of the evolution of the area of the cell for a model with a maximum of 50 anchors for different values of the ARTF ratio. C. Anchor lifetime as a function of the ARTF ratio for the model with a maximum number of anchors for two different values of the maximum number of anchors in the cell (50 and 100). D. Gyration radius as a function of the anchor lifetime for the model with a maximum number of anchors for two different values of the maximum number of anchors in the cell (50 and 100). The parameters for the simulations are: anchor density $\rho_a = 1$, dimensionless dipole force magnitude $M = 4$, anchor removal threshold $T_a \in \{20, 30, 35, 40, 80, 4000\}$.

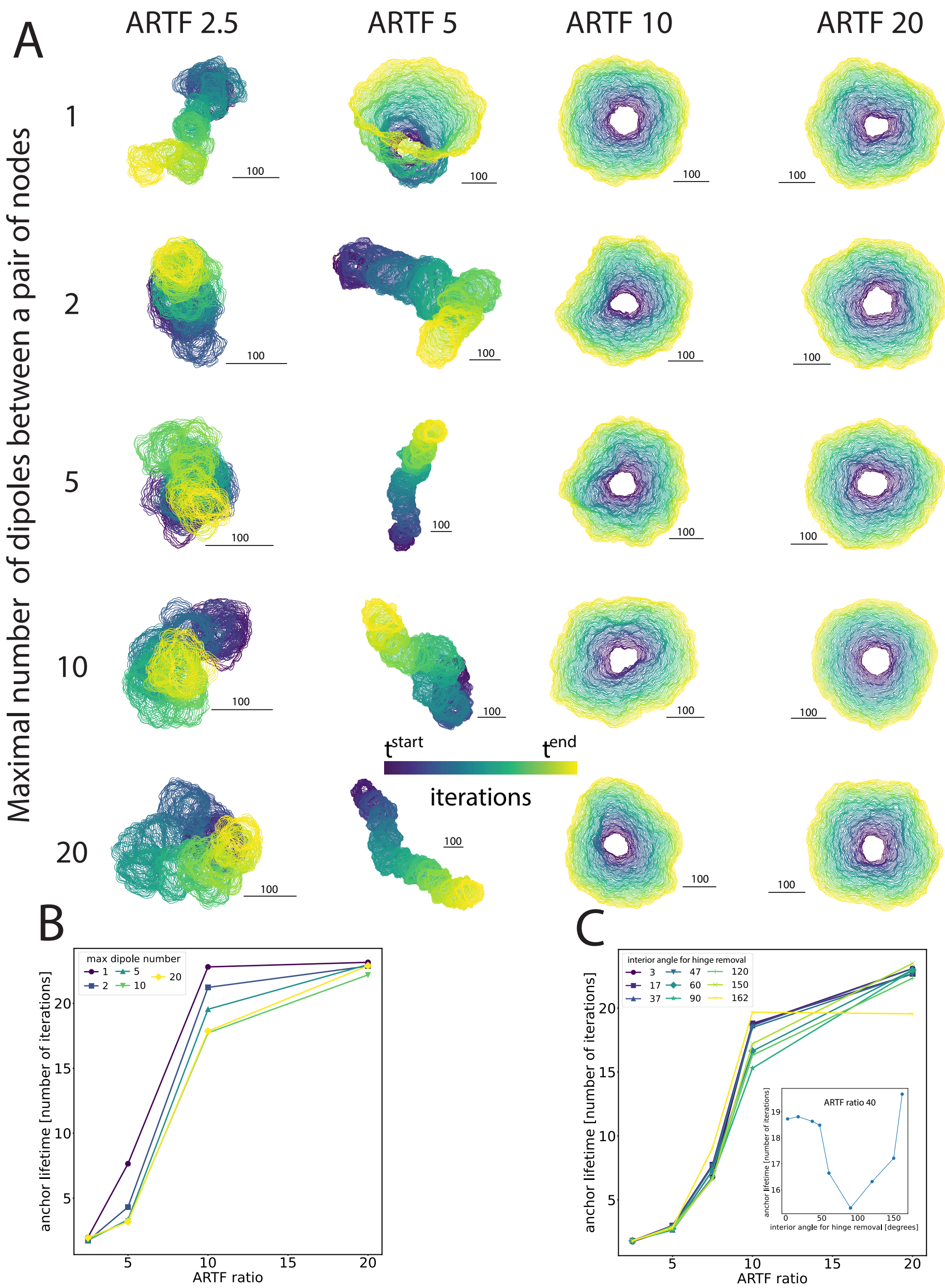

Fig K: Simulations varying the maximal number of dipoles between a pair of nodes and the interior angle for hinge removal. A. Representative examples of the behavior of the cell between different values of the maximal dipole number for different values of the ARTF ratio. Outlines are color-coded for iterations. Scale bar in dimensionless model length units. B. Anchor lifetime as a function of the ARTF ratio for different values of the maximal dipole number. C. Anchor lifetime as a function of the ARTF ratio for different values of the interior angle for hinge removal. Inset. Plot of the anchor lifetime as a function of the interior angle for hinge removal for a fixed ARTF ratio (40). The parameters for the simulations are: anchor density $\rho_a = 1$, dimensionless dipole force magnitude $M = 4$, anchor removal threshold $T_a \in \{10, 20, 30, 40, 80\}$.

References

[1] Ronceray P, Broedersz CP, Lenz M. Fiber networks amplify active stress. Proc Natl Acad Sci U S A. 2016;113(11):2827–2832.

[2] Messi Z, Bornert A, Raynaud F, Verkhovsky AB. Traction Forces Control Cell-Edge Dynamics and Mediate Distance Sensitivity during Cell Polarization. Current Biology. 2020;30(9):1762–1769.e5. doi:10.1016/j.cub.2020.02.078.

[3] Broedersz CP, MacKintosh FC. Modeling semiflexible polymer networks. Rev Mod Phys. 2014;86:995–1036. doi:10.1103/RevModPhys.86.995.

[4] Hu S, Dasbiswas K, Guo Z, Tee YH, Thiagarajan V, Hersen P, et al. Long-range self-organization of cytoskeletal myosin II filament stacks. Nature Cell Biology. 2017;19(2):133–141. doi:10.1038/ncb3466.

[5] De La Cruz EM, Gardel ML. Actin Mechanics and Fragmentation*. Journal of Biological Chemistry. 2015;290(28):17137–17144. doi:https://doi.org/10.1074/jbc.R115.636472.

[6] Gittes F, Mickey B, Nettleton J, Howard J. Flexural rigidity of microtubules and actin filaments measured from thermal fluctuations in shape. Journal of Cell Biology. 1993;120(4):923–934. doi:10.1083/jcb.120.4.923.

[7] Berro J, Michelot A, Blanchoin L, Kovar DR, Martiel JL. Attachment Conditions Control Actin Filament Buckling and the Production of Forces. Biophysical Journal. 2007;92(7):2546–2558. doi:https://doi.org/10.1529/biophysj.106.094672.

[8] Kovar DR, Pollard TD. Insertional assembly of actin filament barbed ends in association with formins produces piconewton forces. Proc Natl Acad Sci U S A. 2004;101(41):14725–14730. doi:10.1073/pnas.0405902101.

[9] Gabella C, Bertseva E, Bottier C, Piacentini N, Bornert A, Jeney S, et al. Contact Angle at the Leading Edge Controls Cell Protrusion Rate. Current Biology. 2014;24(10):1126–1132. doi:10.1016/j.cub.2014.03.050.

[10] Carlier MF, Pantaloni D. Control of actin dynamics in cell motility11Edited by N.-H. Chua. Journal of Molecular Biology. 1997;269(4):459–467. doi:https://doi.org/10.1006/jmbi.1997.1062.

[11] Cameron LA, Svitkina TM, Vignjevic D, Theriot JA, Borisy GG. Dendritic Organization of Actin Comet Tails. Current Biology. 2001;11(2):130–135. doi:10.1016/S0960-9822(01)00022-7.

[12] Arai Y, Yasuda R, Akashi KI, Harada Y, Miyata H, Kinosita J Kazuhiko, et al. Tying a Molecular Knot with Optical Tweezers. Nature. 1999;399(6735):446–448. doi:10.1038/20894.

[13] Murrell MP, Gardel ML. F-actin buckling coordinates contractility and severing in a biomimetic actomyosin cortex. Proceedings of the National Academy of Sciences. 2012;109(51):20820–20825. doi:10.1073/pnas.1214753109.
