# Supplementary material for: Balanced contractility and adhesion drive polarization in a minimal elastic actomyosin network
Source: PLoS Comput Biol. 2026 Sep 18;22(9):e1014750. doi: 10.1371/journal.pcbi.1014750 (PMC13630322; doi:10.1371/journal.pcbi.1014750)
Supplement: Supplementary file 2 [file pcbi.1014750.s012.pdf]

Recommendation: Major Revisions

Summary of Article contributions:

The authors extend a computational discrete active network model -- representing cellular actomyosin networks -- to the case of dynamic adhesions, exploring how symmetry breaking in and persistent migration of elastic cytoskeletal networks can originate purely through contraction-adhesion dynamics. The computational cell model incorporates a simple rule: anchors (representing adhesions) break under a threshold force. The authors observe five distinct phenotypes emerging as a function of anchor density and the ratio of anchor removal threshold to dipole force magnitude (ARTF): small networks moving erratically, large networks with centripetal contraction, migrating networks, networks growing isotropically, and networks growing anisotropically.

Importantly, the migrating phenotype, which occurs at intermediate ARTF ratios, features characteristics associated with persistently migrating cells: e.g., network shape, the localization of force dipoles in the rear, and the localization of anchors in the front. The authors illuminate that the significance of the ARTF ratios lies in its impact on anchor lifetime, as the ARTF ratio defines the number of dipoles required to break an anchor. As such, the emergence of a migrating phenotype at intermediate ARTF suggests an optimal anchor lifetime. In further evidence of the significance of the balance of contraction and adhesion for network dynamics, the authors demonstrate that the memory for polarization, and thus directionality, can only be lost by perturbing *both* the dipole and anchor distributions in the network.

Finally, the authors demonstrate that the model can recapitulate symmetry breaking due to adhesion-associated migration cues (haptotaxis / durotaxis) by introducing a gradient of the ARTF ratio, resulting in highly directional and persistent migration for both the migrating phenotype and erratic phenotype. In all, this work highlights that polarization can be an emergent behavior of cytoskeletal-like networks due to mechanics alone.

Summary of Reviewer impression:

This work appears to be novel. The computational approach employed by the authors is well motivated, as it contributes to the broader understanding of how cytoskeletal mechanics contributes to cell polarity and migration by isolating the contributions of actin, myosin, and adhesion from *in vivo* complexity (signaling, upstream regulation, biomechanical feedback, etc.). The authors extend a previous discrete active network model, yielding biologically relevant conclusions regarding the important balance of adhesion and contraction dynamics in cells. As such, the results of this manuscript would certainly be of interest to the readership of PLOS Computational Biology. However, the authors need to clarify model and computational details and, importantly, distinguish novel contributions from those of prior work. My comments are as follows:

Major Revisions:

1. In the computational discrete active network model, it is essential that the authors clarify and emphasize this work's particular novel contributions. This model (largely the same as the author's prior work in [2]) is an extension of the model in reference [32] (itself building off [36]), as noted in Lines 40-43 and 66-68. However, the language in this manuscript suggests that the model development occurred in *this* project (e.g., in the Author Summary, "we developed a computational model of a simplified cell made only of elastic connections, contractile forces, and attachment points to its surroundings"). This is also the impression given by the methods section, where the computational cell model is rederived despite already largely being described in [2], [32], and [36]. I am not convinced that the extensive model description in the methods section adds value to this manuscript – if the authors want to keep this for completeness, aspects which are genuinely novel should be emphasized over aspects which are the same as [2], [32], or [36]. It would also be helpful for the authors to clarify how the model was used in [2] and [32] (which would strengthen the argument for its usefulness in describing mechanical actomyosin dynamics in cells).
2. The description of the model in the methods section is difficult to understand due to a lack of clarity in terminology which must be addressed:
    a. The words "bond", "segment", and "filament" seem to be used interchangeably or are simply poorly defined. For example, in Lines 81-83, segments are noted as inextensible but filaments are allowed to stretch – this seems contradictory to the note in Line 68 about filaments being composed of chains of inextensible segments. This issue probably also relates to a confusion regarding differences between this work and prior work in [36] (relating to major revision comment #1). This confusion also arises in the discussion of rest length, where bonds have rest length 2\ell_0 (Line 71), segments have rest length \ell_0 (Line 84), and filaments have rest length \ell_p (Line 102) which is also persistence length (Line 69) -- I suspect this last one is a typo, but of course adds to confusion.
    b. Similarly to above, it is unclear what the difference in energy contribution is between filaments which are bent (eq. 2) and "hinges" (Line 77), especially because the full Hamiltonian in eq. 14 only incorporates "hinges". Presumably filaments are composed of several segments which all form hinges, but this is not clearly stated.
    c. In Line 102, is the length of myosin bipolar minifilaments defining bond/segment rest length \ell_0? If so, this should be clarified.
    d. A minor related comment: Is the range of dipole force magnitudes listed in Line 109 in pN units or in nondimensional model units?
3. Pruning of the hinges, nodes, and bonds can in principle result in effective turnover of the actin network, which would be a nontrivial contribution to the dynamics. The

authors need to report how much pruning of the network (removal of hinges, merging of nodes, rearranging and removal of bonds) happens over the course of simulations for different conditions. I suspect that highly migratory cases (particularly those which stay below the area threshold) may have high rates of network pruning that would correspond to non-negligible levels of network turnover (e.g., it seems true in Movie 1 where the network density does not appear to change with time, and also in Movie 10 after the half-way point). Further, the authors should then compare the rate of pruning to *in vivo* rates of actin turnover.

4. In Lines 292-301, the authors note that large cells exhibit low persistence scores, but they appear more persistent because of a subtlety regarding actual displacement in comparison to cell size. As such, Figure 2D is incredibly misleading, where several large cells exhibit high persistence according to the chosen measure. Moreover, since the simulation time for large cells is significantly shorter than for small cells, it is not clear to me that their persistence is comparable in the manner presented in this work.
    a. To at least clarify Fig 2D, the authors could consider adding the cell boundary outline at the end of trajectories in Fig 2E (if this change makes the figure too large, Fig. 2E could become a supplementary figure).
    b. Otherwise, in discussing the difference in persistence of large versus small cells, the authors should address how this comparison (and its usefulness) is impacted by the difference in simulation time necessitated by the unbounded growth inherently defining large cells.
    c. Relatedly, perhaps a useful plot would be persistence score vs ARTF ratio, given the high correlation shown in Fig 2B and then explored in Figs 3-5. I suggest the authors add this to Fig 2, unless it would be misleading in a manner similar to Fig 2D.
5. For Figures 3-5 as well as Movies 1-10, the authors should report which parameters are used to generate the displayed characteristic features/movements (specifically, the parameters which distinguish between the total 150 parameter sets explored).

Minor Revisions:

1. There are several typos throughout the manuscript which should be addressed:
    i. Lines 28-29: "[A] Discrete elastic model of actin-myosin network investigated the effect [of] nonlinear actin elasticity on force transmission...."
    ii. Lines 30-31: "To investigate polarization, other studies [described the] actin-myosin network as..."
    iii. Lines 38-39: missing ")"
    iv. Line 102: "(other refs?)"
    v. Line 167: "we considered a restarted the simulation"

vi. Line 196: “Network flowwas”
vii. Line 296-297: “small cells exhibiting either erratic or persistent motion, expanding but stationary cells, and larger...”
viii. Line 386: missing “)”

2. In this work, the authors constrain their focus to lamellipodium-based mesenchymal migration. However, the manuscript seems to imply that this is the only (relevant) migration mode, neglecting bleb-based (ameboid) migration which is prevalent in 3D microenvironments. The authors should clarify this focus on actin protrusion-based migration somewhere in the introduction.
3. The elastic modulus of the substrate is chosen to be 1e6 (“very high”, Line 110). The authors should clarify why they choose this order of magnitude, and whether the results would change qualitatively at lower substrate elastic moduli.
4. In parts of the Methods (Lines 100-106) and Discussion (Lines 518-527) sections, the authors note parameters and resultant scales in space and time which correspond to experimentally measured or physiologically reasonable values in units. I recommend the authors add a column to Table 1 which includes the resultant in-units values and references when those parameters are drawn from the literature. Regardless of if this is added to the table, exactly which parameters were selected based on experimental data versus previous modeling work (as noted in Lines 120-122) should be noted explicitly by the authors.
5. In Lines 117-120, the authors note that parameters can be grouped by whether they are structural or related to turnover/pruning. This could be added to Table 1 as well, though not absolutely necessary.
6. In Lines 263-264, the authors note “see Methods for details” regarding when simulations are stopped. This is never mentioned in the methods section, so it should be either added by the authors or removed from Lines 263-264.
7. In Lines 123-125, the authors state which parameters are allowed to vary (and why) -- it would be helpful to restate or reference this in Lines 268-269 regarding the ensemble of 150 distinct parameter sets used in the Results section. In addition, some results are averaged over identical parameter sets (as noted in the caption of Fig 2), but how many identical parameter sets are considered does seem to be stated – the authors should clarify this detail.
8. In Figure 2C, areas under MSD curves for ballistic, random, and simulation trajectories are shaded for large cells but not small cells. As the shading is not particularly useful and obscures the orange/blue simulation data, I recommend the authors remove the shading and just keep the dotted lines corresponding to ballistic and random MSD bounds. Also, the plots should be labelled as corresponding to large/small cells, instead of only being noted as such in the caption.
9. For the linear kymographs, how is the “line through the approximate center of the system” (Line 186-187) defined? Is it meant to correspond to the migration direction (if there is one)? Relatedly, how is angle = 0 chosen for radial kymographs? In

principle, one would expect angle = 0 to correspond to the migration direction, but this does not seem true throughout Fig 5C.

10. In Fig 6C, the red line = 1 defining anchor removal threshold should be noted as such in the figure caption.
11. In Fig 6D, the effects of varying gradient strength are shown for migrating cells and erratic cells, but for migrating cells two different nonzero gradient strengths are used while for erratic cells only one nonzero gradient strength is used. The authors should clarify whether (and if so, why) the conditions in “on” vs “off” gradient cases are different for the two phenotypes. It would also be valuable to include an intermediate gradient condition for the erratic cells, to aid the comparison in Fig. 6D.
12. In Lines 491-493, the authors comment on how their results show that the balance of contractility and adhesion impacts cell migration speed, but this is not actually analyzed in this manuscript so the authors should refrain from this being a conclusion of this work.
13. There are other computational models exploring actomyosin mechanics questions which do not employ a viscoelastic continuum formulation, e.g., Cytosim, Maxian et al. 2021 (https://doi.org/10.1371/journal.pcbi.1009240), Kim et al. 2009 (https://doi.org/10.1371/journal.pcbi.1000439), and Rutkowski and Vavylonis 2021 (https://doi.org/10.1371/journal.pcbi.1009506). In the introduction, the authors should clarify the benefits and/or differences of their approach over these other types of non-continuum mechanical models of actomyosin networks, for addressing questions relating to polarization.
