# Supplementary material for: Balanced contractility and adhesion drive polarization in a minimal elastic actomyosin network
Source: PLoS Comput Biol. 2026 Sep 18;22(9):e1014750. doi: 10.1371/journal.pcbi.1014750 (PMC13630322; doi:10.1371/journal.pcbi.1014750)
Supplement: Supplementary file 3 [file pcbi.1014750.s013.pdf]

Balanced contractility and adhesion drive polarization in a minimal elastic actomyosin network

(PCOMPBIOL-D-26-00527)

Point-by-point response letter

1 Reviewer #1

In their manuscript the authors propose, based on previous experimental and modeling studies, a minimal model for cell polarization and motility . The model consists of elastic bonds, force dipoles mimicking myosin motor minifilaments and anchors, mimicking adhesion bond formation and that break upon excess of load. Simple turnover rules are applied for the dynamics. Several phenotypes are found, including self-polarized moving "cells" of roughly constant size. The main parameter is identified as the timescale of the anchor detachment. The work suggests that this minimal system is sufficient for polarization, size determination AND motion, which in my opinion is an important step in our understanding of motility. Overall all I like the approach and find especially movie 5 impressive, as it also recovers the shape and the transverse actomyosin bundle observed in crawling keratocytes. The paper should be published after the authors respond to my comments below.

1.1 Comments

1. There are quite some typos and formatting issues (e.g. p7 after eq(14) "(other refs?)"). Please fix them.

   We thank the reviewer for the careful reading. We have carefully proofread the revised version.

2. p8 after Eq(16) "In our simulations, when a single dipole is not capable to bend a bond, the system simply grows" This is hard to grasp at this point in the model description, since growth was not yet mentioned. Maybe first describe the model parts AND the dynamics very briefly before going into the details. This would help the reader

   We thank the reviewer for the comment. We have modified the description of the model; there is no mention of the dynamics of the model in this section.

3. p9, line 140: "Merged nodes are replaced by a new node located at their barycenter carrying all the bonds and dipoles of the pair" I understand the procedure, and it makes sense. But I have the feeling that this may lead to artificial "jumps" in the dynamics. For instance, for simulations of growing tissue/bacterial colonies it is known that replacing a cell by two (here it is rather the inverse, replacing many nodes by a single one) leads to spurious and unrealistic peaks especially in the forces. Did the authors check (eg for small systems) that such issues are not important here?

   This is an important point, and the analogy to colony/tissue models is well taken. We address it by clarifying the mechanism and by inspecting the dynamics of forces.
   The situation differs in kind from cell division in those models, where a finite-sized object is inserted, displacing the configuration discontinuously.

Here merging is the reverse and acts only at very short range: a pair of nodes is fused only once minimization has already driven the two nodes to within d_{merge}. Merging therefore completes a collapse that the dynamics had already produced rather than imposing a new deformation.
The reason why the merging procedure does not generate spurious force peaks is the order of the operations:

(a) Forces are evaluated after minimization at a minimum of the Hamiltonian, $\partial\mathcal{H}/\partial\mathbf{x}_i = 0$,

(b) anchors are removed depending on forces that were computed at equilibrium, prior to pruning, merging and addition of new material,

(c) pruning, merging and addition of new material are performed,

(d) the next minimization step is performed.

With this sequence of events ($a \to b \to c \to d$), any perturbation introduced by a merge is relaxed during the next minimization.
This concern is known in cell-based tissue models, where cell division and removal produce a discontinuous change in the computed forces Fletcher et al. [2014]. It has been examined directly in a vertex model of growing tissue Barton et al. [2017]: although that model integrates equations of motion, so the system can be transiently out of balance between updates, the force discontinuities introduced by division were found to cause no dynamical artifacts or instabilities. Our system is more resilient still to this kind of perturbation, because a merge fuses two already near-coincident nodes rather than inserting or deleting a finite cell, and because our update scheme is quasi-static (the system moves through a succession of equilibrium states).
To address this question, we first quantified spurious force peaks in the distribution of forces. We focused on cells with ARTF ratios 1, 2.5 and 5, that typically result in cells with small and steady area. In brief, for each simulation we recorded the force on each anchor at each iteration, discarding the transient regime, and computed the distribution of forces. We then identified outliers in the distribution based on the Z-score. Each observation with a Z-score greater than 3.5 is considered an outlier. In the following table we report for each ARTF ratio the probability that an anchor is subjected to an outlier force, the mean number of merged nodes and the probability that the number of merged nodes at a given iteration is an outlier with respect to the distribution of the number of merged nodes. We applied the same procedure to identify an unexpected number of merged nodes:

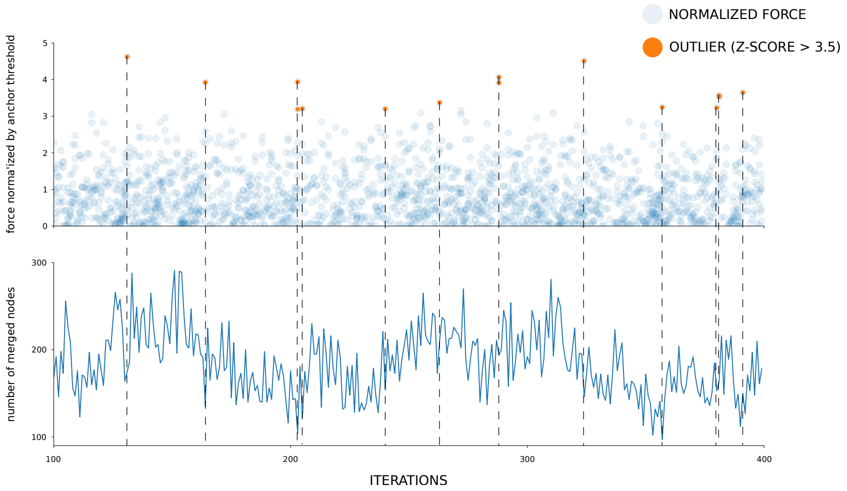

Figure 1: Time evolution of force events and number of merged nodes for a simulation with ARTF ratio 2.5. Upper panel: Normalized forces (blue) and outliers of the normalized forces (orange). Lower panel: Time evolution of the number of merged nodes. The first 100 transient iterations have been discarded.

ARTF	Prob. of force outlier	Mean Merge	Prob. of merge outlier
1.0	0.098961	171.896146	0.003449
2.5	0.037509	172.646778	0.001505
5.0	0.054494	161.745000	0.001003

This table indicates that force outliers are not frequent in general ($\sim 5\%$), while the mean number of merged nodes is about 170, and the probability of a merge event being an outlier is lower than the force outlier probability by more than one order of magnitude. Together, these results strongly suggest that peak forces are unlikely to be the result of a particular node merging (Figure 1). Note that the high number of merged nodes is expected by the design of the model: addition of network $\rightarrow$ minimization $\rightarrow$ forces measurement $\rightarrow$ merging.

4. Concerning the force maps described on p12, did the authors check that the sum of all forces is always zero, as required for a force-free system? This should be mentioned/discussed.

   We thank the reviewer for this remark. Each configuration is brought to equilibrium by energy minimization; because the descent is terminated at a finite tolerance on the energy gradient, a small residual net force might remain. We quantified this residual by computing for every frame of every simulation the dimensionless residual $r = \frac{|\sum_i \vec{F}_i|}{\sum_i |\vec{F}_i|}$, that is to say

the fraction of the total traction that fails to be canceled. By definition r is bounded in $[0, 1]$ with $r = 0$ corresponding to an exact balance (force-free system) and $r = 1$ corresponding to no cancellation at all (all forces are aligned). Its distribution is shown in a new supplementary figure (Supp. Fig. S5). We report the main statistics of the distribution for the different phenotypes and pooled simulations, confirming that the residual remains very small to within the gradient-descent tolerance. As the reviewer asks

**Phenotype**	**Median**	**95th**	**99th**
Erratic	$\sim 2 \times 10^{-3}$	$\sim 3 \times 10^{-1}$	8×10^{-1}
Centripetal	$\sim 2 \times 10^{-3}$	$\sim 3 \times 10^{-3}$	$\sim 5 \times 10^{-3}$
Anisotropic growth	$\sim 3 \times 10^{-4}$	$\sim 9 \times 10^{-4}$	$\sim 10^{-3}$
Migrating	$\sim 10^{-4}$	$\sim 3 \times 10^{-4}$	$\sim 5 \times 10^{-4}$
Isotropic growth	$\sim 2 \times 10^{-5}$	$\sim 3 \times 10^{-4}$	$\sim 10^{-3}$
All sims (pooled)	$\sim 8 \times 10^{-4}$	$\sim 3 \times 10^{-1}$	$\sim 9 \times 10^{-1}$

about the forces summing up to zero, the cancellation is meaningful if expressed relative to the quantity being canceled, that is to say the sum $\sum \left|\vec{F}\right|$. Accordingly, $r = \frac{\left|\sum_i \vec{F}_i\right|}{\sum_i \left|\vec{F}_i\right|}$ quantifies the fraction of forces failing to cancel. Note that the residual is more broadly distributed in regimes with very few anchors per frame (erratic). For this phenotype, with few tractions, the relative residual does not average down, so the upper tail reflects anchor count rather than poorer convergence.

5. p16 l306: here 5 phenotypes are mentioned but afterwards come only three types with subtypes. Maybe restructure for clarity.

   We thank the reviewer for this comment. The five phenotypes are grouped into three subsections by shared underlying dynamics (low ARTF: erratic/centripetal; high ARTF: isotropic/anisotropic growth; intermediate ARTF: migrating). We have rewritten the opening paragraph for clarity.

6. Concerning the isotropic/anisotropic growth discussed on page 17: I find this phenotype also promising. It may be stabilized by some implementation of mass conservation, right? Did the authors try this? Such a stabilization would make the parameter region where polarization/motion is possible substantially larger and the message of the manuscript even stronger (limited resources/membrane are not needed for polarization, as the authors also discuss in the conclusions, but may help it)

   We are grateful for this comment, and according to the reviewer's suggestion we implemented two kinds of conservation: a limited pool of network (actin) and a limited pool of anchors (adhesion).
   limited pool of network: We imposed a global cap on the network, a reservoir that is consumed by addition of new material at the periphery of the cell and replenished by merging and pruning. Limiting the pool of

network does not stabilize the growing phenotypes into a motile phenotype but arrests them, because material is supplied only at the edge and the cap simply starves it. A limited actin pool is therefore neither required for polarization nor, in this naive form, a way to enlarge the motile regime. Note that for a fast, motile cell such as a keratocyte, the actin monomer pool is not the factor limiting protrusion: the diffusible actin concentration exceeds that needed to sustain the observed polymerization rates by orders of magnitude Raz-Ben Aroush et al. [2017], and the relevant homeostasis is a turnover steady state in which assembly balances disassembly Manhart et al. [2019].

limited pool of anchors: As for actin, only a fraction of the total cellular pool of integrin, vinculin and talin is engaged in adhesions Gallant et al. [2005]. Those molecules are not limiting factors; instead, it has been shown that rear detachment is the rate-limiting step at high adhesiveness Palecek et al. [1998]. However, as adhesions are the elements that bear the contractile load, and break when overloaded, their number might matter. It controls how the load is shared and how readily anchors can rupture. We modified the model to limit the total number of anchors. Once the reservoir of anchors is consumed, new anchors can be added only at the same rate of detachment. The reviewer's intuition was correct, and as anticipated, with this additional rule growing phenotypes stabilize, widening the region of parameter space in which cells polarize.

The mechanism is the following. The growth phenotypes, and the way a finite adhesion pool suppresses them, result from the balance between contraction and effective adhesion. In the strong-adhesion regime the anchors are not overloaded, so contraction cannot retract the network and the continual addition of material at the edge can only expand the cell, producing growth. This is a natural "null" behavior of unbounded growth. Isotropic and anisotropic growth differ only in symmetry: as a growing cell enlarges, the contractile tension it carries increases, and beyond a certain size the symmetric, disk-like shape becomes unstable to elongation. A small asymmetry in the network is amplified and the cell extends along a preferred axis. Anisotropic growth is therefore a partially polarized state. Adhesion is weak enough to contract the added material but still too strong for a rear to detach and translocate the cell. In this regime the system lies between the isotropic growth and migration regimes. With an unconstrained adhesion supply this never resolves, because new anchors are added at the edge as the cell grows, holding the adhesion density, and hence the force per anchor, roughly constant, so nothing ruptures and growth continues without bound. A finite pool breaks the loop: with a fixed number of anchors, growth spreads the anchors over a larger area while the total contractile force keeps rising with the amount of material, so the force sustained by each anchor increases with cell size. Once that per-anchor force crosses the rupture threshold the anchors begin to fail, the network retracts and growth halts; equivalently, a finite pool makes the effective adhesion strength decrease as the cell grows, so a cell that begins

in the growth regime slides into the migration regime. This is the negative feedback that bounds the growing phenotypes and enlarges the migratory region: parameter values that gave unbounded growth in the open system now yield bounded migration. Simulations with a finite anchor pool confirm this: both the isotropic and anisotropic growth phenotypes polarize and migrate. In the anisotropic case the axis is already selected, and the finite pool supplies the rear rupture that lets the elongated cell detach and move; in the isotropic case the size-driven rupture breaks the symmetry of the growing disk.

In summary, a limited resource is not required for polarization; the unconstrained dynamics leads to self-polarization and emergent turnover of material, while a finite adhesion pool may help, enlarging the motile region (Figure S10). We thank the reviewer for prompting an analysis that, we believe, strengthens the manuscript's message in exactly the way suggested.

7. p18, l375 "contraction at the trailing edge not only balances the addition of new network but leads to a steady translocation of the whole system" Here, and also later on p28 l512 when UCSP model is mentioned, the authors may consider to cite some continuum models that also show that contraction at the rear is sufficient for a bifurcation to motility, especially Recho P, et al Phys. Rev. Lett. 2013, J. Mech. Phys. Solids 2015 and its generalization to adhesion, V Wössner, et al, New Journal of Physics 2024

   We thank the reviewer for these references. We added the suggested references in the Discussion.

8. As already stated above, I like the transverse actomyosin bundle very much. The authors may add a figure with a quantification of this state, when they discuss that "these structures are reminiscent of stress fibers" on p19 l405.

   We thank the reviewer for this suggestion. We have added a supplementary figure (Supp. Fig. S9) to quantify these structures. In brief, we computed the longitudinal and transverse density with respect to the direction of motion. Moving cells show more asymmetric longitudinal profiles (individual and averaged) compared to other phenotypes.

9. I like the idea to also study external cues that can bias migration. But at this point I find the introduction of a directional gradient in the ARTF ratio a bit too oversimplified/phenomenological. For e.g. durotaxis, can one not just implement a gradient in stiffness of the substrate springs or something similar? The authors should try to be more realistic here or at least discuss a bit more thoroughly how such changes of "real" parameters affect the ARTF.

We thank the reviewer for this comment. It was a mistake to describe the introduction of the gradient of ARTF ratio as a way to model specifically durotaxis. Instead, what we model here is the cell's response to an external gradient through modification of the strength of its substrate adhesions. An external gradient may be of substrate rigidity as in durotaxis or of the concentration of substrate ligand as in haptotaxis, and the resulting reinforcement of the adhesions up the gradient may be due to signaling rather than a direct mechanical response. In the model, we are in a general case of a rigid substrate: substrate rigidity is orders of magnitude higher than that of the network. Consequently, direct introduction of the gradient of substrate rigidity would not have influenced the network behavior. Nevertheless, the cell could sense increases in rigidity, even in rigid substrates, through signaling mechanisms and respond by reinforcing its adhesions. The text of the paper is modified accordingly.

10. In Fig3 and 4 the network displacement should be discussed/quantified more. I see large displacements in eg Fig3F that seem not so much correlated with the dipoles/forces in Fig3D, or maybe this is not so visible there. Can one quantify/correlate better?

    We agree with the reviewer: there is an apparent mismatch, but there is a mechanistic explanation for it. The flow maps (panels F/L) show the displacement produced by one minimization, which is dominated not by where dipoles are densest but by where anchors have just been removed. Releasing a peripheral anchor lets the contracted network snap inward, producing the large displacements seen at the edge of the centripetal system in Fig. 3F. The dipole map in panel D does not capture the force build-up and release that drives the flow.

11. In the results, the ratio of anchor removal threshold to dipole force (ARTF) is discussed quite a bit but in the discussion the authors state "The timescale of detachment effectively defines the behavior of the system". I suggest a discussion of ARTF is also needed in the discussion/conclusion

    We thank the reviewer for this comment. We have clarified in the Discussion that the key quantity governing the system dynamics is the anchor lifetime, which itself depends on the ARTF ratio. Accordingly, in the Results section, we place greater emphasis on the ARTF ratio, whereas in the Discussion we focus on the anchor lifetime.

2 Reviewer #2

The paper by Messi and colleagues investigates cell polarization and migration. To do so, they construct a model of the cellular machinery used for migration. They include filaments (modeled based on actin filaments), force dipoles (myosin), and anchors (focal adhesions). The key behavior they encode in their model is force-dependent breakage of the anchors. They investigate the

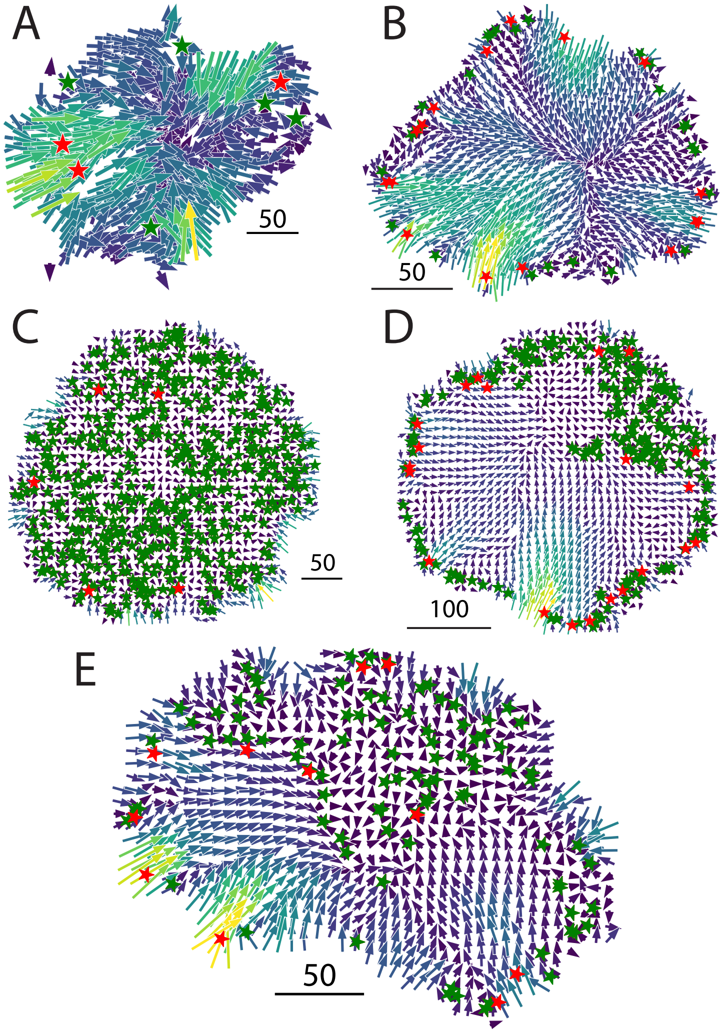

Figure 2: Flow in the cell with anchors and removed anchors for the different phenotypes. Network displacement during the minimization shown with arrows color-coded for the norm of the displacement. Anchors during minimization are shown in green. Anchors removed at the previous iteration are shown in red. A) Erratic, B) Centripetal, C) Isotropic, D) Anisotropic, E) Migrating

behavior of the model over ranges of parameter values that lead to different qualitative behaviors of the simulated cell.

Messi et al. find that in one extreme, when contractile forces are lower than the breaking strength of the adhesions, the system grows approximately isotropically. In the opposite extreme, when breaking strength is very low, adhesions do not persist and cells are also unable to move. Interestingly, in the middle regime, they find that the cell self-organizes into a polar, motile state. It is notable that no guiding cues or initial broken symmetry is necessary to create these states. These results are well presented and explained. I find these results interesting and valuable. I would note, however, that I am not an expert in the field of polarity establishment and motility, so am not well positioned to asses the novelty of this work within the context of similar models.

The model itself is of many movable nodes which can be connected by an energy function approximating the energy of a flexible filament. They can, in addition, be connected by an energy function approximating force generation by myosin. Finally, they can be attached elastically to a point in space, approximating adhesion to a substrate. Node positions for a given set of energetic links are determined by energy minimization. The links are then updated based on some rules, and energy is minimized again to model time evolution of the system. The energies used are reasonable, as is the procedure to simulate time through successive minimizations. The update rules, however, present a major issue with this work. Specifically, the update rules around removal of filaments and myosin links are presented as numerical concerns, but in my opinion require more careful treatment, as they encode key aspects of the biophysics of the model.

2.1 Major Comments

1. The update rules specify that nodes which are close together are merged, then bonds removed if they number above 10 (as well as myosin links). Another rule specifies that bent links are removed when the bend is larger than some angle. These update rules appear to hide the biophysics of actin disassembly. The model should be reformulated to include a biophysical description of this process, and the behavior of the model checked across some range of the associated parameters.

   We agree with the reviewer and have modified the text accordingly to present a biophysical description of the pruning/merging rules. Those rules are introduced in the model to keep the discrete network well behaved, but are motivated by identifiable features of actin-network disassembly. Pruning/merging is of two kinds:

   (i) Angle-based removal: The rule that removes a sharply bent hinge mimics the process in which an actin filament buckles and is severed once its radius of curvature becomes small enough under myosin contraction. Buckled actin severs at radii of a few dozen nanometers Cameron et al. [2001] to a few hundred nanometers Arai

et al. [1999], Murrell and Gardel [2012]. Our rule to remove bent hinges reproduces this severing: the bending energy fixes the local radius of curvature as $R = \ell_0/[2\sin(\phi/2)]$, with ϕ the deflection from straight. Thus the interior threshold angle α_{rem} corresponds to a severing radius ~ 80 nm, in the range of the values reported in Cameron et al. [2001], Arai et al. [1999], Murrell and Gardel [2012].

(ii) Density cap and node merging: Filaments are represented as one-dimensional bonds with no thickness, so by construction the network has no excluded volume and nothing bounds how many contractions can draw through a point; the per-node cap on bonds and myosin links, with the merging of nodes within d_{merge}, supplies the maximum local density that the filament diameter imposes in a real fiber network. Because the cap acts in the dense, compacted regions where myosin fragments and severs F-actin Haviv et al. [2008], Vogel et al. [2013], the material it removes represents myosin-driven disassembly. This rule is an *effective* representation of these processes, keyed on local density.

We investigated the robustness of the model with respect to both parameters.
Varying either the range of maximal number of dipoles or the interior threshold angle did not qualitatively change the behavior of the system. Decreasing the maximal number of dipoles reduced the parameter domain for migratory behavior, while varying the threshold angle had a biphasic effect on the parameter range for motion. Nevertheless, in all cases the system remained capable of spontaneous polarization and persistent migration.
We varied the maximum number of dipoles allowed between a pair of nodes while maintaining the maximum number of allowed bonds per pair of nodes fixed to probe the effect of their relative abundance. We considered systems with the maximum number of dipoles allowed between a pair of nodes ranging from 1 to 20. Reducing this limit caused the system to transition more rapidly to the growing phenotypes, but did not qualitatively alter the behavior of the model. Across the entire range explored, the systems remain capable of spontaneous polarization and persistent migration.
These results are now summarized in Supplementary Figure S11.

- The model has constant addition of material. The authors show that some systems converge to a constant cell area. The analysis of these systems is a key point of the paper. Do these systems also reach a steady state number of actin and myosin bonds? The answer to this question should be shown. In the case that they do, it would imply that the rate of actin loss by disassembly is equal to that of actin addition, making the two terms of equal importance to the behavior of the model. In the case that a steady state is not reached, this

would imply that these systems are getting denser over time. In this case, the authors would need either to argue why studying such a system at early times is relevant, or add further disassembly rules to enable steady state density regimes.

We thank the reviewer for this remark. In the revised version of the manuscript, we have added a supplementary figure demonstrating that the time evolution of the different network components parallels the evolution of the cell area. Specifically, once the cell area reaches a steady state, the other components of the network also attain steady-state behavior (Figure S7).

- From my reading of the model, the update rules basically encode actin disassembly which happens only at high concentrations and actin disassembly which happens when actin is compressed. What are their relative contributions to the total? Are actomyosin systems known to behave in this way? The biophysical model of disassembly should be put in context of what is known.

  In the revised version of the manuscript, we added supplementary figures (Figures S2 and S3) showing the time evolution of the relative contributions of disassembly due to concentration and compression. The features of actin-network disassembly have been discussed at the beginning of this response.

2.2 Minor Comments

1. Equation 2 gives the energy of bending as a function of bend angle, with a $\sin^2$ dependence. In the proceeding paragraph, there is a description of the bending energy of a rod with constant curvature. I was not able to see how the $\sin^2$ term emerges from a constant curvature bend. The authors should briefly show how they arrive at this term.

   The original manuscript did not explicitly state two assumptions underlying the derivation. The bending energy scales as $\sim \frac{1}{R^2}$. The osculating circle is a circle that best approximates the curvature; in our discrete model, the radius of this circle is the circumradius (see the figure and Supp. Fig. S1):

$$R = \frac{\ell_0}{2\sin\left(\frac{\phi}{2}\right)}$$

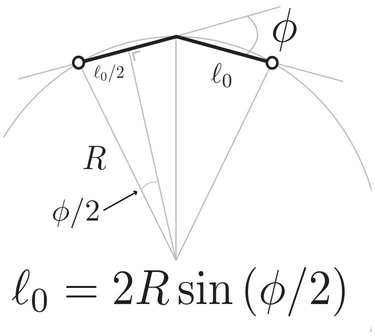

2. In the model description, bonds are described as being able to be compressed, but also bent. I see no description for when compression is used vs. bending, or when bends are created.

   We agree that the description of the different bond deformation modes was not sufficiently clear in the original manuscript.
   In our model, as in Ronceray et al. [2016], Messi et al. [2020], stretching/compression and bending are simply two independent modes of deformation (i.e., two degrees of freedom) that both contribute to the Hamiltonian. The model does not prescribe when a bond should stretch/compress or bend. Instead, both deformation modes are allowed to relax toward mechanical equilibrium.

3. It is strange to me that "consecutive bonds that are initially aligned can also form hinges". Does this model something like a distribution of filament lengths? This choice would benefit from some explanation.

We thank the reviewer for raising this point. We did not model a distribution of filament lengths. The confusion comes from the fact that the term hinge was not clearly defined in the original manuscript. In our model, a hinge refers to any element that carries a bending energy, either a single bond that bends at its midpoint or a pair of consecutive, initially aligned bonds that bend relative to one another. We have clarified this terminology in the revised Methods section.

4. The meaning of point 2 in the pruning section is unclear. I gather from the later description that it is intended to mean the filament is removed from the system, but from the description in this point it is unclear what a "lower" angle means. Are hinges ever converted back to filaments?

   We thank the reviewer for raising this point. We have modified the description of the different processes of network remodeling. The angle-based removal aims to mimic the severing of an actin filament due to buckling once its radius of curvature is small enough. Since the elements are discrete, we computed the interior angle of a hinge, rather than the curvature, and when this angle is lower than the removal angle, the hinge is removed from the system.

5. Table 1 should be expanded to include references for the values, where applicable

   We have split Table 1 into a table dedicated to energetic parameters and another table for remodeling parameters. Values and ranges of values are discussed in a specific section of the Supplementary Information.

6. The algorithm for placement of filaments, myosin links and anchors is not described

   We have modified the model description to include the initialization of the network. The network is initialized as follows:

   (a) *Node creation:* Nodes are placed on a hexagonal lattice (coordination number six), centered at the origin and restricted to a disk of radius 40 (in model units).

   (b) *Bond creation:* Each edge of the fully coordinated lattice is independently retained with probability $\rho = 0.7$. Since this is well above the lattice bond-percolation threshold ($p_c \approx 0.35$), a spanning component always exists: we keep the largest connected component and discard all smaller components and isolated nodes.

   (c) *Anchor creation:* Each node of the remaining network is independently made an anchor with probability ρ_a.

   (d) *Dipole creation:* Finally, we place $N_d = \rho_d N_{\text{node}}$ dipoles, where N_{node} is the number of nodes in the remaining network. Each dipole

is assigned to a pair of lattice-adjacent nodes drawn at random; a pair may be chosen even if its bond was removed during dilution, and the same pair may receive more than one dipole.

7. The threshold of 10^5 units of cell area is chosen as a limit for a "stable" cell size. In figure 1, the cells which are going above this limit are clearly already slowing down in their area growth. Why was this value chosen, and is it meaningful? The authors might consider the relationship of the cell growth rate with the rate of area addition instead.

   The threshold area is a practical cutoff that halts unbounded growing cells at finite computational cost. We should, however, provide a clearer reading of the figure: the cells above the threshold are not slowing. Their area is plotted on a logarithmic axis against linear time, on which a power law appears to slow down. On linear axes the same curves are parabolic; the area grows quadratically according to the rule of addition of material. These cells are growing without saturating, so the 10^5 value truncates runs that would otherwise grow without bound.

   In physical units, using $\ell_0 \approx 150\,\text{nm}$, the cutoff corresponds to an area $\approx 2 \times 10^3\ \mu\text{m}^2$, that is to say a typical cell size of 50 μm across; we believe it is a meaningful cutoff for motile-cell size.

   According to the reviewer's suggestion, we have added a supplementary figure (Supp. Fig. S6) showing the time evolution of the cell growth rate and the rate of area addition.

8. The results of the paper would be strengthened by showing the hypothesis stated on line 357: that there exists a negative feedback between adhesion and contraction. The probability of nearby anchors to fail in the next iteration / time step could be compared to those far away.

   We thank the reviewer for this suggestion. We have carried out this analysis and found that, across phenotypes, anchors closer to a recently removed anchor are more likely to fail in the next step than distant ones, accompanied by a rise in their force as the removed anchor's load redistributes onto its neighbors. These new results thus support the proposed feedback between adhesion and contraction. These results are shown in a new supplementary Figure S8, and the corresponding methodology is described in Supplementary Information.

9. In the text, it is described that "the measurement of anchor lifetime appears to reach a finite plateau. This is an artifact...". I was not able to understand which feature of the data this line describes. The authors should perhaps reconsider how they include the anchors which never break into the measure of lifetime.

The sentence refers to the anchor-lifetime measurements as a function of the ARTF ratio in Fig. 6A; we have revised the manuscript to explain it more clearly. Anchor lifetime is measured as the number of iterations during which an anchor exists. If an anchor is still present when the simulation ends, its lifetime is truncated at the end of the run rather than being discarded or treated as infinite. Consequently, when the true lifetime becomes comparable to or exceeds the simulation duration, for very stable anchors in rapidly growing networks, the measured mean lifetime is systematically underestimated (an effect compounded by anchors created late in the run, which have little remaining time). The plateau in Fig. 6A is therefore a censored lower bound, not an intrinsic maximum lifetime. This underestimate does not affect our conclusions: all systems in the plateau region (lifetimes above ≈ 15 iterations) correspond to the isotropically growing phenotype, where cells reach their limit area at a similar number of iterations, and further increases in anchor lifetime produce no change in the dynamics. We now state in the text and the Fig. 6A caption that plateau values are lower bounds, while lifetimes below the plateau are genuine, turnover-limited measurements.

10. The way the paper approaches the relationship of the simulation with time needs to be consolidated. In figure 1, "iterations" is used to refer to what in other places is referred to as time or timestep. In the conclusion, a formal equivalence with time is stated and the timestep approximated in seconds. Consistent terminology should be used. It should also be clarified how the timescales of the various processes compare. Should, for example, we think of the mechanical equilibrium as fast compared to the chemical kinetics, which set the time evolution? Is mechanical equilibrium slow comparatively, dominating the timestep? In the discussion the protrusion rate is used to approximate the timestep, why is this the correct measure to pin the time evolution to instead of e.g. turnover or mechanics?

    We thank the reviewer for the comment. In the revised manuscript and figures, we now use a consistent terminology: iterations instead of time and timesteps.

    The model is by design quasi-static. Within each iteration, the network is relaxed to mechanical equilibrium by energy minimization, and the chemical and structural events are applied only to the relaxed configuration. This rests on a separation of timescales: actomyosin reaches mechanical equilibrium rapidly while chemical kinetics dictate the slower time evolution Deng et al. [2006]. The network is always at mechanical equilibrium when these slow events are applied. In this sense the fast mechanical variable is adiabatically slaved to the slow chemical and structural state, including protrusion, which is what drives the physical time evolution.

    The reason why we used the protrusion rate is that, among the slow processes that drive the dynamics, protrusion is the only one that advances

the system by a fixed geometric increment per iteration, identical across all simulations. The other processes cannot serve as a clock. Mechanical relaxation is fast and adiabatically eliminated, and carries no physical duration. Adhesion turnover and filament severing are triggered by force thresholds, local geometry, and density rather than by an input rate: they occur as a result of the relaxed mechanics, so their number of events per iteration is an outcome of the dynamics, not a fixed increment against which time could be calibrated. Protrusion is thus not an arbitrary choice but the only process carrying a constant, model-defined step, which is why we use it to convert iterations to physical time.

11. The final result is that a spatial bias in the ARTF ratio is sufficient to polarize the cell. In the Methods I see this is implemented by changing the breaking threshold of the anchors. Is there a biological reason to think this might be the case in some form of cell migration? How might an external signal lead to a changing breaking threshold? What molecules might be involved? The discussion should be expanded.

    We thank the reviewer for this comment. In the model, we simulate the cell's response to an external cue through a spatial modulation of the anchor removal threshold, corresponding to a variation in the strength of cell–substrate adhesions. This choice is not intended to represent a specific molecular mechanism, but rather a generic cellular response to an external signal.
    Biologically, modulation of adhesion strength can arise through signaling pathways that regulate focal adhesion assembly, maturation, or disassembly Parsons et al. [2010]. Such pathways are known to be influenced by external cues, including gradients in extracellular matrix composition (haptotaxis) King et al. [2016] and substrate stiffness (durotaxis) Espina et al. [2022]. In our model, these complex signaling processes are coarse-grained into a single parameter controlling the effective strength of cell–substrate adhesions.
    We have expanded the paragraph on biased migration to clarify the biological interpretation of this modeling choice in the revised manuscript.

12. The submitted draft of the manuscript has clearly not been sufficiently proofread (e.g. "(other refs.?)" in line 102, numerous typos and leftover sentence fragments in the Methods section). This reviewer would point out that such sloppiness makes it doubly difficult to decipher the author's intent and would ask the professional courtesy of the authors to do this work before asking others to evaluate the paper.

    We thank the reviewer for this comment. We carefully proofread the revised manuscript.

2.3 Notes on writing and presentation

1. In equations, variable subscripts etc. which are full words should be printed in plain text, not math italics (can be accomplished using LaTeX in the equation environment). Care should be taken to do this consistently throughout the text (e.g. they are mixed in the equation describing the persistency score)

   We thank the reviewer for pointing this out. This has been corrected throughout the manuscript.

2. Equation 1 assumes uniform bending, but this assumption is only introduced after the equation

   We thank the reviewer for this comment. The issue has been resolved in the revised version. Most of the model derivation is now in the Supplementary Information, and the assumption of uniform bending is mentioned before the equation.

3. In the title block, affiliation 4 is unused

   We thank the reviewer for pointing this out. The unused affiliation has been removed.

4. There are too many typos in the Methods section to list here, it needs to be gone through thoroughly

   We thank the reviewer for pointing this out. We have thoroughly revised the Methods section, paying particular attention to clarity, wording, and typographical errors.

5. The first sentence of the author summary is awkwardly phrased and should be rewritten

   According to the suggestion of the reviewer, we have rephrased the first sentence of the Author Summary.

6. Equation 8 overruns the page

   We thank the reviewer for pointing this out. The equation has been reformatted so that it no longer extends beyond the page margins.

7. In line 248, the anchors are described as "fixed-position nodes" but this contradicts the methods

   We thank the reviewer for pointing this out. The revised manuscript now consistently describes anchors as nodes attached to fixed springs with zero rest length.

8. Line 340: "is uniform and isotropic" should include an "approximately"

According to the suggestion of the reviewer, we added "approximately" in the revised version.

9. In figure 2 C, the authors present a schematic about trajectory reconstruction. The relationship of this to the message of the figure is unclear from the figure and legend. From the rest of the paper, I understand this to be connected to the construction of S_{pers}. In the current version of the figure, the placement makes it look like actual and reconstructed are related to the figures below, which I gather is not true. Panels c, d, and e should be rethought to better allow the reader to understand the message of the figure.

   We thank the reviewer for this comment. We have restructured Figure 2. The trajectory-reconstruction schematic (former panel C) and the individual MSD panels have been moved to the Supplementary Information; this removes the misleading spatial adjacency that made "actual" and "reconstructed" appear connected to the panels below, which was not intended.

10. In the panels showing circular kimographs, a different color combo from green and red should be chosen, as red-green colorblindness is fairly common.

    We thank the reviewer for this suggestion. We have changed the color scheme from red–green to magenta–green.

11. Typos:

    a) Line 8: "membrane ,"
    b) Line 31: "description"
    c) Line 40: "we extend discrete elastic model"
    d) Line 41: "to dynamical cases"
    e) Line 386: missing parenthesis
    f) Line 513: unnecessary space

    We thank the reviewer for pointing out these typographical errors. We have carefully proofread the revised version of the manuscript.

3 Reviewer #3

Summary of Article contributions: The authors extend a computational discrete active network model – representing cellular actomyosin networks – to the case of dynamic adhesions, exploring how symmetry breaking in and persistent migration of elastic cytoskeletal networks can originate purely through contraction-adhesion dynamics. The computational cell model incorporates a simple rule: anchors (representing adhesions) break under a threshold force. The authors observe five distinct phenotypes emerging as a function of anchor

density and the ratio of anchor removal threshold to dipole force magnitude (ARTF): small networks moving erratically, large networks with centripetal contraction, migrating networks, networks growing isotropically, and networks growing anisotropically. Importantly, the migrating phenotype, which occurs at intermediate ARTF ratios, features characteristics associated with persistently migrating cells: e.g., network shape, the localization of force dipoles in the rear, and the localization of anchors in the front. The authors illuminate that the significance of the ARTF ratios lies in its impact on anchor lifetime, as the ARTF ratio defines the number of dipoles required to break an anchor. As such, the emergence of a migrating phenotype at intermediate ARTF suggests an optimal anchor lifetime. In further evidence of the significance of the balance of contraction and adhesion for network dynamics, the authors demonstrate that the memory for polarization, and thus directionality, can only be lost by perturbing both the dipole and anchor distributions in the network. Finally, the authors demonstrate that the model can recapitulate symmetry breaking due to adhesion-associated migration cues (haptotaxis / durotaxis) by introducing a gradient of the ARTF ratio, resulting in highly directional and persistent migration for both the migrating phenotype and erratic phenotype. In all, this work highlights that polarization can be an emergent behavior of cytoskeletal-like networks due to mechanics alone.

Summary of Reviewer impression: This work appears to be novel. The computational approach employed by the authors is well motivated, as it contributes to the broader understanding of how cytoskeletal mechanics contributes to cell polarity and migration by isolating the contributions of actin, myosin, and adhesion from in vivo complexity (signaling, upstream regulation, biomechanical feedback, etc.). The authors extend a previous discrete active network model, yielding biologically relevant conclusions regarding the important balance of adhesion and contraction dynamics in cells. As such, the results of this manuscript would certainly be of interest to the readership of PLOS Computational Biology. However, the authors need to clarify model and computational details and, importantly, distinguish novel contributions from those of prior work. My comments are as follows

3.1 Major Comments

1. In the computational discrete active network model, it is essential that the authors clarify and emphasize this work's particular novel contributions. This model (largely the same as the author's prior work in [2]) is an extension of the model in reference [32] (itself building off [36]), as noted in Lines 40-43 and 66-68. However, the language in this manuscript suggests that the model development occurred in this project (e.g., in the Author Summary, "we developed a computational model of a simplified cell made only of elastic connections, contractile forces, and attachment points to its surroundings"). This is also the impression given by the Methods section, where the computational cell model is rederived despite already

largely being described in [2], [32], and [36]. I am not convinced that the extensive model description in the Methods section adds value to this manuscript – if the authors want to keep this for completeness, aspects which are genuinely novel should be emphasized over aspects which are the same as [2], [32], or [36]. It would also be helpful for the authors to clarify how the model was used in [2] and [32] (which would strengthen the argument for its usefulness in describing mechanical actomyosin dynamics in cells).

We thank the reviewer for this comment. We have entirely revised the model description in the Methods section. In particular, we clarified what is inherited from Ronceray et al. [2016], Messi et al. [2020] and what is novel in the present work: the remodeling rules and the dynamics. Details of the normalization of the Hamiltonian and the parameters are now provided in the Supplementary Information.

2. The description of the model in the Methods section is difficult to understand due to a lack of clarity in terminology which must be addressed:

    a) The words "bond", "segment", and "filament" seem to be used interchangeably or are simply poorly defined. For example, in Lines 81-83, segments are noted as inextensible but filaments are allowed to stretch – this seems contradictory to the note in Line 68 about filaments being composed of chains of inextensible segments. This issue probably also relates to a confusion regarding differences between this work and prior work in [36] (relating to major revision comment #1). This confusion also arises in the discussion of rest length, where bonds have rest length $2\ell_0$ (Line 71), segments have rest length ℓ_0 (Line 84), and filaments have rest length ℓ_p (Line 102) which is also persistence length (Line 69) – I suspect this last one is a typo, but of course adds to confusion.

    We thank the reviewer for this comment. The description of the model has been extensively rewritten to clarify the confusing points. In particular, the term "filament" is now used exclusively to refer to actin filaments in the biological discussion, whereas the terms "bond" and "segment" refer exclusively to the model. We have made it explicit that bonds are made of two straight segments attached at nodes on a hexagonal lattice. We also clarify the difference between ℓ_0 and ℓ_p: the former is the segment rest length whereas the latter is the filament persistence length used to compute the bending and spring energies of the bonds. We also clarified the differences between the present work and the studies of Ronceray et al. [2016] and Messi et al. [2020].

    b) Similarly to above, it is unclear what the difference in energy contribution is between filaments which are bent (eq. 2) and "hinges"

(Line 77), especially because the full Hamiltonian in eq. 14 only incorporates “hinges”. Presumably filaments are composed of several segments which all form hinges, but this is not clearly stated.

We thank the reviewer for this comment. In the revised description of the model, we clarified that hinges are either the midpoints of bonds or contact points between bonds that are aligned in the initial network configuration. The term ”filament” has been removed from the model description for clarity. Consequently, in the computation of the Hamiltonian, the bending energy is summed over hinges, not over filaments.

c) In Line 102, is the length of myosin bipolar minifilaments defining bond/segment rest length ℓ_0? If so, this should be clarified.

The reviewer is right: we use the length of myosin minifilaments to convert length from model units to physical units. This is now clarified in the Supplementary Information.

d) A minor related comment: Is the range of dipole force magnitudes listed in Line 109 in pN units or in nondimensional model units?

The dipole force magnitude is dimensionless. We have modified the parameter table to indicate explicitly which parameters are dimensionless.

3. Pruning of the hinges, nodes, and bonds can in principle result in effective turnover of the actin network, which would be a nontrivial contribution to the dynamics. The authors need to report how much pruning of the network (removal of hinges, merging of nodes, rearranging and removal of bonds) happens over the course of simulations for different conditions. I suspect that highly migratory cases (particularly those which stay below the area threshold) may have high rates of network pruning that would correspond to non-negligible levels of network turnover (e.g., it seems true in Movie 1 where the network density does not appear to change with time, and also in Movie 10 after the half-way point). Further, the authors should then compare the rate of pruning to in vivo rates of actin turnover.

We thank the reviewer. We have added two supplementary figures that report the relative contributions of the disassembly mechanisms and their evolution over the course of the simulations for different values of the ARTF ratio (Supplementary Figures S2 and S3). Consistent with the reviewer’s expectation, the motile phenotypes, both erratic and migrating, including the cases below the area threshold, show higher pruning rates than the growing phenotypes.
To compare with physiological turnover, we note a structural feature of the model. New material is incorporated only at the periphery, whereas disassembly acts within the bulk. As a consequence, in a migrating cell, the network is continuously renewed from the front, while the trailing material is pruned away, so that by the time the cell has translocated by

approximately its own size, essentially its entire network has been replaced. In keratocytes, the actin network turns over on a timescale of seconds across a front-to-rear distance Raz-Ben Aroush et al. [2017], Theriot and Mitchison [1991].

4. In Lines 292-301, the authors note that large cells exhibit low persistence scores, but they appear more persistent because of a subtlety regarding actual displacement in comparison to cell size. As such, Figure 2D is incredibly misleading, where several large cells exhibit high persistence according to the chosen measure. Moreover, since the simulation time for large cells is significantly shorter than for small cells, it is not clear to me that their persistence is comparable in the manner presented in this work.

   We agree with the reviewer that, as described in the original manuscript, the discussion of the persistence score was potentially misleading. In the revised version, we now state clearly that large cells with high scores are not migrating in the usual sense: their net displacement is small relative to their size and is dominated by directed, growth-driven translocation of the centroid rather than by locomotion. We have also renamed the quantity for clarity: it is now presented as the straightness of the normalized trajectory (SNT), rather than a "persistence" score. Two important points:

   (a) It is computed from unit direction vectors between successive positions of the cell center, so neither the absolute displacement nor the cell size enters the calculation. SNT measures the shape (straightness) of the trajectory, not the distance traveled. A large cell can therefore have a high SNT together with a small net displacement, which is the growth-driven translocation described above.

   (b) In the submitted version, S_{pers} was initially computed from the MSD with a variable maximal lag that depended on the duration of the simulation. We compared the values of S_{pers} obtained with variable maximum lag and with fixed maximum lag (40 iterations). The Pearson and Spearman correlations are 0.91 and 0.90, respectively, thus indicating that the measure of persistence (now straightness of normalized trajectories) remains unchanged when the maximum lag is similar to the simulation time of the fast-growing cells.

   In the revised Figure 2, we removed the colormap encoding simulation time, which was confusing.

   a) To at least clarify Fig 2D, the authors could consider adding the cell boundary outline at the end of trajectories in Fig 2E (if this change makes the figure too large, Fig. 2E could become a supplementary figure).

      We thank the reviewer for this comment. We have modified Figure 2E (now Figure 2D) to show the typical cell size along with the trajectories.

b) Otherwise, in discussing the difference in persistence of large versus small cells, the authors should address how this comparison (and its usefulness) is impacted by the difference in simulation time necessitated by the unbounded growth inherently defining large cells.

   As previously discussed, the persistence score (now SNT) is robust to simulations with different durations.

c) Relatedly, perhaps a useful plot would be persistence score vs ARTF ratio, given the high correlation shown in Fig 2B and then explored in Figs 3-5. I suggest the authors add this to Fig 2, unless it would be misleading in a manner similar to Fig 2D.

   We agree with the reviewer that there is a high correlation with ARTF. However, the aim of Figure 2 is to illustrate the difference in persistence among large cells. Showing the persistence score as a function of ARTF would make this observation less straightforward. For this reason, we believe that keeping persistence vs ranked area provides a more straightforward message.

5. For Figures 3-5 as well as Movies 1-10, the authors should report which parameters are used to generate the displayed characteristic features/movements (specifically, the parameters which distinguish between the total 150 parameter sets explored).

   The parameters for every simulation of every movie and every figure can be found in a csv file on the data server that was published with the original submission. We added the parameters for the movies and the figures in their respective captions.

3.2 Minor Comments

1. There are several typos throughout the manuscript which should be addressed:

   We thank the reviewer for their careful reading. Points i to viii have been corrected in the revised manuscript.

   i. Lines 28-29: "[A] Discrete elastic model of actin-myosin network investigated the effect [of] nonlinear actin elasticity on force transmission...."

   ii. Lines 30-31: "To investigate polarization, other studies [described the] actin-myosin network as..."

   iii. Lines 38-39: missing ")"

   iv. Line 102: "(other refs?)"

   v. Line 167: "we considered a restarted the simulation"

   vi. Line 196: "Network flowwas"

vii. Line 296-297: “small cells exhibiting either erratic or persistent motion, expanding but stationary cells, and larger...”

viii. Line 386: missing “)”

2. In this work, the authors constrain their focus to lamellipodium-based mesenchymal migration. However, the manuscript seems to imply that this is the only (relevant) migration mode, neglecting bleb-based (amoeboid) migration which is prevalent in 3D microenvironments. The authors should clarify this focus on actin protrusion-based migration somewhere in the introduction.

   We thank the reviewer for this comment. In the revised Introduction, we clarify that we consider adherent mesenchymal cells.

3. The elastic modulus of the substrate is chosen to be 1e6 (“very high”, Line 110). The authors should clarify why they choose this order of magnitude, and whether the results would change qualitatively at lower substrate elastic moduli.

   We chose a large anchor spring constant in order to represent the rigid-substrate limit of the model. In our units, $\mu_s = 10^6$ corresponds to $10^5\,\mathrm{pN\,nm^{-1}}$, i.e. about three orders of magnitude larger than the bond spring constant, so that substrate deformations remain negligible compared with network deformations. To connect this value to physical units, we use a coarse-grained estimate $\mu_s \sim EA/L$, where A is an effective adhesion area and L is an effective deformation length scale. Taking A to be of the order of a nascent adhesion, which is about $10^{-2}\,\mu\mathrm{m}^2$ Changede et al. [2015], and L to be of the order of the anchor displacement before failure ($L \sim 0.1\ell_0$) gives an estimated substrate modulus in the range 10^6–10^7 Pa, qualitatively consistent with a stiff substrate. Experimental studies on keratocytes further show that substrate stiffness can significantly affect migration behavior: softer substrates reduce migration speed and directional persistence in fish keratocytes Dziob et al. [2016], while matrix rigidity also regulates keratocyte morphology, polarization, and persistence of fan-shaped motile states Riaz et al. [2016]. Exploring lower substrate stiffness in the model would therefore be an interesting extension, but is beyond the scope of the present study.

4. In parts of the Methods (Lines 100-106) and Discussion (Lines 518-527) sections, the authors note parameters and resultant scales in space and time which correspond to experimentally measured or physiologically reasonable values in units. I recommend the authors add a column to Table 1 which includes the resultant in-units values and references when those parameters are drawn from the literature. Regardless of if this is added to the table, exactly which parameters were selected based on experimental data versus previous modeling work (as noted in Lines 120- 122) should be noted explicitly by the authors.

We thank the reviewer for this comment. We have substantially revised the Methods section. In the revised manuscript, Supplementary Table S1 now includes the complete list of model parameters, values in model units, corresponding values in physical units and references (experimental measurements, previous modeling work, or assumptions made in this study).

5. In Lines 117-120, the authors note that parameters can be grouped by whether they are structural or related to turnover/pruning. This could be added to Table 1 as well, though not absolutely necessary.

   In the revised version of the manuscript, we have split Table 1, separating the parameters associated with the remodeling.

6. In Lines 263-264, the authors note "see Methods for details" regarding when simulations are stopped. This is never mentioned in the Methods section, so it should be either added by the authors or removed from Lines 263-264.

   We thank the reviewer for pointing this out. In the revised version, we have removed the mention of the Material and Methods section.

7. In Lines 123-125, the authors state which parameters are allowed to vary (and why) – it would be helpful to restate or reference this in Lines 268-269 regarding the ensemble of 150 distinct parameter sets used in the Results section. In addition, some results are averaged over identical parameter sets (as noted in the caption of Fig 2), but how many identical parameter sets are considered does seem to be stated – the authors should clarify this detail.

   We thank the reviewer for this comment. Typically, each set of parameters is replicated ten times. We now provide the number of replicates in the caption of Figure 2. In the revised version of the manuscript, we also specify which parameters are varied.

8. In Figure 2C, areas under MSD curves for ballistic, random, and simulation trajectories are shaded for large cells but not small cells. As the shading is not particularly useful and obscures the orange/blue simulation data, I recommend the authors remove the shading and just keep the dotted lines corresponding to ballistic and random MSD bounds. Also, the plots should be labelled as corresponding to large/small cells, instead of only being noted as such in the caption.

   We thank the reviewer for this comment. To improve the readability of the figure, we have now moved panel C to the Supplementary Information.

9. For the linear kymographs, how is the "line through the approximate center of the system" (Line 186-187) defined? Is it meant to correspond to the migration direction (if there is one)? Relatedly, how is angle =

0 chosen for radial kymographs? In principle, one would expect angle = 0 to correspond to the migration direction, but this does not seem true throughout Fig 5C.

We thank the reviewer for this comment. For the linear kymographs, a line was drawn by hand through the approximate center of the network. For phenotypes exhibiting a well-defined axis of growth or migration (i.e., migrating and anisotropic), this line was aligned with and in the same direction as that axis. For other phenotypes, the direction was chosen at random.
For the radial kymographs, the angular coordinate is defined in a fixed reference frame with respect to the substrate rather than relative to the migration direction. Consequently, the angular position 0 points to the right (as in a trigonometric circle) and does not necessarily coincide with the direction of motion. This choice was made intentionally, as it allows changes in the migration direction to be visualized directly in the kymographs.
The captions of those figures have been revised accordingly.

10. In Fig 6C, the red line = 1 defining anchor removal threshold should be noted as such in the figure caption.

    We thank the reviewer for pointing this out. We added a note to the figure and clarified in the caption that the red line at 1 indicates the anchor-removal threshold.

11. In Fig 6D, the effects of varying gradient strength are shown for migrating cells and erratic cells, but for migrating cells two different nonzero gradient strengths are used while for erratic cells only one nonzero gradient strength is used. The authors should clarify whether (and if so, why) the conditions in "on" vs "off" gradient cases are different for the two phenotypes. It would also be valuable to include an intermediate gradient condition for the erratic cells, to aid the comparison in Fig. 6D.

    We thank the reviewer for this comment. We have amended the figure and the manuscript to include intermediate gradient strengths.

12. In Lines 491-493, the authors comment on how their results show that the balance of contractility and adhesion impacts cell migration speed, but this is not actually analyzed in this manuscript so the authors should refrain from this being a conclusion of this work.

    We thank the reviewer for this comment. What we actually wanted to say is that experimental studies investigated the influence of contractility and adhesion on migration speed while our model uncovers the role of contraction-adhesion balance in polarization itself. The text was amended accordingly in the revised version of the manuscript.

13. There are other computational models exploring actomyosin mechanics questions which do not employ a viscoelastic continuum formulation, e.g., Cytosim, Maxian et al. 2021 (https://doi.org/10.1371/journal.pcbi.1009240), Kim et al. 2009 (https://doi.org/10.1371/journal.pcbi.1000439), and Rutkowski and Vavylonis 2021 (https://doi.org/10.1371/journal.pcbi.1009506). In the introduction, the authors should clarify the benefits and/or differences of their approach over these other types of non-continuum mechanical models of actomyosin networks, for addressing questions relating to polarization.

    We thank the reviewer for pointing this out. We clarified in the revised Introduction that our approach is complementary to other non-continuum mechanical models of actomyosin networks, which are particularly well suited to resolving filament-scale organization, motor activity, crosslinking, and network architecture.

References

Yasuharu Arai, Ryohei Yasuda, Ken Ichirou Akashi, Yoshie Harada, Hidetake Miyata, Jr. Kinosita, Kazuhiko, and Hiroyasu Itoh. Tying a molecular knot with optical tweezers. *Nature*, 399(6735):446–448, 1999. doi: 10.1038/20894. URL `https://doi.org/10.1038/20894`.

Daniel L. Barton, Silke Henkes, Cornelis J. Weijer, and Rastko Sknepnek. Active Vertex Model for cell-resolution description of epithelial tissue mechanics. *PLoS Computational Biology*, 13(6):e1005569, 2017. doi: 10.1371/journal.pcbi.1005569.

L. A. Cameron, T. M. Svitkina, D. Vignjevic, J. A. Theriot, and G. G. Borisy. Dendritic organization of actin comet tails. *Current Biology*, 11(2):130–135, 2001. ISSN 0960-9822. doi: 10.1016/S0960-9822(01)00022-7.

Rishita Changede, Xiaochun Xu, Felix Margadant, and Michael P. Sheetz. Nascent integrin adhesions form on all matrix rigidities after integrin activation. *Developmental Cell*, 35(5):614–621, 2015. ISSN 1534-5807. doi: 10.1016/j.devcel.2015.11.001.

Linhong Deng, Xavier Trepat, James P. Butler, Emil Millet, Kathleen G. Morgan, David A. Weitz, and Jeffrey J. Fredberg. Fast and slow dynamics of the cytoskeleton. *Nature Materials*, 5(8):636–640, 2006. doi: 10.1038/nmat1685. URL `https://doi.org/10.1038/nmat1685`.

Dominika Dziob, Tomasz Kołodziej, Jakub Nowak, Paweł Cyzio, Joanna Raczkowska, Jadwiga Laska, and Zenon Rajfur. Effect of substrate elasticity on macroscopic parameters of fish keratocyte migration. *Physical Biology*, 13 (5):054001, 2016. doi: 10.1088/1478-3975/13/5/054001.

Jaime A Espina, Cristian L Marchant, and Elias H Barriga. Durotaxis: the mechanical control of directed cell migration. *The FEBS journal*, 289(10): 2736–2754, 2022.

Alexander G. Fletcher, Miriam Osterfield, Ruth E. Baker, and Stanislav Y. Shvartsman. Vertex models of epithelial morphogenesis. *Biophysical Journal*, 106(11):2291–2304, 2014. doi: 10.1016/j.bpj.2013.11.4498.

Nathan D. Gallant, Kristin E. Michael, and Andrés J. García. Cell adhesion strengthening: contributions of adhesive area, integrin binding, and focal adhesion assembly. *Molecular Biology of the Cell*, 16(9):4329–4340, 2005. doi: 10.1091/mbc.e05-02-0170.

Lior Haviv, David Gillo, Frédéric Backouche, and Anne Bernheim-Groswasser. A cytoskeletal demolition worker: Myosin II acts as an actin depolymerization agent. *Journal of Molecular Biology*, 375(2):325–330, 2008. doi: 10.1016/j.jmb.2007.09.066.

Samantha J. King, Sreeja B. Asokan, Elizabeth M. Haynes, Seth P. Zimmerman, Jeremy D. Rotty, Jr Alb, James G., Alicia Tagliatela, Devon R. Blake, Irina P. Lebedeva, Daniel Marston, Heath E. Johnson, Maddy Parsons, Norman E. Sharpless, Brian Kuhlman, Jason M. Haugh, and James E. Bear. Lamellipodia are crucial for haptotactic sensing and response. *Journal of Cell Science*, 129 (12):2329–2342, 06 2016. ISSN 0021-9533. doi: 10.1242/jcs.184507. URL `https://doi.org/10.1242/jcs.184507`.

Angelika Manhart, Téa Aleksandra Icheva, Christophe Guerin, Tobias Klar, Rajaa Boujemaa-Paterski, Manuel Théry, Laurent Blanchoin, and Alex Mogilner. Quantitative regulation of the dynamic steady state of actin networks. *eLife*, 8:e42413, 2019. doi: 10.7554/eLife.42413.

Zeno Messi, Alicia Bornert, Franck Raynaud, and Alexander B. Verkhovsky. Traction forces control cell-edge dynamics and mediate distance sensitivity during cell polarization. *Current Biology*, 30(9):1762–1769.e5, 2020. ISSN 0960-9822. doi: 10.1016/j.cub.2020.02.078.

Michael P. Murrell and Margaret L. Gardel. F-actin buckling coordinates contractility and severing in a biomimetic actomyosin cortex. *Proceedings of the National Academy of Sciences*, 109(51):20820–20825, 2012. doi: 10.1073/pnas.1214753109.

Sean P. Palecek, Anna Huttenlocher, Alan F. Horwitz, and Douglas A. Lauffenburger. Physical and biochemical regulation of integrin release during rear detachment of migrating cells. *Journal of Cell Science*, 111(7):929–940, 1998. doi: 10.1242/jcs.111.7.929.

J Thomas Parsons, Alan Rick Horwitz, and Martin A Schwartz. Cell adhesion: integrating cytoskeletal dynamics and cellular tension. *Nat Rev Mol Cell Biol*, 11(9):633–643, Sep 2010. ISSN 1471-0080 (Electronic); 1471-0072 (Print); 1471-0072 (Linking). doi: 10.1038/nrm2957.

Dikla Raz-Ben Aroush, Noa Ofer, Enas Abu-Shah, Jun Allard, Oleg Krichevsky, Alex Mogilner, and Kinneret Keren. Actin turnover in lamellipodial fragments. *Current Biology*, 27(19):2963–2973.e14, 2017. doi: 10.1016/j.cub.2017.08.066.

Maryam Riaz, Marie Versaevel, Danahe Mohammed, Karine Glinel, and Sylvain Gabriele. Persistence of fan-shaped keratocytes is a matrix-rigidity-dependent mechanism that requires $\alpha5\beta1$ integrin engagement. *Scientific Reports*, 6(1):34141, 2016. doi: 10.1038/srep34141. URL `https://doi.org/10.1038/srep34141`.

Pierre Ronceray, Chase P Broedersz, and Martin Lenz. Fiber networks amplify active stress. *Proc. Natl. Acad. Sci. U. S. A.*, 113(11):2827–2832, March 2016.

Julie A. Theriot and Timothy J. Mitchison. Actin microfilament dynamics in locomoting cells. *Nature*, 352:126–131, 1991. doi: 10.1038/352126a0.

Sven K. Vogel, Zdenek Petrasek, Fabian Heinemann, and Petra Schwille. Myosin motors fragment and compact membrane-bound actin filaments. *eLife*, 2: e00116, 2013. doi: 10.7554/eLife.00116.
