# Supplementary material for: Balanced contractility and adhesion drive polarization in a minimal elastic actomyosin network
Source: PLoS Comput Biol. 2026 Sep 18;22(9):e1014750. doi: 10.1371/journal.pcbi.1014750 (PMC13630322; doi:10.1371/journal.pcbi.1014750)
Supplement: Supplementary file 4 [file pcbi.1014750.s014.pdf]

Recommendation: Minor Revisions

Summary of reviewer impression:

The authors have addressed most of the major and minor revisions to satisfaction, significantly clarifying the model and computational details, now employing consistent terminology across the manuscript as well as appropriately emphasizing novel aspects of the model/methods. In addition, the authors have added supplemental figures which illuminate the impact of model parameters not directly addressed in the main figures, strengthening the manuscript. Remaining comments are primarily regarding ensuring sufficient discussion of network pruning in the main text, and some minor comments of typos or small clarification.

Major Revision:

1. The additional supplementary Figs S2, S3, and S11 are all positive additions to the manuscript in that they reveal the degree of network pruning in phenotypes exhibiting migration as well as robustness of the model with respect to pruning parameters. Also, in the revised manuscript, the authors note the pruning mechanism mimics myosin-driven disassembly, so the pruning rules have a biophysical basis. However, the authors seem to shy away from emphasis on this mechanism, despite its prevalence in migratory cases. This is evident in, for example, Lines 89-90, in discussion of novel additions to the model: the authors note adhesion turnover and network growth, but not network disassembly via pruning which also adds to the dynamic nature of the model. Similarly in Line 260. where it is noted that the network is pruned, but not noting what this mimics in real actomyosin networks (as compared to Lines 264-265 which connects network growth to polymerization). The mention of Figs S2 and S3 is brief in the methods section, and not at all referenced in the Results or Discussion as to the role / presence of pruning in the various phenotypes. Meanwhile, Fig S11 is never mentioned in the main text. It is my recommendation that the authors make appropriate note of the merging/pruning rules in referencing the model, and discuss the extend of the impact of these rules on the results of this manuscript. For example, in the discussion section, the authors could summarize the points of Fig S2 and S3 and note how changing pruning parameters does not change the qualitative results, making reference to Fig S11.
    a. A related note: in Lines 107-108, the turnover, growth, and pruning rules are introduced as things which make the network “well-behaved”, secondly mentioning the biophysical motivation. I would swap the emphasis, as the rules all have a clear basis in the underlying biophysics and are connected to physiological scales.

Minor Revisions:

1. A couple remaining typos throughout the manuscript which should be addressed:
   i. Line 92: “of its energy to which contribute stretching/compression”…
   ii. In the revised manuscript, supplementary figures are often listed as “SS1”, “SS2”, etc. This should, presumably, be “S1”, “S2”, etc.
   iii. Line 77: “0.05%” or “0.05-4%”
   iv. Line 290 “correlation with force dipole” (presumably should be “dipole force”)
2. It would be beneficial for the authors to mention that the area cutoff of 1e5 units of cell area (in Line 269) corresponds to a typical cell size of 50 microns – this is indeed a reasonable cutoff, so mentioning the in-units value strengthens this choice for the reader.
3. I am confused how the trajectory gyration radius is calculated: what and when (in time) do r_i and r_m correspond to? This should be clarified in the revised manuscript.
4. In Fig 6A, it does not seem the dotted lines are defined in the figure caption (I assume this is related to the bound on the lifetime of an anchor due to the finite simulation time).
5. It would be nice if the authors added titles “migrating” and “erratic” to the top and bottom parts of Fig 6D.
