# Supplementary material for: Balanced contractility and adhesion drive polarization in a minimal elastic actomyosin network
Source: PLoS Comput Biol. 2026 Sep 18;22(9):e1014750. doi: 10.1371/journal.pcbi.1014750 (PMC13630322; doi:10.1371/journal.pcbi.1014750)
Supplement: Peer review history

# Peer review: Decision Letter 0

**DOI:** 10.1371/journal.pcbi.1014750.r001

**Authors:** Dimitrios Vavylonis, Jun Allard

9 Apr 2026

Balanced contractility and adhesion drive polarization in a minimal elastic actomyosin network

PLOS Computational Biology

Dear Dr. Messi,

Thank you for submitting your manuscript to PLOS Computational Biology. After careful consideration, we feel that it has merit but does not fully meet PLOS Computational Biology's publication criteria as it currently stands. Therefore, we invite you to submit a revised version of the manuscript that addresses the points raised during the review process.

Please submit your revised manuscript by Jun 09 2026 11:59PM. If you will need more time than this to complete your revisions, please reply to this message or contact the journal office at ploscompbiol@plos.org. When you're ready to submit your revision, log on to https://www.editorialmanager.com/pcompbiol/ and select the 'Submissions Needing Revision' folder to locate your manuscript file.

Please include the following items when submitting your revised manuscript:

* A letter that responds to each point raised by the editor and reviewer(s). You should upload this letter as a separate file labeled 'Response to Reviewers'. This file does not need to include responses to formatting updates and technical items listed in the 'Journal Requirements' section below.

* A marked-up copy of your manuscript that highlights changes made to the original version. You should upload this as a separate file labeled 'Revised Manuscript with Track Changes'.

* An unmarked version of your revised paper without tracked changes. You should upload this as a separate file labeled 'Manuscript'.

If you would like to make changes to your financial disclosure, competing interests statement, or data availability statement, please make these updates within the submission form at the time of resubmission. Guidelines for resubmitting your figure files are available below the reviewer comments at the end of this letter

We look forward to receiving your revised manuscript.

Kind regards,

Jun Allard

Guest Editor

PLOS Computational Biology

Dimitrios Vavylonis

Section Editor

PLOS Computational Biology

**Additional Editor Comments:**

The Reviewers are broadly positive about the importance and outcomes of the work, but they raise several issues, for example regarding the nature of the material and how it is added, removed and rearranged.

**Journal Requirements:**

If the reviewer comments include a recommendation to cite specific previously published works, please review and evaluate these publications to determine whether they are relevant and should be cited. There is no requirement to cite these works unless the editor has indicated otherwise.

1) Please ensure that the CRediT author contributions listed for every co-author are completed accurately and in full.

At this stage, the following Authors/Authors require contributions: Zeno Messi, Nathan Goehring, Franck Raynaud, and Alexander Verkhovsky. Please ensure that the full contributions of each author are acknowledged in the "Add/Edit/Remove Authors" section of our submission form.

The list of CRediT author contributions may be found here: https://journals.plos.org/ploscompbiol/s/authorship#loc-author-contributions

2) We ask that a manuscript source file is provided at Revision. Please upload your manuscript file as a .doc, .docx, .rtf or .tex. If you are providing a .tex file, please upload it under the item type u2018LaTeX Source Fileu2019 and leave your .pdf version as the item type u2018Manuscriptu2019.

3) Please upload all main figures as separate Figure files in .tif or .eps format. For more information about how to convert and format your figure files please see our guidelines:

https://journals.plos.org/ploscompbiol/s/figures

4) Please amend your detailed Financial Disclosure statement. This is published with the article. It must therefore be completed in full sentences and contain the exact wording you wish to be published.

1) State what role the funders took in the study. If the funders had no role in your study, please state: "The funders had no role in study design, data collection and analysis, decision to publish, or preparation of the manuscript."

2) If any authors received a salary from any of your funders, please state which authors and which funders..

If you did not receive any funding for this study, please simply state: u201cThe authors received no specific funding for this work.u201d

**Reviewers' comments:**

Reviewer's Responses to Questions

**Comments to the Authors:**

**Please note here if the review is uploaded as an attachment.**

Reviewer #1: In their manuscript the authors propose, based on previous experimental

and modeling studies, a minimal model for cell polarization and motility.

The model consists of elastic bonds, force dipoles mimicking myosin motor minifilaments

and anchors, mimicking adhesion bond formation and that break upon excess of load.

Simple turnover rules are applied for the dynamics.

Several phenotypes are found, including self-polarized moving "cells" of roughly constant size.

The main parameter is identified as the timescale of the anchor detachment.

The work suggests that this minimal system is sufficient for polarization, size determination

AND motion, which in my opinion is an important step in our understanding of motility.

Overall all I like the approach and find especially movie 5 impressive,

as it also recovers the shape and the transverse actomyosin bundle observed in crawling keratocytes.

The paper should be published after the authors respond to my comments below.

Comments:

1) There are quite some typos and formatting issues (e.g. p7 after eq(14) "(other refs?)").

Please fix them.

2) p8 after Eq(16) "In our simulations, when a single dipole is not

capable to bend a bond, the system simply grows"

This is hard to grasp at this point in the model description, since growth

was not yet mentioned. Maybe first describe the model parts AND the dynamics

very briefly before going into the details. This would help the reader

3) p9, line 140: "Merged nodes are replaced by a new node located at their barycenter

carrying all the bonds and dipoles of the pair"

I understand the procedure, and it makes sense. But I have the feeling

that this may lead to artificial "jumps" in the dynamics. For instance,

for simulations of growing tissue/bacterial colonies it is known that

replacing a cell by two (here it is rather the inverse, replacing many nodes

by a single one) leads to spurious and unrealistic peaks especially in the forces.

Did the authors check (eg for small systems) that such issues are not

important here?

4) Concerning the force maps described on p12, did the authors check

that the sum of all forces is always zero, as required for a force free system?

This should be mentioned/discussed.

5) p16 l306: here 5 phenotypes are mentioned but afterwards come only

three types with subtypes. Maybe restructure for clarity.

6) Concerning the isotropic/anisotropic growth discussed on page 17:

I find this phenotype also promising. It may be stabilized by some implementation

of mass conservation, right? Did the authors try this? Such a stabilization

would make the parameter region where polarization/motion is possible

substantially larger and the message of the manuscript even stronger

(limited resources/membrane are not needed for polarization, as the

authors also discuss in the conclusions, but may help it)

7) p18, l375 "contraction at the trailing edge not only balances the addition of new network

but leads to a steady translocation of the whole system"

Here, and also later on p28 l512 when UCSP model is mentioned,

the authors may consider to cite some continuum models

that also show that contraction at the rear

is sufficient for a bifurcation to motility, especially

Recho P, et al Phys. Rev. Lett. 2013, J. Mech. Phys. Solids 2015

and its generalization to adhesion, V Wössner, et al, New Journal of Physics 2024

8) As already stated above, I like the transverse actomyosin bundle very much.

The authors may add a figure with a quantification of this state,

when they discuss that "these structures are reminiscent of stress fibers"

on p19 l405.

9) I like the idea to also study external cues that can bias migration.

But at this point I find the introduction of a directional gradient in the ARTF ratio

a bit too oversimplified/phenomenological. For e.g. durotaxis, can one

not just implement a gradient in stiffness of the substrate springs or something similar?

The authors should try to be more realistic here or at least discuss a bit more thoroughly

how such changes of "real" parameters affect the ARTF.

10) In Fig3 and 4 the network displacement should be discussed/quantified more.

I see large displacements in eg Fig3F that seem not so much correlated with the dipoles/forces

in Fig3D, or maybe this is not so visible there. Can one quantify/correlate better?

11) In the results, the ratio of anchor removal threshold to dipole force (ARTF)

is discussed quite a bit but in the discussion the authors state

"The timescale of detachment effectively defines the behaviour of the system".

I suggest a discussion of ARTF is also needed in the discussion/conclusion

Reviewer #2: The paper by Messi and colleagues investigates cell polarization and migration. To do so, they construct a model of the cellular machinery used for migration. They include filaments (modeled based on actin filaments), force dipoles (myosin), and anchors (focal adhesions). The key behavior they encode in their model is force-dependent breakage of the anchors. They investigate the behavior of the model over ranges of parameter values that lead to different qualitative behaviors of the simulated cell.

Messi et al. find that in one extreme, when contractile forces are lower than the breaking strength of the adhesions, the system grows approximately isotropically. In the opposite extreme, when breaking strength is very low, adhesions do not persist and cells are also unable to move. Interestingly, in the middle regime, they find that the cell self-organizes into a polar, motile state. It is notable that no guiding cues or initial broken symmetry is necessary to create these states. These results are well presented and explained. I find these results interesting and valuable. I would note, however, that I am not an expert in the field of polarity establishment and motility, so am not well positioned to asses the novelty of this work within the context of similar models.

The model itself is of many movable nodes which can be connected by an energy function approximating the energy of a flexible filament. They can, in addition, be connected by an energy function approximating force generation by myosin. Finally, they can be attached elastically to a point in space, approximating adhesion to a substrate. Node positions for a given set of energetic links are determined by energy minimization. The links are then updated based on some rules, and energy is minimized again to model time evolution of the system. The energies used are reasonable, as is the procedure to simulate time through successive minimizations. The update rules, however, present a major issue with this work. Specifically, the update rules around removal of filaments and myosin links are presented as numerical concerns, but in my opinion require more careful treatment, as they encode key aspects of the biophysics of the model.

Major comments

1. The update rules specify that nodes which are close together are merged, then bonds removed if they number above 10 (as well as myosin links). Another rule specifies that bent links are removed when the bend is larger than some angle. These update rules appear to hide the biophysics of actin disassembly. The model should be reformulated to include a biophysical description of this process, and the behavior of the model checked across some range of the associated parameters.

a. The model has constant addition of material. The authors show that some systems converge to a constant cell area. The analysis of these systems is a key point of the paper. Do these systems also reach a steady state number of actin and myosin bonds? The answer to this question should be shown. In the case that they do, it would imply that the rate of actin loss by disassembly is equal to that of actin addition, making the two terms of equal importance to the behavior of the model. In the case that a steady state is not reached, this would imply that these systems are getting denser over time. In this case, the authors would need either to argue why studying such a system at early times is relevant, or add further disassembly rules to enable steady state density regimes.

b. From my reading of the model, the update rules basically encode actin disassembly which happens only at high concentrations and actin disassembly which happens when actin is compressed. What are their relative contributions to the total? Are actomyosin systems known to behave in this way? The biophysical model of disassembly should be put in context of what is known.

Minor comments

1. Equation 2 gives the energy of bending as a function of bend angle, with a sin^2 dependence. In the proceeding paragraph, there is a description of the bending energy of a rod with constant curvature. I was not able to see how the sin^2 term emerges from a constant curvature bend. The authors should briefly show how they arrive at this term.

2. In the model description, bonds are described as being able to be compressed, but also bent. I see no description for when compression is used vs. bending, or when bends are created.

3. It is strange to me that “consecutive bonds that are initially aligned can also form hinges”. Does this model something like a distribution of filament lengths? This choice would benefit from some explanation.

4. The meaning of point 2 in the pruning section is unclear. I gather from the later description that it is intended to mean the filament is removed from the system, but from the description in this point it is unclear what a “lower” angle means. Are hinges ever converted back to filaments?

5. Table 1 should be expanded to include references for the values, where applicable

6. The algorithm for placement of filaments, myosin links and anchors is not described

7. The threshold of 10^5 units of cell area is chosen as a limit for a “stable” cell size. In figure 1, the cells which are going above this limit are clearly already slowing down in their area growth. Why was this value chosen, and is it meaningful? The authors might consider the relationship of the cell growth rate with the rate of area addition instead.

8. The results of the paper would be strengthened by showing the hypothesis stated on line 357: that there exists a negative feedback between adhesion and contraction. The probability of nearby anchors to fail in the next iteration / time step could be compared to those far away.

9. In the text, it is described that “the measurement of anchor lifetime appears to reach a finite plateau. This is an artifact...”. I was not able to understand which feature of the data this line describes. The authors should perhaps reconsider how they include the anchors which never break into the measure of lifetime.

10. The way the paper approaches the relationship of the simulation with time needs to be consolidated. In figure 1, “iterations” is used to refer to what in other places is referred to as time or timestep. In the conclusion, a formal equivalence with time is stated and the timestep approximated in seconds. Consistent terminology should be used. It should also be clarified how the timescales of the various processes compare. Should, for example, we think of the mechanical equilibrium as fast compared to the chemical kinetics, which set the time evolution? Is mechanical equilibrium slow comparatively, dominating the timestep? In the discussion the protrusion rate is used to approximate the timestep, why is this the correct measure to pin the time evolution to instead of e.g. turnover or mechanics?

11. The final result is that a spatial bias in the ARTF ratio is sufficient to polarize the cell. In the methods I see this is implemented by changing the breaking threshold of the anchors. Is there a biological reason to think this might be the case in some form of cell migration? How might an external signal lead to a changing breaking threshold? What molecules might be involved? The discussion should be expanded.

12. The submitted draft of the manuscript has clearly not been sufficiently proofread (e.g. “(other refs.?)” in line 102, numerous typos and leftover sentence fragments in the methods section). This reviewer would point out that such sloppiness makes it doubly difficult to decipher the author’s intent and would ask the professional courtesy of the authors to do this work before asking others to evaluate the paper.

Notes on writing and presentation

1. In equations, variable subscripts etc. which are full words should be printed in plain text, not math italics (can be accomplished using LaTeX \text{} in the equation environment). Care should be taken to do this consistently throughout the text (e.g. they are mixed in the equation describing the persistency score)

2. Equation 1 assumes uniform bending, but this assumption is only introduced after the equation

3. In the title block, affiliation 4 is unused

4. There are too many typos in the methods section to list here, it needs to be gone through thoroughly

5. The first sentence of the author summary is awkwardly phrased and should be rewritten

6. Equation 8 overruns the page

7. In line 248, the anchors are described as “fixed-position nodes” but this contradicts the methods

8. Line 340: “is uniform and isotropic” should include an “approximately”

9. In figure 2 C, the authors present a schematic about trajectory reconstruction. The relationship of this to the message of the figure is unclear from the figure and legend. From the rest of the paper, I understand this to be connected to the construction of S_pers. In the current version of the figure, the placement makes it look like actual and reconstructed are related to the figures below, which I gather is not true. Panels c, d, and e should be rethought to better allow the reader to understand the message of the figure.

10. In the panels showing circular kimographs, a different color combo from green and red should be chosen, as red-green colorblindness is fairly common.

11. Typos

a. Line 8: “membrane ,”

b. Line 31: “description”

c. Line 40: “we extend discrete elastic model”

d. Line 41: “to dynamical case”

e. Line 386: missing paren

f. Line 513: unnecessary space

Reviewer #3: Review is uploaded as an attachment

**********

**Have the authors made all data and (if applicable) computational code underlying the findings in their manuscript fully available?**

The PLOS Data policy requires authors to make all data and code underlying the findings described in their manuscript fully available without restriction, with rare exception (please refer to the Data Availability Statement in the manuscript PDF file). The data and code should be provided as part of the manuscript or its supporting information, or deposited to a public repository. For example, in addition to summary statistics, the data points behind means, medians and variance measures should be available. If there are restrictions on publicly sharing data or code —e.g. participant privacy or use of data from a third party—those must be specified.

Reviewer #1: Yes

Reviewer #2: Yes

Reviewer #3: Yes

**********

PLOS authors have the option to publish the peer review history of their article (what does this mean?). If published, this will include your full peer review and any attached files.

If you choose “no”, your identity will remain anonymous but your review may still be made public.

**Do you want your identity to be public for this peer review?**  For information about this choice, including consent withdrawal, please see our Privacy Policy.

Reviewer #1: No

Reviewer #2: No

Reviewer #3: No

[NOTE: If reviewer comments were submitted as an attachment file, they will be attached to this email and accessible via the submission site. Please log into your account, locate the manuscript record, and check for the action link "View Attachments". If this link does not appear, there are no attachment files.]

**Figure resubmission:**

After uploading your figures to PLOS’s NAAS tool - https://ngplosjournals.pagemajik.ai/artanalysis, NAAS will process the files provided and display the results in the "Uploaded Files" section of the page as the processing is complete. If the uploaded figures meet our requirements (or NAAS is able to fix the files to meet our requirements), the figure will be marked as "fixed" above. If NAAS is unable to fix the files, a red "failed" label will appear above. When NAAS has confirmed that the figure files meet our requirements, please download the file via the download option, and include these NAAS processed figure files when submitting your revised manuscript.

**Reproducibility:**

To enhance the reproducibility of your results, we recommend that authors of applicable studies deposit laboratory protocols in protocols.io, where a protocol can be assigned its own identifier (DOI) such that it can be cited independently in the future. Additionally, PLOS ONE offers an option to publish peer-reviewed clinical study protocols. Read more information on sharing protocols at https://plos.org/protocols?utm_medium=editorial-email&utm_source=authorletters&utm_campaign=protocols

Attachment Submitted filename: PCOMPBIOL_04_2026_reviewer_comments.pdf

# Author comment: Author response to Decision Letter 1

**DOI:** 10.1371/journal.pcbi.1014750.r002

20 Jul 2026

Attachment Submitted filename: response_to_reviewers.pdf

# Peer review: Decision Letter 1

**DOI:** 10.1371/journal.pcbi.1014750.r003

**Authors:** Dimitrios Vavylonis, Jun Allard

20 Aug 2026

Dear Dr Messi,

We are pleased to inform you that your manuscript 'Balanced contractility and adhesion drive polarization in a minimal elastic actomyosin network' has been provisionally accepted for publication in PLOS Computational Biology.

Before your manuscript can be formally accepted you will need to complete some formatting changes, which you will receive in a follow up email. A member of our team will be in touch with a set of requests. In addition, Reviewer 2 and 3 have expended considerable care going through and finding typographical and language clarity improvements, which I suggest you give full consideration.

Please note that your manuscript will not be scheduled for publication until you have made the required changes, so a swift response is appreciated.

IMPORTANT: The editorial review process is now complete. PLOS will only permit corrections to spelling, formatting or significant scientific errors from this point onwards. Requests for major changes, or any which affect the scientific understanding of your work, will cause delays to the publication date of your manuscript.

Should you, your institution's press office or the journal office choose to press release your paper, you will automatically be opted out of early publication. We ask that you notify us now if you or your institution is planning to press release the article. All press must be co-ordinated with PLOS.

Thank you again for supporting Open Access publishing; we are looking forward to publishing your work in PLOS Computational Biology.

Best regards,

Jun Allard

Guest Editor

PLOS Computational Biology

Dimitrios Vavylonis

Section Editor

PLOS Computational Biology

***********************************************************

Reviewer 2 and 3 have expended considerable care going through and finding typographical and language clarity improvements, which I suggest you give full consideration.

Reviewer's Responses to Questions

**Comments to the Authors:**

**Please note here if the review is uploaded as an attachment.**

Reviewer #1: The authors have substantially revised their manuscript. Especially the algorithm is no much clearer,

but also some other presentation issues have been improved.

The authors have now carefully verified spurious forces and total force balance,

such that I am now fully convinced of their algorithm.

The new study concerning limited pools and added to the supplement strengthens the conclusions.

Overall, this is now a very nice work and I recommend publication.

Reviewer #2: The authors have greatly improved the understandability of the text by rewriting the methods section. Their responses have sufficiently addressed my major and minor comments.

I find no further issues which could make this work unsuitable for publication. Addressing the comments below may improve the presentation and readability of the of the work:

- In figures S2 and S3, "by number" is not easy to understand. Perhaps "by density cap"?

- The contributions detailed by figures S2 and S3 would benefit from some analysis in the text. These values seem to me to indicate that disassembly behaves differently in the three ARTF regimes.

- The reasoning about how time evolution is modeled as given in the response to my minor comment 10 would assist the reader if included in the methods section about the model.

- First sentence of author summary is a run on

- Line 52: The word optimal here would seem to mean "at one value", but I think the authors likely intend something like "within a range". Perhaps different word choice would be better.

- Line 91: "several cycles of minimization" seems to indicate that several cycles happen per iteration, the wording should be adjusted

- Line 104: "procedures" would be better as "iterations"

Reviewer #3: review is uploaded as an attachment

**********

**Have the authors made all data and (if applicable) computational code underlying the findings in their manuscript fully available?**

The PLOS Data policy requires authors to make all data and code underlying the findings described in their manuscript fully available without restriction, with rare exception (please refer to the Data Availability Statement in the manuscript PDF file). The data and code should be provided as part of the manuscript or its supporting information, or deposited to a public repository. For example, in addition to summary statistics, the data points behind means, medians and variance measures should be available. If there are restrictions on publicly sharing data or code —e.g. participant privacy or use of data from a third party—those must be specified.

Reviewer #1: Yes

Reviewer #2: Yes

Reviewer #3: Yes

**********

PLOS authors have the option to publish the peer review history of their article (what does this mean?). If published, this will include your full peer review and any attached files.

If you choose “no”, your identity will remain anonymous but your review may still be made public.

**Do you want your identity to be public for this peer review?**  For information about this choice, including consent withdrawal, please see our Privacy Policy.

Reviewer #1: No

Reviewer #2: No

Reviewer #3: No

Attachment Submitted filename: PCOMPBIOL_Rev_comments.pdf

# Editor report: Acceptance letter

**DOI:** 10.1371/journal.pcbi.1014750.r004

**Authors:** Dimitrios Vavylonis, Jun Allard

PCOMPBIOL-D-26-00527R1

Balanced contractility and adhesion drive polarization in a minimal elastic actomyosin

network

Dear Dr Messi,

I am pleased to inform you that your manuscript has been formally accepted for publication in PLOS Computational Biology. Your manuscript is now with our production department and you will be notified of the publication date in due course.

The corresponding author will soon be receiving a typeset proof for review, to ensure errors have not been introduced during production. Please review the PDF proof of your manuscript carefully, as this is the last chance to correct any errors. Please note that major changes, or those which affect the scientific understanding of the work, will likely cause delays to the publication date of your manuscript.

Soon after your final files are uploaded, unless you have opted out, the early version of your manuscript will be published online. The date of the early version will be your article's publication date. The final article will be published to the same URL, and all versions of the paper will be accessible to readers.

For Research, Software, and Methods articles, you will receive an invoice from PLOS for your publication fee after your manuscript has reached the completed accept phase. If you receive an email requesting payment before acceptance or for any other service, this may be a phishing scheme. Learn how to identify phishing emails and protect your accounts at https://explore.plos.org/phishing.

Thank you again for supporting PLOS Computational Biology and open-access publishing. We are looking forward to publishing your work!

With kind regards,

Janani Seenivasan

PLOS Computational Biology | Carlyle House, Carlyle Road, Cambridge CB4 3DN | United Kingdom ploscompbiol@plos.org | Phone +44 (0) 1223-442824 | ploscompbiol.org | @PLOSCompBiol
